# Exploring the complex spectrum of dominance and recessiveness in genetic cardiomyopathies

**DOI:** 10.1038/s44161-023-00346-3

**Published:** 2023-10-09

**Authors:** Alex Lipov, Sean J. Jurgens, Francesco Mazzarotto, Mona Allouba, James P. Pirruccello, Yasmine Aguib, Massimo Gennarelli, Magdi H. Yacoub, Patrick T. Ellinor, Connie R. Bezzina, Roddy Walsh

**Affiliations:** 1 Department of Experimental Cardiology, Heart Centre, Amsterdam UMC, Amsterdam, the Netherlands; 2 Amsterdam Cardiovascular Sciences, Heart Failure & Arrhythmias, Amsterdam, the Netherlands; 3 Cardiovascular Disease Initiative, Broad Institute of MIT and Harvard, Cambridge, MA USA; 4 Cardiovascular Research Center, Massachusetts General Hospital, Harvard Medical School, Boston, MA USA; 5 Department of Molecular and Translational Medicine, University of Brescia, Brescia, Italy; 6 National Heart and Lung Institute, Imperial College London, London, UK; 7 Aswan Heart Centre, Magdi Yacoub Heart Foundation, Aswan, Egypt; 8 Division of Cardiology, University of California, San Francisco, San Francisco, CA USA; 9 Genetics Unit, Istituto di Ricovero e Cura a Carattere Scientifico, Istituto Centro San Giovanni di Dio Fatebenefratelli, Brescia, Italy; 10 Harefield Heart Science Centre, Uxbridge, UK; 11 Demoulas Center for Cardiac Arrhythmias, Massachusetts General Hospital, Boston, MA USA; 12 European Reference Network for Rare and Low Prevalence Complex Diseases of the Heart, Amsterdam, the Netherlands

**Keywords:** Medical genetics, Medical genomics, Genetics research, Cardiovascular genetics

## Abstract

Discrete categorization of Mendelian disease genes into dominant and recessive models often oversimplifies their underlying genetic architecture. Cardiomyopathies (CMs) are genetic diseases with complex etiologies for which an increasing number of recessive associations have recently been proposed. Here, we comprehensively analyze all published evidence pertaining to biallelic variation associated with CM phenotypes to identify high-confidence recessive genes and explore the spectrum of monoallelic and biallelic variant effects in established recessive and dominant disease genes. We classify 18 genes with robust recessive association with CMs, largely characterized by dilated phenotypes, early disease onset and severe outcomes. Several of these genes have monoallelic association with disease outcomes and cardiac traits in the UK Biobank, including *LMOD2* and *ALPK3* with dilated and hypertrophic CM, respectively. Our data provide insights into the complex spectrum of dominance and recessiveness in genetic heart disease and demonstrate how such approaches enable the discovery of unexplored genetic associations.

## Abstract (web summary)

Lipov et al. performed a meta-analysis of biallelic genotypes in cardiomyopathy patients and the UK Biobank. Using rare variant association analysis, they identified 18 genes with robust evidence for recessive inheritance and revealed a complex spectrum of dominance and recessiveness.

## Main

The considerable genetic complexity of Mendelian diseases is increasingly recognized, with many conditions characterized by substantial genetic heterogeneity, incomplete penetrance, variable expressivity and sizable proportions of patients where causal variants are not identified. While Mendelian disease genes are typically categorized as being associated with either dominant or recessive inheritance, this is probably an oversimplification of the actual relationship between genotypes and clinical phenotype^1^. In particular, strict Mendelian definitions of dominance (where monoallelic and biallelic genotypes have similar phenotypes) and recessiveness (where heterozygous variants have no phenotypic effect) are unlikely to reflect the reality of human clinical genetics for most Mendelian diseases. Recent studies using large biobank datasets demonstrated that heterozygous carriers of known pathogenic biallelic variants often display evidence of mitigated or milder phenotypes that are typically related to the recessive condition (such as associations with asthma, bronchiectasis and aspergillosis in carriers of the cystic fibrosis variant *CFTR* p.Phe508del)^2,3^. These observations indicate that variations on a semidominant model may better represent the more complex genetic architecture of many Mendelian-like disease genes^3^.

Cardiomyopathies (CMs) are heritable cardiac conditions that are leading causes of heart failure (HF) and sudden cardiac death (SCD). The most common subtypes are hypertrophic (HCM), dilated (DCM) and arrhythmogenic (ACM), which collectively affect approximately 1 in 200 people. Over the past three decades, investigations into the genetic basis of CMs have progressed from initial linkage-based study gene discovery through the candidate gene era and subsequently extensive reevaluation to define robust gene–disease associations, particularly through the ClinGen curations for HCM^4^, DCM^5^ and ACM^6^. The vast majority of genes validated through this process were evaluated for, and are associated with, autosomal dominant inheritance, which represents the major inheritance mode for CMs but also perhaps partly reflects the concentration of genomics research in outbred European ancestry populations. Since these initial re-curation activities, there has been a sharp rise in the number of proposed new gene associations with recessive CM (even as new dominant associations plateaued), driven by increased accessibility to exome and genome sequencing, research studies in previously understudied populations and cohort analyses of patients with pediatric CM^7–10^.

In CM genetics, biallelic pathogenic variants in genes associated with supposed autosomal dominant inheritance are rare, but have been consistently reported for many years; they are usually associated with a more severe phenotype or earlier disease onset, for example, biallelic loss-of-function (LOF) variants in *MYBPC3* observed in severe neonatal presentation of disease. Similarly, the most established CM gene with early-onset recessive inheritance, *ALPK3* (ref. ^11^), was subsequently shown to cause adult-onset HCM in heterozygosity^12^. Such observations suggest that dosage effects may be commonly observed in CM genes, supporting a semidominant-type model. However, detailed understanding of genotype–phenotype correlations for each disease gene, which is essential for the effective interpretation of clinical genetic data, remains sparse.

In this study, we undertake a comprehensive meta-analysis of biallelic variation associated with CM phenotypes to define genes robustly associated with recessive disease, investigate the continuum of inheritance and effect sizes across CM genes and identify putative monoallelic associations in these genes using biobank datasets. These findings highlight the increasing genetic heterogeneity and complex spectrum of dominance and recessiveness in heritable cardiac conditions and will enable more informed and equitable application of clinical genetic testing.

## Results

**Fig. 1:**
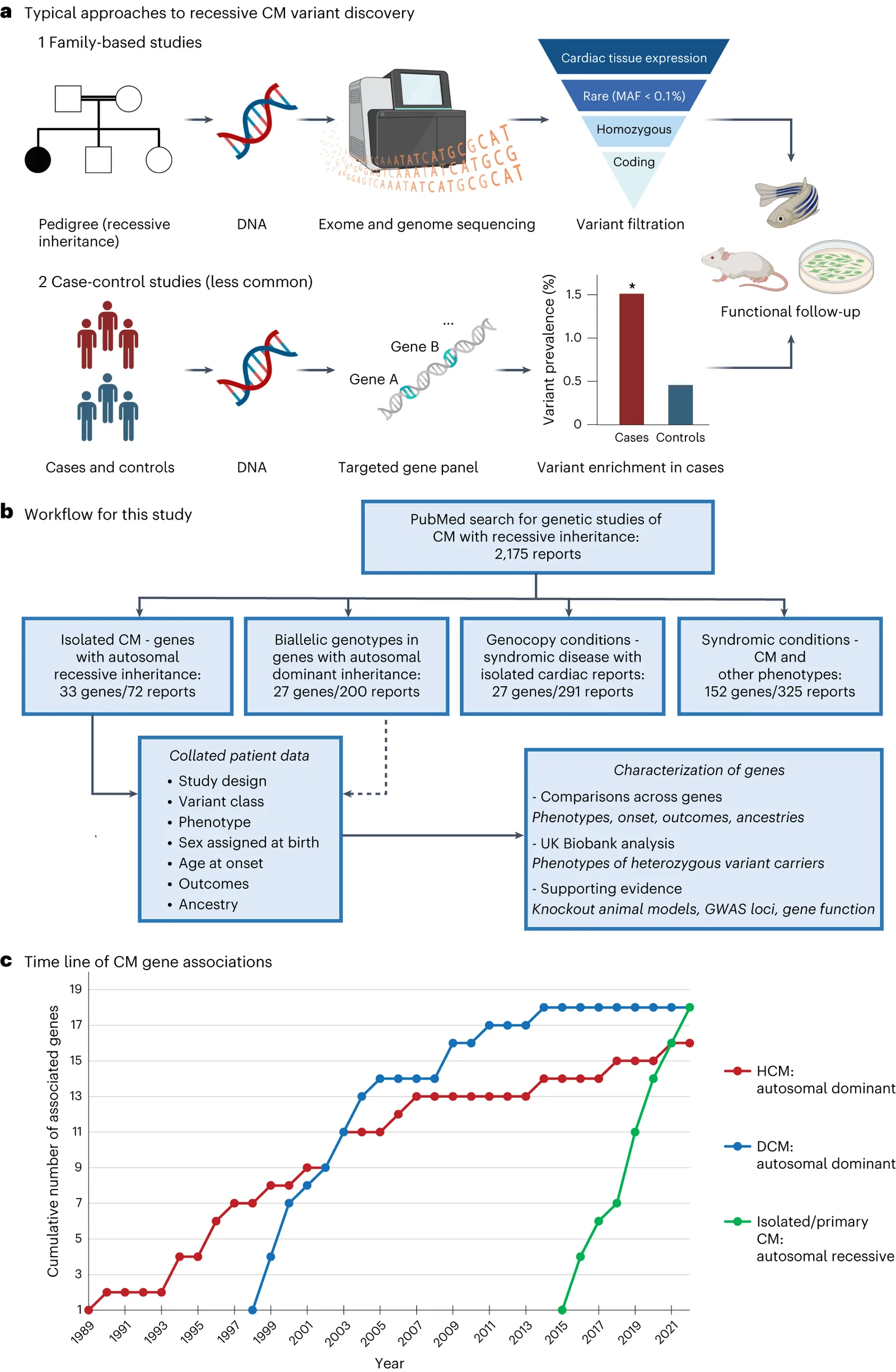
The identification of genes associated with recessive CM. **a**, Standard approaches to identify genes and variants associated with recessive inheritance based on the published studies evaluated in this analysis. **b**, Workflow for this meta-analysis study including the number of distinct genes and reports for the four categories of genes associated with biallelic and recessive inheritance of CM phenotypes. **c**, Time line of robust gene–disease associations for CMs, comparing autosomal dominant inheritance genes for HCM and DCM (based on ClinGen gene–disease curation) with autosomal recessive inheritance genes for all CMs (robustly associated genes as defined in this study).

A PubMed search with terms for CMs and recessive inheritance yielded 2,175 results (search restricted to English-language non-review articles published between 1990 and 2023, search date 7 June 2023; see Methods for the full search terminology), with a further 29 relevant reports identified through referral in these papers. Abstracts were triaged to identify human clinical reports where autosomal biallelic variants were reported for CM phenotypes. Selected articles were then categorized into four groups; (1) genes associated with recessive inheritance for isolated CM phenotypes (33 genes in 72 reports; Supplementary Table 1); (2) reported biallelic genotypes in CM genes with established autosomal dominant inheritance (27 genes in 200 reports); (3) potential genocopy genes for recessive syndromic diseases where patients presented with isolated CM (27 genes in 291 reports; Supplementary Table 2); and (4) recessive syndromic conditions that include CM as part of multisystem disease but without reported isolated presentation (152 genes in 325 reports; Supplementary Table 3). Most of the 72 reports for recessive isolated CM were family-based studies using genome-wide approaches (exome and genome sequencing), augmented by a small number of case-control analyses and pediatric cohort sequencing studies (Fig. 1a). The workflow for this analysis is shown in Fig. 1b.

### Recessive CM genes

For genes associated with recessive CM (group 1), we considered associations to be robust if multiple (≥2) families with biallelic variants identified through genome-wide methods were reported (excluding genes where evidence was derived solely from cohort sequencing studies) or significant enrichment of such variants was demonstrated in case versus control cohorts. The phenotypes described were either isolated CMs or patients where the cardiac phenotype was consistently the presenting or primary clinical symptom. These associations are further supported by additional evidence described below and summarized in Table 1. For each gene, full details of the studies, variants and patient clinical information are described in Supplementary Table 1, with detailed text summaries of the published evidence in the Supplementary Text (see Methods for additional details of the curation approach).

**Table 1:** Summary of 18 genes associated with autosomal recessive CM with robust evidence

Gene	Gene function	Phenotype	Reports	Families	Biallelic cases	Variant classes	Evidence summary	GWAS associations	Mouse knockout phenotypes
*ALPK3*	Localization of myomesin (M-band and nucleus)	DCM/HCM	9 (refs. ^11,52–59^)	17	26	PTV/missense	Exome (8 trio, 5 proband). Panel (2 trio, 2 proband). Max LOD = 2.9 (ref. ^52^)	HCM, LV/ECG traits	HCM and DCM features
*BAG5*	Co-chaperone of proteostasis regulation	DCM	2 (refs. ^60,61^)	5	6	PTV	Exome (3 trio, 2 proband)	–	LV dilation and arrhythmogenicity
*CAP2*	Thin filament protein (actin regulation)	DCM	3 (refs. ^62–64^)	3	4	PTV	Exome (2 trio, 1 proband). Max LOD = 1.9 (ref. ^62^)	–	Severe DCM, conduction anomalies, sudden death
*FBXO32*	E3 ubiquitin ligase subunit	DCM	2 (refs. ^65,66^)	2	6	Missense	Exome (2 trio). Max LOD = 3.4 (ref. ^65^)	HCM, AF, ECG traits	–
*FLII*	Actin remodeling protein	DCM	2 (refs.^10,67^)	3	3	PTV/missense	Exome (3 trio)	–	Embryonic lethality
*JPH2*	Junctional membrane complex	DCM	6 (refs. ^7,8,10,68–70^)	7	7	PTV/missense	Exome (2 trio, 2 proband). Panel (1 trio, 2 proband)	–	Embryonic lethality
*KLHL24*	E3 ubiquitin ligase substrate adapter	HCM	3 (refs. ^10,71,72^)	4	9	PTV/missense	Exome (3 trio, 1 proband). Max LOD = 3.6 (ref. ^71^)	–	–
*LDB3*	Z-disc protein	DCM	1 (ref. ^73^)	5	5	PTV	Exome (5 trio)	ECG traits	Severe DCM, early death
*LEMD2*	Inner nuclear membrane protein	ACM	1 (ref. ^74^)	2	11	Missense	Exome (2 large pedigrees). Max LOD = 7.3 (estimated)^74^	–	DCM-like phenotype with fibrosis and arrhythmia (p.Leu13Arg knock-in)
*LMOD2*	Thin filament protein (actin elongation)	DCM	5 (refs. ^75–79^)	5	6	PTV	Exome (4 trio). Panel (1 trio)	–	DCM-like phenotype, early death
*MYZAP*	Intercalated disc protein	DCM	2 (refs. ^80,81^)	3	8	PTV	Exome (3 trio)	AF	TAC-induced hypertrophy, HF, increased mortality
*NRAP*	Intercalated disc protein	DCM	10 (refs. ^7,10,19,61,72,82–86^)	30	32	PTV/missense	Exome (7 trio, 12 proband). Panel (2 trio, 9 proband). RVAS: 1.9% versus 0% (*P* < 0.00001)^19^	ECG traits	–
*PLEKHM2*	Kinesin transport cargo adapter protein	DCM	2 (refs. ^87,88^)	2	5	PTV	Exome (1 trio). Panel (1 trio). Max LOD = 2.8 **(estimated)**^87^	LV traits	–
*PPA2*	Inorganic pyrophosphatase	DCM/SCD	7 (refs. ^7,8,89–93^)	32	53	Missense	Exome (21 trio, 3 proband). Panel (8 trio)	–	–
*PPP1R13L*	NF-κB and p53 inhibitor	DCM	6 (refs. ^9,10,94–97^)	11	15	PTV/missense	Exome (6 trio, 5 proband)	–	Severe and rapidly progressing DCM
*RPL3L*	Muscle-specific ribosomal protein	DCM	4 (refs. ^10,98–100^)	6	9	Missense	Exome (6 trio)	AF	–
*SLC30A5*	Zinc transporter	CM	1 (ref. ^101^)	2	4	PTV	Exome (2 trio)	–	Sudden death (60%)
*TRIM63*	E3 ubiquitin ligase	HCM	6 (refs. ^25,102–106^)	28	32	PTV/missense	Panel (7 trio, 21 proband). RVAS: 0.4% versus 0% (*P* = 0.0002)^104^, 2.1% versus 0% (*P* = 0.003)^25^	Strain, ECG traits	Severe hypertrophy (TAC or *TRIM55* double-knockout)

For the evidence summary, cases are summarized as ‘exome’ (variants detected with genome-wide methods of genome and exome sequencing) or ‘panel’ (sequencing of distinct sets of CM-associated genes only), and as ‘trio’ (confirmed recessive inheritance with heterozygous parents) or ‘proband’ (recessive inheritance not confirmed but likely). The summary also includes the maximum reported or estimated LOD scores for large family pedigrees and details of the RVAS enrichment analysis. See the Supplementary Text and Methods for further details and citations for this genetic evidence, GWAS associations and mouse knockout phenotypes. Strain, myocardial strain in longitudinal direction; TAC, transverse aortic constriction.

We identified 18 genes with robust association with recessive CMs. Of these, 12 are associated with DCM (*BAG5*, *CAP2*, *FBXO32*, *FLII*, *JPH2*, *LDB3*, *LMOD2*, *MYZAP*, *NRAP*, *PLEKHM2*, *PPP1R13L* and *RPL3L*), 2 with HCM (*KLHL24* and *TRIM63*) and 4 with mixed or other CM phenotypes (*ALPK3*, *LEMD2*, *SLC30A5* and *PPA2*) (Table 1). Of note, all of these associations were proposed from 2015 onwards, during which time robust associations for dominant inheritance had largely plateaued (Fig. 1c). In addition to the demonstration of recessive inheritance using genome-wide methods, the evidence includes strong logarithm of odds (LOD) scores in large family pedigrees (*ALPK3*, *FBXO32*, *KLHL24*, *LEMD2*, *PLEKHM2*), the enrichment of rare biallelic variants in case versus control cohorts (*NRAP*, *TRIM63*) and additional cases where, although recessive inheritance has not been definitively demonstrated, patients are highly likely to have biallelic genotypes (Table 1 and Supplementary Text). Notably, no homozygous individuals for any of the implicated variants are described in the Genome Aggregation Database (gnomAD) v.2.

**Extended Data Fig. 1:**
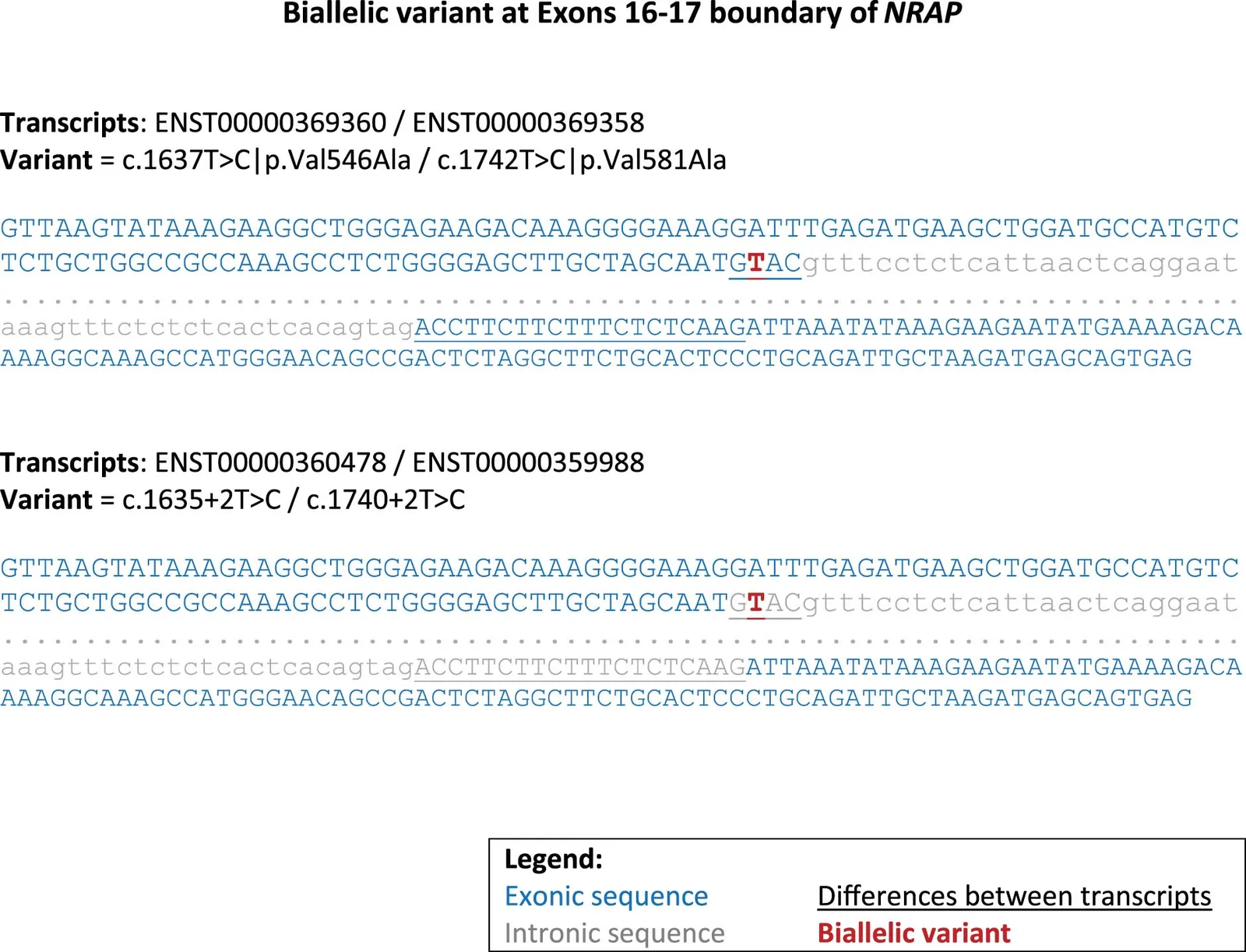
Predicted effect of homozygous *NRAP* variant in different transcripts. The homozygous *NRAP* variant GRCh37:chr10-115391614-A-G was detected in one individual in the cohort of 3222 British adults of Pakistani heritage by Narasimhan et al.^14^ (highlighted in red). The variant affects a splice donor site in two transcripts (ENST00000360478:c.1635+2 T > C / ENST00000359988:c.1740+2 T > C) but acts as missense variant in two others (ENST00000369360:p.Val546Ala / ENST00000369358:p.Val581 Ala). This is due to differential splicing of 24 bases (underlined) in the intron between exons 16 and 17. Therefore, although annotated as a potential loss-of-function variant, its effect is expected to be much less severe than most truncating variants in *NRAP* due to the milder missense variant effect in two of the NRAP transcripts.

The association of several of these genes with CM is further supported by concordant phenotypic data in mouse homozygous knockouts (Table 1). Such animal models are directly comparable for the 13 genes where biallelic protein-truncating variants (PTVs) (that is, nonsense, frameshift and splice donor or acceptor variants) were the sole or predominant variant class in patients with CM. Additionally, only one high-confidence homozygous PTV in these 13 genes was detected in 650,979 individuals from the gnomAD, UK Biobank and cohorts with Pakistani ancestry^13,14^ (which is unlikely to be a bona fide LOF variant; Extended Data Fig. 1); no predicted compound heterozygous PTVs were detected in 578,392 individuals in gnomAD v.2 and the UK Biobank (Methods and Supplementary Table 4). Some genes are also putative causal genes at cardiac-relevant genome-wide association study (GWAS) loci associated with CM or left ventricular (LV) traits (*ALPK3*, *FBXO32*, *PLEKHM2*, *TRIM63*), atrial fibrillation (AF) (*FBXO32*, *MYZAP*, *RPL3L*) and electrocardiogram (ECG) traits (*ALPK3*, *FBXO32*, *LDB3*, *NRAP*, *TRIM63*) (Table 1).

**Fig. 2:**
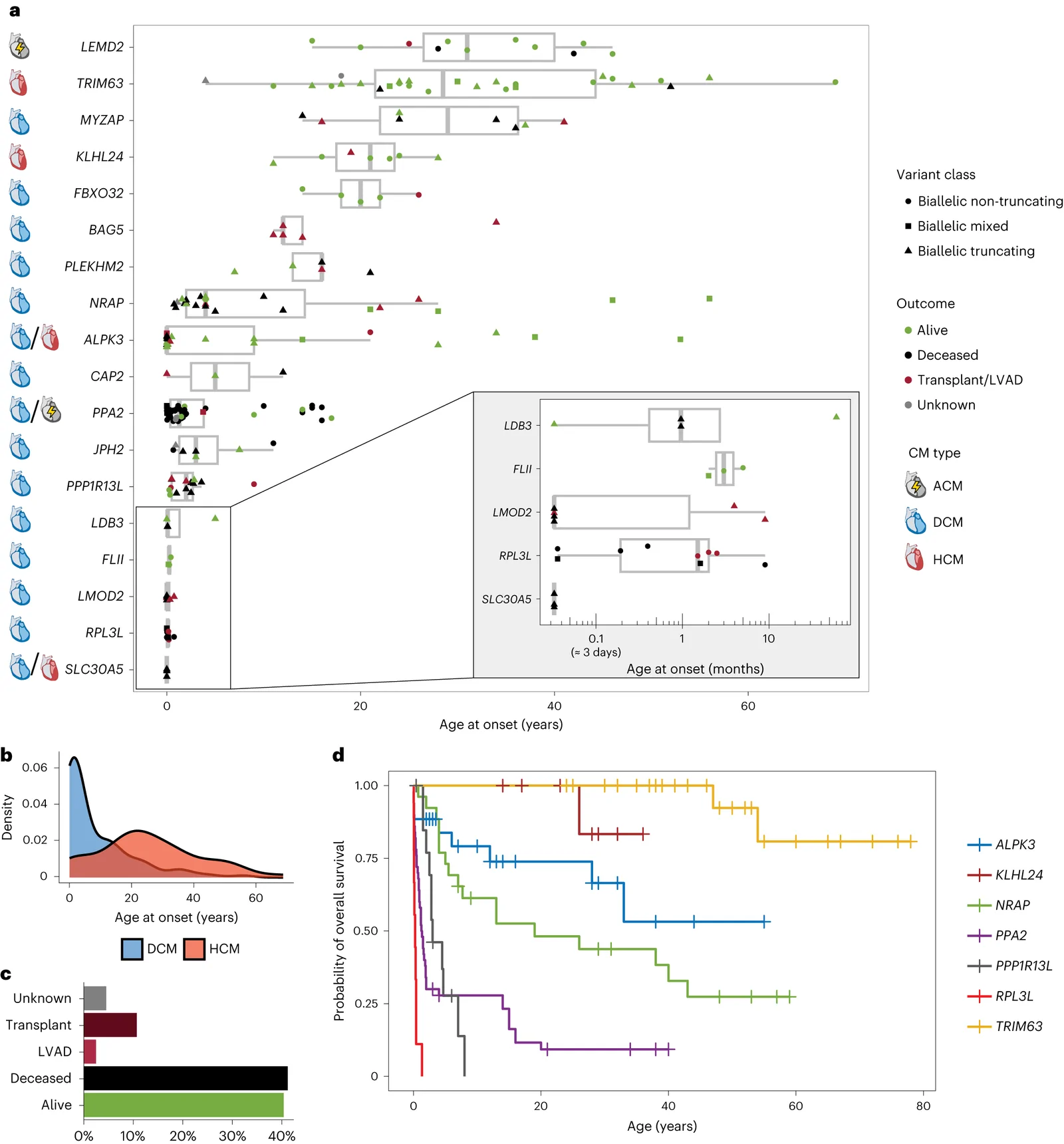
Patients with biallelic variants in robustly associated recessive CM genes. **a**, Summary of clinical details for patients with recessive CM (*n* = 241), including age at onset (*x* axis), outcome (dot color) and variant class (dot shape) as shown in the legend. For each gene, the primary CM phenotype is also shown. Data are presented as box plots with the median, an IQR box (25th to 75th percentile) and whiskers from the minimum to the maximum, excluding outliers (defined as >1.5× the IQR). **b**, Age at onset density plots for cases with biallelic variants in unambiguous DCM (*n* = 107) and HCM (*n* = 50) genes, highlighting that HCM genes (*KLHL24* and *TRIM63*) are associated with later onset (mean onset: 8.5 versus 25.4 years, *P* = 5.04 × 10^−11^, two-tailed *z*-test). **c**, Overall outcomes for all 241 cases. **d**, Kaplan–Meier survival curves for seven genes with more than eight cases with outcome data, with survival defined as freedom from death, heart transplantation or insertion of an LVAD. Relative to *NRAP*, the hazard ratios (HRs) for these genes were: *TRIM63*: 0.07 (0.02–0.29), *P* = 0.00031; *ALPK3*: 0.59 (0.25–1.38), *P* = 0.22; *PPA2*: 3.72 (2.05–6.75), *P* = 1.6 × 10^−5^; *PPP1R13L*: 3.01 (1.34–6.73), *P* = 0.0074; *RPL3L*: 21.0 (8.25–53.7), *P* = 1.8 × 10^−10^; *KLHL24*: 0.16 (0.02–1.22), *P* = 0.077 (Cox proportional hazards regression).

**Extended Data Fig. 2:**
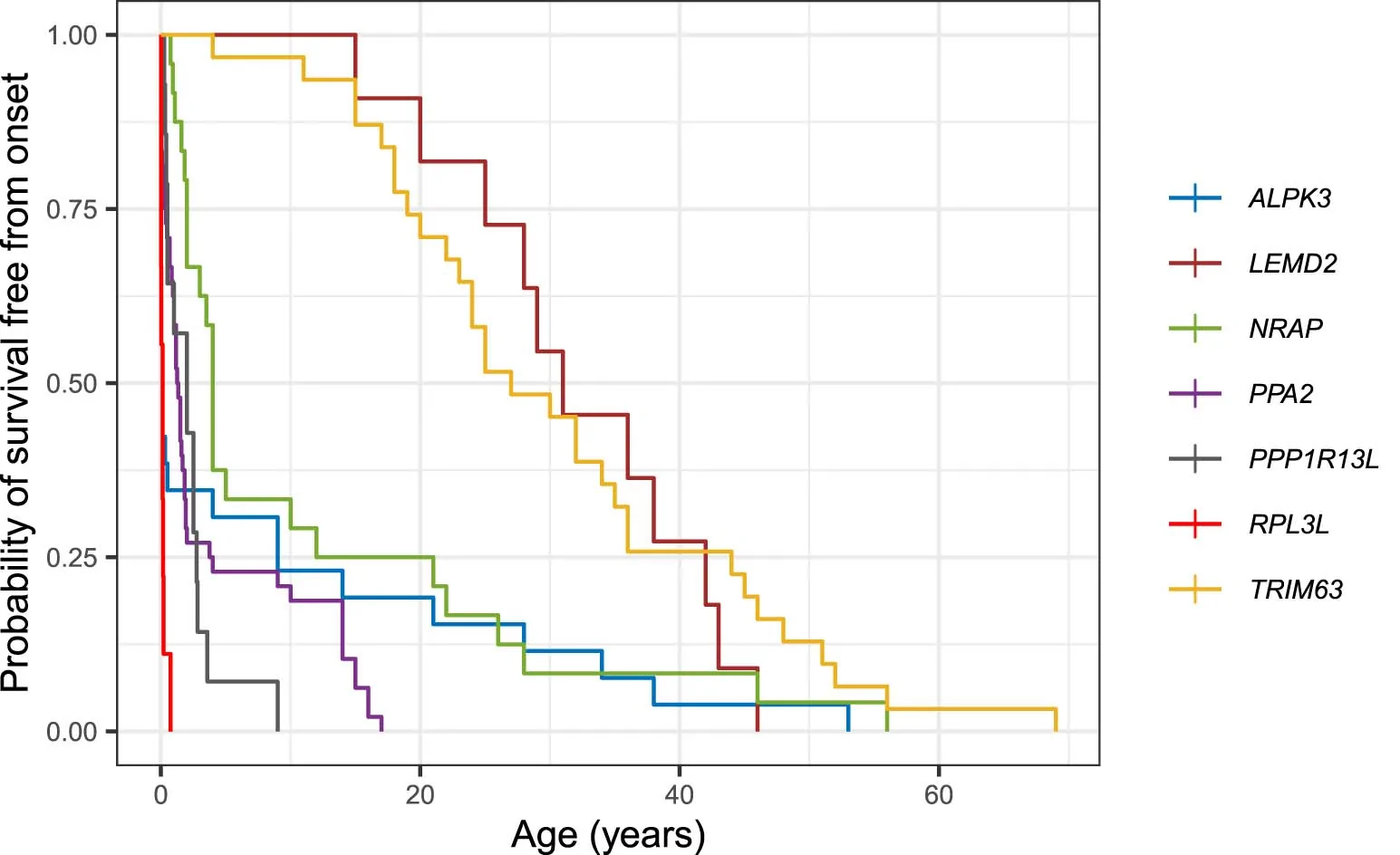
Outcome analysis for carriers of biallelic variants in genes associated with recessive cardiomyopathy. Kaplan-Meier curves for 7 genes with n > 8 cases with age of onset data, with survival defined as freedom from onset of disease. Relative to *NRAP*, hazard ratios for these genes were: *ALPK3* 1.54 (0.88-2.71), p = 0.13; *LEMD2* 0.43 (0.21-0.89), p = 0.023; *PPA2* 2.90 (1.71-4.91), p = 7.8e-5; *PPP1R13L* 3.21 (1.59-6.46), p = 0.0011; *RPL3L* 11.2 (4.71-26.5), p = 4.5e-8; *TRIM63* 0.41 (0.24-0.71), p = 0.0014 (Cox proportional hazards regression).

As expected for recessive inheritance, age at disease onset for these patients is generally lower compared to autosomal dominant CM. For HCM, recessive cases had a mean of 25.4 ± 16.4 years, median 24.0 (interquartile range (IQR) = 15.3–34.8) years (Extended Data Table 1) compared to a mean of 37.3 ± 17.1 years, median 37.5 (IQR = 23.6–49.8) years for genotype-positive HCM cases of the SHaRe registry^15^ (*P* = 2.4 × 10^−7^, two-tailed *z*-test). For DCM, recessive cases had a mean of 8.5 ± 11.4 years, median 3.3 (IQR = 0.5-13.5) years (Extended Data Table 1) compared to a mean of 29.8 ± 20.9 years, median 32.0 (IQR = 12.5-48.5) years for genotype-positive DCM cases from the Pugh et al. cohort^16^ (*P* = 4.9 × 10^−15^, two-sided Wilcoxon rank-sum test with continuity correction). However, there was considerable variation in age at onset between, and indeed within, genes, ranging from largely adult-onset disease (for example, *TRIM63* and *MYZAP*) to severe neonatal and pediatric disease phenotypes (for example, *RPL3L* and *LMOD2*) (Fig. 2a and Extended Data Fig. 2). In general, DCM phenotypes were associated with a significantly earlier age at onset compared to HCM (Fig. 2b). In contrast to autosomal dominant CMs, which have a strong male predominance (60–70% male for HCM and DCM^15,17^), there was a closer sex balance for recessive cases (45.2% female) suggesting higher penetrance for biallelic variants. Overall, the reported outcomes revealed a severe disease course for many of these genotypes, with death reported in 41.5% of cases and heart transplantation or LV assist device (LVAD) implantation in 13.3% of cases (Fig. 2c). We performed outcome analysis for seven genes with *n* > 8 cases with available outcome data (Fig. 2d), which again demonstrated considerable variability in disease severity. Patients with biallelic variants in *TRIM63* and *KLHL24* had a relatively mild disease course, *PPP1R13L*, *PPA2* and *RPL3L* were associated with lethal outcomes at young ages, while *ALPK3* and *NRAP* cases had intermediate outcomes despite often presenting as pediatric CM.

**Fig. 3:**
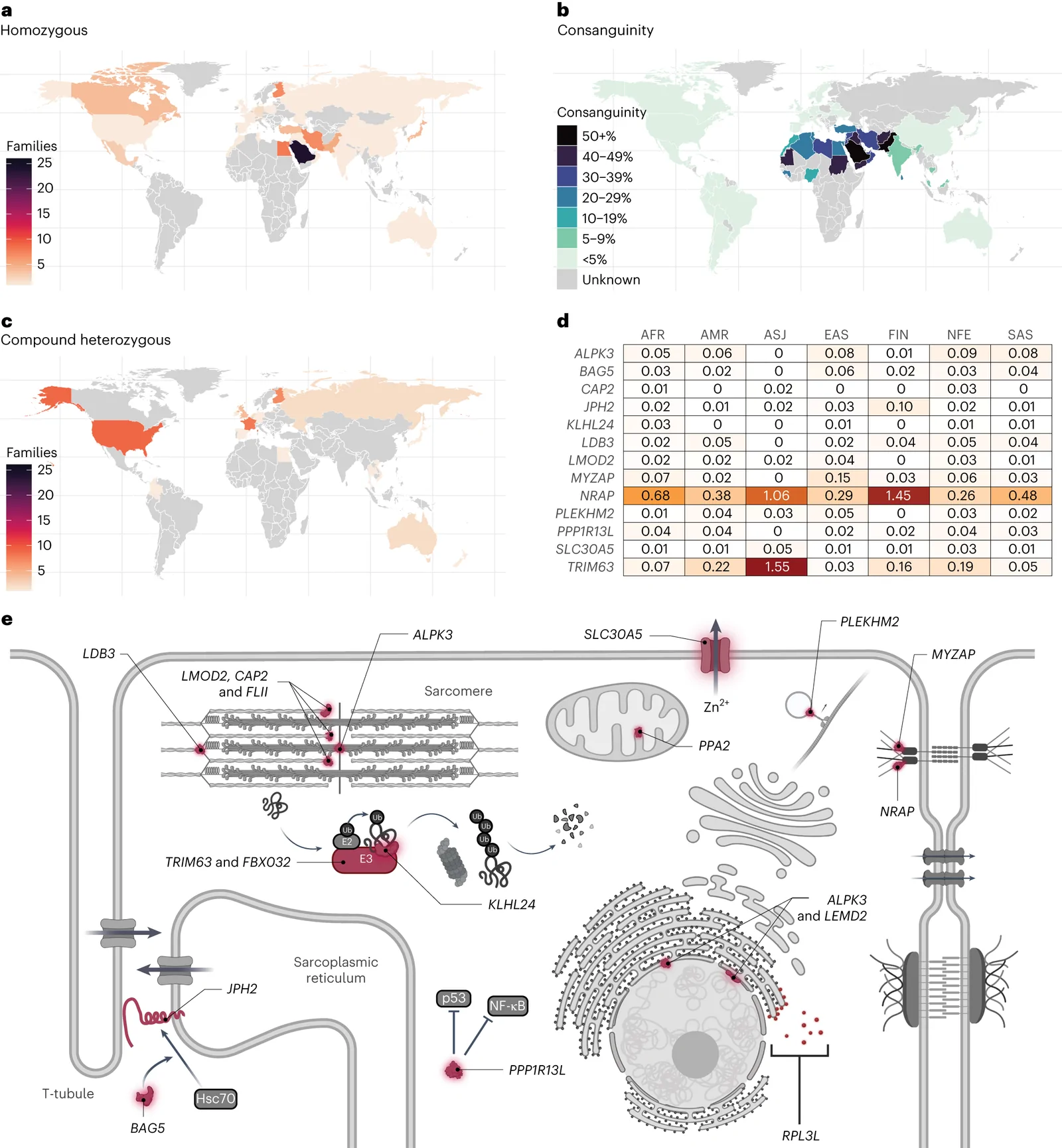
Effect of ancestry on the biallelic genotypes and functions of genes associated with recessive CM phenotypes. **a**, Countries of origin for families with homozygous variants in the 18 genes robustly associated with autosomal recessive cardiomyopathy, demonstrating enrichment in countries with isolated populations (e.g. Finland, Japan) and high consanguinity rates. **b**, Global consanguinity rates (data from Hamamy et al.^107^). **c**, Countries of origin for families with compound heterozygous variants in the 18 genes robustly associated with autosomal recessive CM more typically reflect traditional centers of genetic research. **d**, Percentage of individuals in the gnomAD population groups with LOF variants in genes robustly associated with autosomal recessive CM and where PTVs were the sole or predominant variant class. AFR, African; AMR, Admixed American; ASJ, Ashkenazi Jewish; EAS, East Asian; FIN, Finnish; NFE, non-Finnish European; SAS, South Asian. **e**, Cellular functions and locations for the proteins of the 18 genes robustly associated with autosomal recessive CM.

The countries of origin of families with biallelic CM genotypes are highlighted in Fig. 3. As expected, patients with homozygous variants are enriched in population bottlenecks where founder variants are prevalent (for example, Finland) and in countries of high consanguinity (Fig. 3a–c). For recessive genes where PTVs were the sole or predominant variant class in patients with CM, we calculated the percentage of individuals with such variants in the heterozygous state for the major population groups in the gnomAD database (Fig. 3d). These data indicate the likely relative prevalence of recessive CM across different genes and population groups, assuming random mating. Stark differences are observed between population groups for some genes, with bottleneck populations like Finns and Ashkenazi Jews in particular harboring founder variants that make certain genes more prevalent causal factors in these populations. Conversely, for genes without founder variants, these populations tend to have a lower overall prevalence of ultrarare variants^18^. For example, the *TRIM63* variant p.Gln247* has an allele frequency of 0.008 in Ashkenazi Jews, suggesting it may be a relatively common cause of HCM in this population. A study by Blueprint Genetics in Finland detected biallelic variants in *NRAP* in up to 2% of studied DCM cases, which may reflect the particular enrichment of *NRAP* PTVs in Finns^19^, although the gene also accounted for 14.3% of all pediatric DCM cases in a Saudi Arabian cohort^10^.

These 18 robustly associated recessive disease genes encode for proteins with a wide range of functions, helping to expand our knowledge of the biological pathways and processes underlying CM pathophysiology (Table 1 and Fig. 3e). The functions include sarcomeric proteins of the thin filament (encoded by *LMOD2*, *CAP2*, *FLII*) and Z-disc (encoded by *LDB3*), intercalated disc proteins (encoded by *MYZAP* and *NRAP*) and components of the ubiquitin proteasome pathway (encoded by *FBXO32*, *KLHL24* and *TRIM63*).

A further 13 genes were reported for recessive CM phenotypes but have thus far been identified in single families only (*AASDH*, *ACACB*, *BICD2*, *CASZ1*, *GATAD1*, *GET3*/*ASNA1*, *KIF20A*, *PHACTR2*, *SLC6A6*, *SOD2*, *TAF1A*, *ULK1*) or in multiple probands solely in a large cohort study (*RHBDF1*, three cases)^10^ (Supplementary Table 1 and Extended Data Table 2). While these studies also used unbiased genome-wide approaches (with some genes supported by animal model and other evidence), replication in additional patients with CM is required to validate these associations. Two other genes (*GJB4* and *LRRC10*, implicated in severe early-onset disease) were excluded from the lists of potential genes because of the presence of multiple adult individuals in gnomAD homozygous for the single implicated missense variants (Methods).

### Biallelic variants in established dominant genes

Our literature search returned reports on biallelic variants for 27 genes with established autosomal dominant association for CMs (that is, genes with definitive, strong or moderate association based on ClinGen curation^4–6^). Some of these relate to well-characterized recessive conditions, for example, the cardiocutaneous Naxos and Carvajal syndromes associated with biallelic variants in desmosome genes, and skeletal myopathy diseases associated with biallelic variants in DCM genes like *TTN* and *LMNA*. In this study, we focused our analysis on sarcomeric CM genes to explore the spectrum of dominance and recessiveness in genes of predominantly dominant inheritance. Such analysis is complicated by certain confounding factors: biallelic variants with lethal phenotypes are probably absent from clinical records (survivor bias) and missense variants (the most prevalent pathogenic variant class for most sarcomeric genes) can vary widely in the severity of their effect. Nevertheless, through the meta-analysis of all published cases and incorporation of related analyses, we identified and characterized distinct genotype–phenotype associations across different genes and variant classes.

**Fig. 4:**
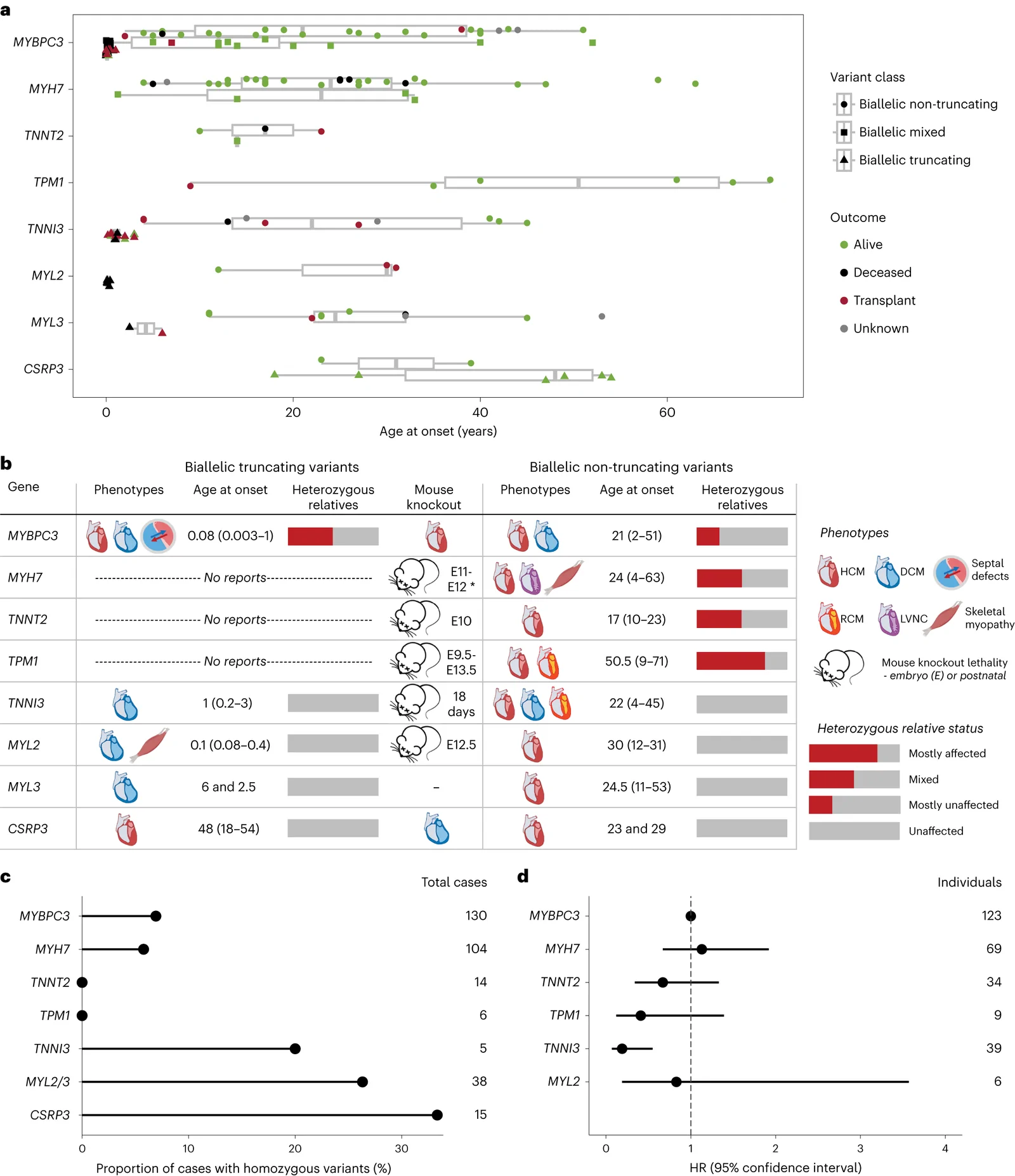
Biallelic variation in genes largely associated with autosomal dominant CM. **a**, Age at onset for cases with biallelic variants in sarcomeric HCM (and DCM) genes usually associated with autosomal dominant inheritance (*n* = 170), shaped and grouped according to variant class and colored according to outcome (as shown in the legend). Data are presented as box plots with the median, an IQR box (25th to 75th percentile) and whiskers from the minimum to the maximum, excluding outliers (defined as >1.5× the IQR). **b**, Summary of biallelic variant associations for these genes (for truncating and non-truncating variants), displaying typical phenotypes, age at onset (median and range), likelihood of phenotype expression in heterozygous relatives and mouse knockout phenotypes, including age at death where lethal (full clinical details are shown in Supplementary Table 5). LVNC, left ventricular non-compaction; RCM, restrictive CM. * data for *Myh6*, the functionally equivalent gene of *MYH7* in mice. **c**, Proportion of Egyptian HCM probands where variants occur in homozygosity, per gene (Allouba et al.^25^). **d**, HRs for penetrance of HCM in sarcomeric variant carriers identified using family screening, compared to *MYBPC3* as baseline (Lorenzini et al.^26^).

**Extended Data Fig. 3:**
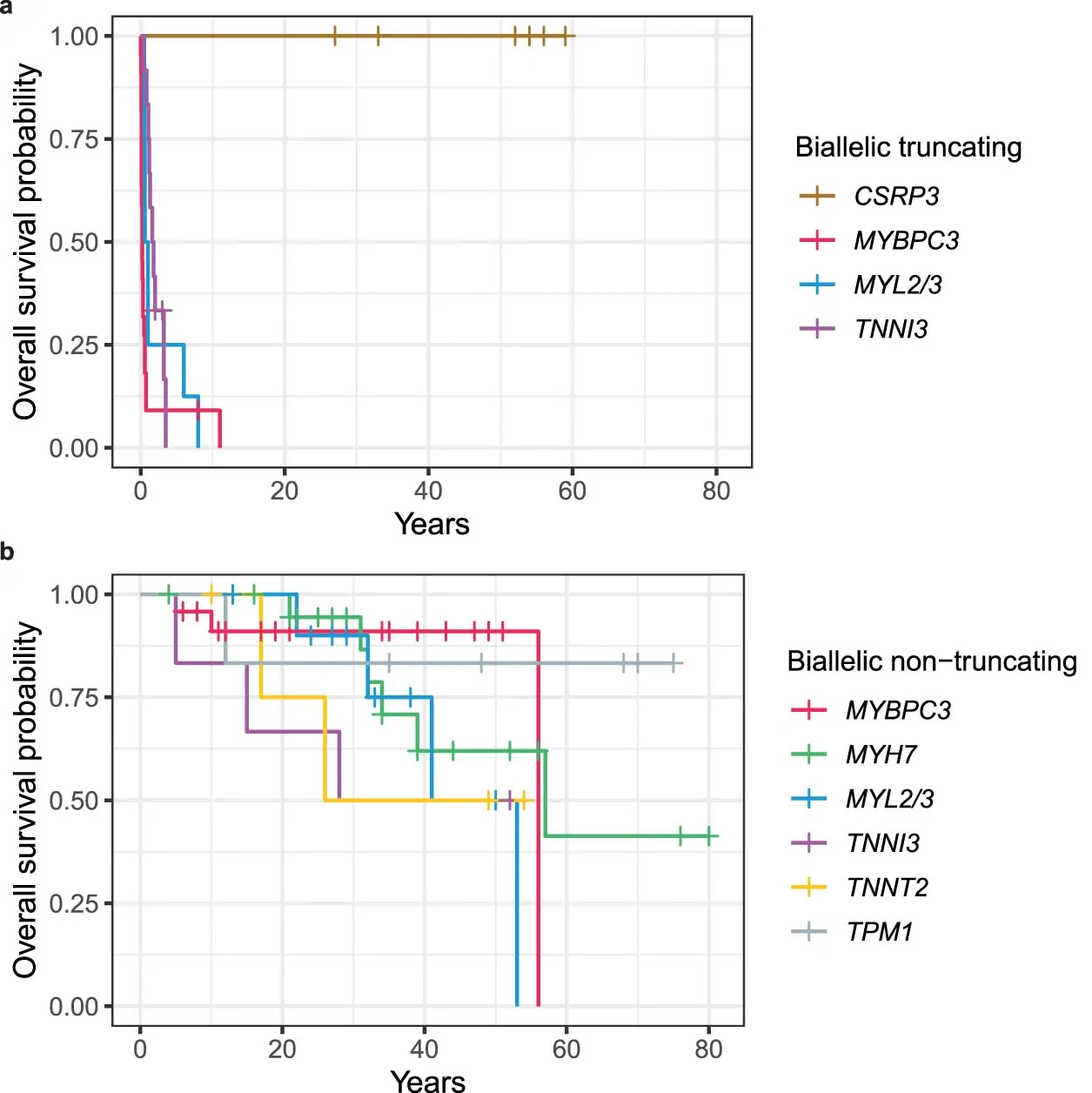
Outcome analysis for carriers of biallelic variants in genes primarily associated with autosomal dominant cardiomyopathy. Kaplan-Meier survival curves for patients with biallelic variants in autosomal dominant HCM/DCM genes (n = 121 where the outcome data is available), with survival defined as freedom from death or heart transplantation - **a**) biallelic protein-truncating variants, **b**) biallelic non-truncating variants.

Biallelic PTVs in *MYBPC3*, *TNNI3*, *MYL2* and *MYL3* are associated with severe, early-onset CM phenotypes, which can include additional features such as atrial and ventricular septal defects and skeletal myopathy (Fig. 4a,b, Extended Data Fig. 3a and Supplementary Table 5). Of these genes, only PTVs in *MYBPC3* are established as disease-causing (for HCM) in the heterozygous state; accordingly, all heterozygous relatives for PTVs in *TNNI3*, *MYL2* and *MYL3* were reported to be unaffected (Fig. 4b and Supplementary Table 5). No biallelic PTVs were reported for *MYH7*, *TNNT2* or *TPM1*, indicating that complete knockout of these genes is incompatible with life, an observation supported by the fact that mouse knockouts for *Myh6* (the functionally equivalent gene of *MYH7* in mice)^20^, *Tnnt2* (ref. ^21^) and *Tpm1* (ref. ^22^) all display embryonic lethality. Conversely, biallelic PTVs in *CSRP3* are seemingly associated with HCM in adults (based on six reported cases), with ages at onset not dissimilar to standard autosomal dominant HCM, even though most reported pathogenic variants in this gene are associated with dominant inheritance (for both missense variants and PTVs)^4,23^.

In contrast to the full knockout genotypes, biallelic non-truncating (largely missense) variants in these genes are characterized by later age at onset and more favorable outcomes (Fig. 4a and Extended Data Fig. 3b). Patients are generally diagnosed in their teens or early adulthood, although still significantly younger overall compared to patients with HCM with heterozygous variants in sarcomeric genes: median (IQR) 25.0 (13.0–35.0) years versus 37.5 (23.6–49.8) years in the SHaRe registry (*P* = 1 × 10^−10^, two-tailed *z*-test)^15^. The later age of onset observed for *TPM1* reflects the fact that four of six biallelic cases had the same Spanish founder variant (p.Arg21Leu), which is associated with a relatively mild and late-onset disease course^24^. Although the other genes display a broadly similar age at onset and outcome profiles, they can be distinguished by other features. As with biallelic PTVs, heterozygous relatives of patients with biallelic non-truncating variants in *TNNI3*, *MYL2* and *MYL3* were all reported to be unaffected, in contrast to those for *MYBPC3*, *MYH7*, *TNNT2* and *TPM1*. This may indicate that non-truncating variants in *TNNI3*, *MYL2* and *MYL3* are generally less penetrant than the other sarcomeric genes, a hypothesis supported by orthogonal evidence from other studies. For example, in an HCM cohort of high consanguinity from Egypt, missense variants in these genes were more likely to present in homozygosity (Fig. 4c), suggesting reduced penetrance when heterozygous^25^. Furthermore, a study assessing penetrance in sarcomeric variant carriers identified during family screening found that *TNNI3* variants were significantly less penetrant compared to *MYBPC3* variants (HR = 0.19, *P* = 0.001)^26^ (Fig. 4d) (note that limited numbers of *MYL2* and *MYL3* variant carriers were available in this study).

### Disease and phenotype associations in the UK Biobank

To further explore the potential spectrum of dominance and recessiveness associated with CM genes, we investigated the effects of rare heterozygous variants in the 18 genes robustly associated with recessive CM phenotypes using UK Biobank data. While the vast majority of heterozygous relatives of the cases with recessive CM were unaffected (Supplementary Table 1), age-related penetrance or subclinical phenotypic effects for these variants cannot be discounted. This has previously been demonstrated for *ALPK3;* biallelic LOF variants were initially described in early-onset CM cases with most monoallelic relatives unaffected. However, heterozygous PTVs were later found to be enriched in adult-onset HCM cohorts^12^, highlighting how the identification of rare recessive forms of disease can anticipate the discovery of more common dominant cases. Additionally, *NRAP* variants were enriched in DCM cases in both the biallelic and heterozygous states but with a much more modest effect size for the latter (odds ratio (OR) of 6.7 compared to 1,052), suggesting that such variants could act as non-Mendelian risk factors^19^.

**Fig. 5:**
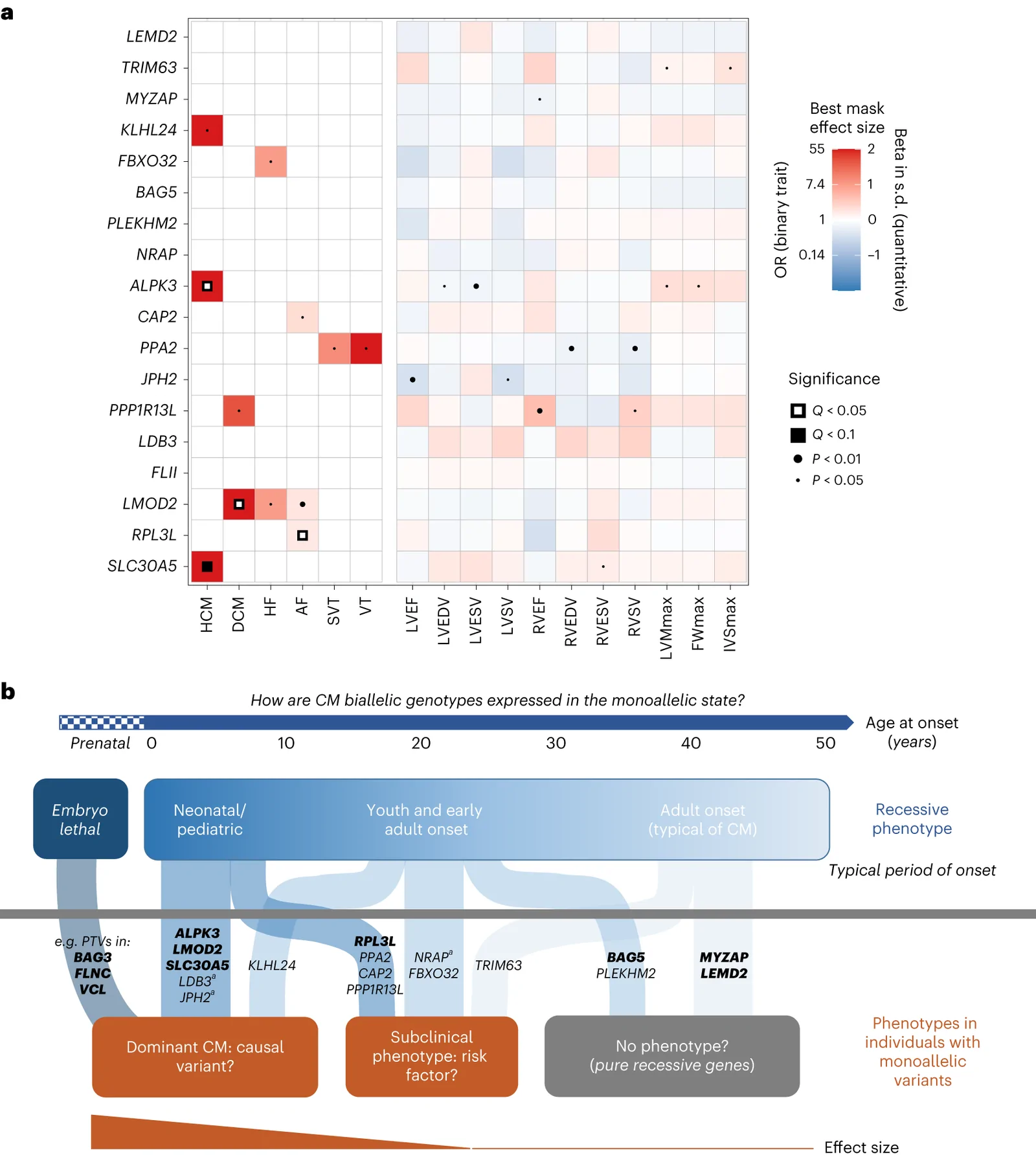
Spectrum of dominance and recessiveness in genetic CMs. **a**, Heatmap of associations between rare variants in recessive CM genes and relevant outcomes in the UK Biobank under a dominant model. Cells are colored according to the effect size of the best (lowest nominal *P* value) variant class mask for the respective gene–trait association, where red indicates increased disease risk (or higher quantitative value), and blue indicates lower disease risk (or lower quantitative value). Disease outcomes were analyzed using logistic mixed-effects models and Firth’s regression models, while the quantitative MRI traits were analyzed using linear mixed-effects models. Effect sizes for binary traits with *P* > 0.05 have been made white for clarity. *P* values represent the overall Cauchy *P* value of the gene–trait association, while *Q* values represent the Benjamini–Hochberg FDR adjusted values (see the Methods for the details). IVSmax, maximum interventricular septal mass; LVEDV, left ventricular end diastolic volume; LVEF, left ventricular ejection fraction; LVESV, left ventricular end systolic volume; LVSV, left ventricular stroke volume; RVEDV, right ventricular end diastolic volume; RVEF, right ventricular ejection fraction; RVESV, right ventricular end systolic volume; RVSV, right ventricular stroke volume. **b**, Model to summarize putative genotype–phenotype relationships for genes associated with autosomal recessive CM. Monoallelic variants in these genes can yield no detectable phenotype (that is, purely recessive inheritance), cause subclinical related phenotypic effects or act as non-Mendelian disease risk factors, or function as causal variants for adult-onset CM. Genes with more robust monoallelic associations are shown in bold. While identification of embryonic lethal biallelic variant classes is challenging given their absence in clinical records, biallelic truncating variants in genes like *BAG3*, *FLNC* and *VCL* have not been described in patients and lead to severe effects in mouse models. ^a^Note that different mechanisms were observed between recessive and dominant inheritance for *JPH2* and *LDB3*: biallelic LOF variants cause DCM, while heterozygous missense variants are associated with HCM (*JPH2* (refs. ^4,23^)) or LVNC CM (*LDB3* (refs. ^108,109^)). The monoallelic association for *NRAP* is based on the study by Koskenvuo et al. ^19^.

**Extended Data Fig. 4:**
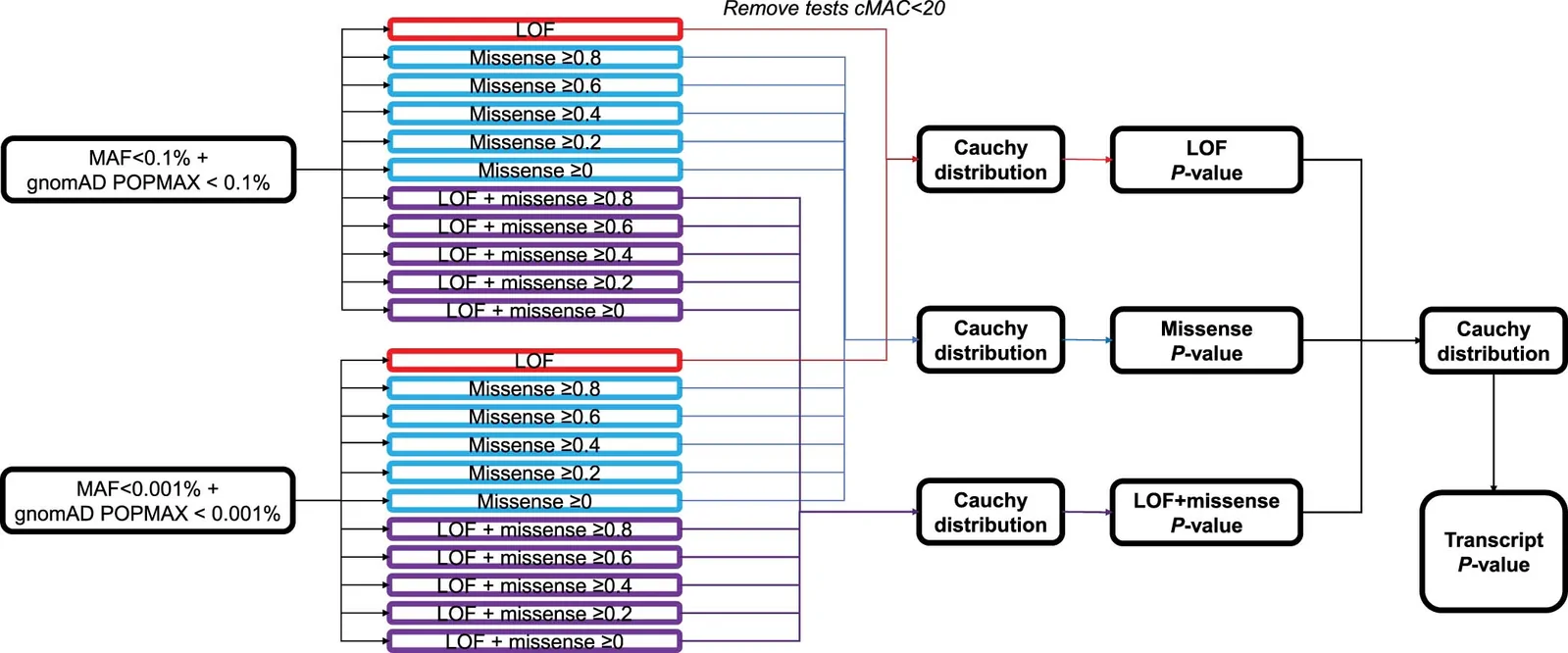
Flowchart for UK Biobank analysis of dominant associations for variants in 18 genes validated for recessive cardiomyopathy. For each gene-trait analysis, we performed rare variant collapsing tests for 22 masks based on frequency and annotation. For frequency, we applied two filters, namely minor allele frequency (MAF) < 0.1% (within the dataset and < 0.1% in each of the major continental populations in gnomAD) and MAF < 0.001% (within the dataset and each of the major continental populations in gnomAD). We analysed 11 different masks based on annotation, namely (1) loss-of-function (LOF) variants, (2) missense variants with predicted-deleteriousness score >=0.8, (3) missense variants with score >=0.6, (4) missense variants with score >=0.4, (5) missense variants with score >=0.2, (6) missense variants with score >=0, (7) LOF variants + missense with score >=0.8, (8) LOF variants + missense variants with score >=0.6, (9) LOF variants + missense variants with score >=0.4, (10) LOF variants + missense variants with score >=0.2, and (11) LOF variants + missense variants with score >=0. The 2 frequency filters times 11 annotation filters equated to 22 mask-phenotype tests per gene-phenotype pair. We removed any tests with < 20 cumulative minor allele count (cMAC) to prevent miscalibrated results from low counts. We then combined the various P-values from the LOF masks into a single LOF P-value using the Cauchy distribution test; we combined the various missense masks into a single missense P-value using the Cauchy distribution tests; and we combined the various LOF+missense masks into a single P-value using the Cauchy distribution test. Finally, these three P-values (for LOF, missense, and LOF+missense) were combined using a final Cauchy distribution test to produce a final omnibus P-value for the entire gene-trait analysis. Of note, LOF variants are referred to as ‘protein-truncating variants’ (PTVs) in the main text. See Methods for further details on annotation, missense predicted-deleteriousness score, association testing models, and the Cauchy distribution test.

The UK Biobank data comprised health records and exome sequencing data on 454,162 relatively healthy, middle-aged individuals. For the 18 recessive CM genes, we performed rare variant collapsing tests under a dominant model across six curated disease end points (HCM, DCM, HF, AF, supraventricular tachycardia (SVT) and ventricular tachycardia (VT); Supplementary Table 6 and Extended Data Table 3). For each gene, we tested 22 different rare variant masks based on variant frequency and variant annotation, and combined the various *P* values into a single omnibus *P* value (using the Cauchy distribution) for each gene–trait pair (Extended Data Fig. 4 and Methods). In a secondary analysis of up to 38,066 samples with magnetic resonance imaging (MRI) data (Extended Data Table 3), we used the same pipeline to perform rare variant collapsing tests across 11 quantitative functional, volumetric and hypertrophy traits from cardiac MRI. An overview of the results is presented in Fig. 5 and Supplementary Table 7.

As expected, a significant association between *ALPK3* and HCM was observed under a dominant model (OR = 14.3 (7.2–28.3) for rare PTVs; *P*_Cauchy_ = 2.5 × 10^−7^, false discovery rate (FDR) *Q*_Cauchy_ = 2.7 × 10^−5^; Supplementary Tables 7 and 8), which is consistent with the recent demonstration of an enrichment of heterozygous *ALPK3* PTVs in patients with HCM^12^. Despite the markedly smaller sample size for analysis of the MRI data (approximately 8% of participants with exome data), nominal associations (*P*_Cauchy_ < 0.05) with LV traits were also detected for *ALPK3*: LV end diastolic and systolic volumes and measures of hypertrophy (maximum left ventricular mass (LVMmax) and maximum free wall mass (FWmax)). Interestingly, we also observed a strong association between *LMOD2* and DCM under a dominant model (OR = 13.7 (5.4–35.2) for rare PTVs; *P*_Cauchy_ = 7.8 × 10^−4^, FDR *Q*_Cauchy_ = 0.028; Supplementary Tables 7 and 8), as well as nominal associations with HF and AF (*P*_Cauchy_ < 0.05). Like *ALPK3*, biallelic PTVs in *LMOD2* cause severe, early-onset CM and this finding suggests that *LMOD2* haploinsufficiency (caused by heterozygous PTVs) may also lead to adult-onset disease. A significant but modest association between *RPL3L* and AF was also detected (OR = 1.22 (1.11–1.34) for all missense variants; *P*_Cauchy_ = 9.1 × 10^−5^, FDR *Q*_Cauchy_ = 0.005), which is consistent with reported GWAS associations with AF for low frequency *RPL3L* missense and splice donor variants^27,28^. Notably, rare variant association studies (RVAS) also demonstrated an overlap between CM and AF genes^29,30^. Using data from the Massachusetts General Brigham Biobank (MGB), these associations were replicated for *ALPK3* with ‘other HCM’ and *LMOD2* with ‘primary/intrinsic CM’, while for *RPL3L* the association was not statistically significant but with a consistent direction of effect (Supplementary Table 9).

Several other dominant model associations were observed which, although of only nominal statistical significance, align with the recessive phenotypes described in this analysis and support the hypothesis that heterozygous variants in some of these genes may lead to later-onset disease or have hypomorphic or subclinical phenotypic effects. Biallelic LOF variants in *KLHL24* cause HCM with onset in teens and young adults; in this study, we show that heterozygous PTVs are nominally associated with HCM (OR = 19.4 (3.9–98.1) for rare PTVs; *P*_Cauchy_ = 0.049) in the UK Biobank data. Biallelic LOF variants in *TRIM63* are associated with a later and more typically adult onset of HCM; consequently, we see dominant associations (*P*_Cauchy_ < 0.05) only with cardiac MRI traits of LV hypertrophy rather than HCM itself, suggesting that *TRIM63* heterozygous variants could act as HCM risk factors. Other notable putative dominant associations include *SLC30A5* with HCM (OR = 40.6 (11.2–147.6) for rare PTVs; *P*_Cauchy_ = 2.9 × 10^−3^), a gene linked with severe neonatal recessive CM in two families; *FBXO32* with HF (OR = 2.64 (1.49–4.68) for ultrarare missense variants; *P*_Cauchy_ = 0.019), a gene where biallelic non-truncating variants have been linked with DCM with onset in teens and young adults; and *PPA2* with ventricular and supraventricular arrhythmias (*P*_Cauchy_ < 0.05), which is consistent with its recessive association with a highly arrhythmogenic phenotype that causes sudden death in young people.

## Discussion

This meta-analysis of biallelic CM genotypes highlighted a remarkable increase in robust recessive gene–disease associations in recent years, revealed that many of these associations exist on a spectrum of dominant and recessive inheritance, and demonstrated how integrated analysis of recessive genetic pedigrees and biobank datasets can enable the identification of new associations.

We identified 18 genes robustly associated with autosomal recessive inheritance for isolated or primary CM phenotypes. Although most of these associations are based on relatively limited numbers of recent studies, we can be confident in their validity because of the methods used to identify causal variants. Trio-style studies are powerful approaches to identify recessive causes of disease because a limited number of plausible homozygous or compound heterozygous candidate variants are usually inherited from unaffected heterozygous parents. The analysis of individual family pedigrees may be prone to erroneous identification of putative causal variants because of issues with variant detection (causal variants may be noncoding or otherwise not identified through exome sequencing), inadequately designed variant filtering pipelines or incorrect selection of the final candidate variant. However, the detection of biallelic variants in the same gene in multiple family pedigrees with consistent phenotypes, in conjunction with the orthogonal supporting evidence described above (high LOD scores, cohort enrichment, absence of biallelic genotypes from population databases, mouse models, GWAS associations), allows us to confidently propose these genes as being robustly associated with CM.

For the 13 other genes reported for recessive CMs (Extended Data Table 2), replication in additional studies is needed given the potential for false positive associations based on single pedigrees. Interestingly, although PTVs were the predominant variant class for 13 of 18 robustly associated genes, only one of these 13 other genes (*RHBDF1*) had a bona fide biallelic, nonsensemediated decay-competent PTV. This may indicate that some of these causal variants could have been erroneously selected given the generally lower prior probability of pathogenicity associated with missense variants. Conversely, orthogonal evidence corroborates pathogenicity for some of these candidate genes (Extended Data Table 2 and Supplementary Text). For example, the taurine transporter *SLC6A6* is supported by animal model data, putative GWAS associations for HCM^31^ and DCM^32^ and the use of taurine as a treatment for DCM in domesticated animals^33^, while the manganese superoxide dismutase *SOD2* is also supported by animal model and GWAS (fibrosis phenotype) evidence.

Research in bottleneck populations or regions characterized by high rates of consanguinity, the latter historically understudied in genomics research, greatly facilitates the identification of rare disease genes associated with recessive inheritance (Fig. 3a). Such findings are critical to increase the utility of clinical genetic testing, especially in these population groups, and enhance our understanding of the genetic architecture, pathways and pathophysiology of diseases like CMs. Importantly, their identification can also pinpoint more prevalent adult-onset dominant forms of disease associated with the same genes. This has been previously demonstrated by the initial discovery of *ALPK3* as a cause of rare early-onset recessive CM and later as a more common subtype of dominant adult-onset HCM. Likewise, in this study we identified putative dominant associations in several genes that aligned with the recessive phenotypes, for instance, for DCM (*LMOD2*) and HCM (*KLHL24*, *SLC30A5*), building on their initial descriptions in rare recessive cases, although further studies are needed to validate these associations.

The availability of vast exome and genome sequenced datasets, for both population-based biobanks and clinical cohorts, now theoretically enables the unbiased identification of autosomal dominant disease genes through RVAS. In practice, however, for relatively rare diseases like CMs only the most prevalent disease genes are likely to be detected at genome-wide significance with current sample sizes, most of which will already be established associations. Signals for less prevalent and new disease genes may exist at subgenome-wide significance thresholds. However, distinguishing these from the noise of potentially false positive hits requires either a considerable expansion of biobank sample sizes (which may not be feasible in the short term) or the judicious use of additional orthogonal lines of evidence to establish pathogenicity. The identification of recessively inherited phenotypes for such genes provides perhaps the most direct evidence for prioritizing dominant genes and is arguably even more relevant than data from mouse knockout models or GWAS loci for related phenotypes. Our findings suggest that integrated approaches combining research on rare recessively inherited disease cases (especially in understudied populations) with large-scale biobank studies can effectively detect disease genes and uncover the complex underlying genetics across many rare disease domains.

The insights into CM genetics in this study demonstrated that most genes are likely to exist on a spectrum of dominant and recessive inheritance rather than acting purely through either model. This complements recent insights from large-scale biobank studies that many variants associated with recessive disease demonstrate phenotypic effects in heterozygosity^2,3^. Understanding the particular characteristics and phenotypic associations of each disease gene and variant class will enable more effective interpretation of variants detected in individuals. Monoallelic and biallelic associations may be regarded as single semidominant conditions with differences restricted to severity or age at onset or distinct conditions where differences in phenotype or molecular mechanism are more pronounced. For genes discovered through and largely associated with recessive inheritance, this can range from a pure recessive model to a semidominant-like model where heterozygous variants can be causal factors for later-onset and less severe disease. For such genes, the severity and age at onset of the recessive phenotype appears to correlate with the likelihood, and effect size, of a monoallelic association (Fig. 5b). We also observed a spectrum of phenotypic severity and penetrance for genes largely associated with dominant inheritance, which is probably specific to variant class and even individual variants. Genes with variants that are generally associated with lower penetrance are more likely to be observed in homozygosity in patients and may therefore be more prevalent disease genes in populations of high consanguinity^25^.

The inclusion of the robustly associated CM genes described in this study in diagnostic gene panels probably has clinical utility, especially for the particular ancestries and patient groups where these genes may be more prevalent causes of disease. This will require comprehensive evaluation of the reported evidence, particularly by the ClinGen gene–disease curation initiative, which has been one of the notable efforts undertaken in recent years to define robust and clinically actionable associations for inclusion in genetic testing panels. Although most of the studies evaluated in this study were published after the initial CM ClinGen curations were initiated^4–6^, future re-curation efforts will classify these associations using its standardized scoring system and aided by the collated published evidence described in this study. The ClinGen framework evaluates both dominant and recessive inheritance models, although it arguably does not sufficiently weigh the highly informative nature of replicated trio-style approaches for identifying pathogenic variants in rare recessive disease genes, even in pedigrees of limited size, and the extreme rarity of such genotypes in the population compared to heterozygous variants. Importantly, for genes with both monoallelic and biallelic associations, these curation efforts will need to determine whether these should be evaluated together as a single semidominant condition or whether there are sufficient differences in phenotype or mechanism of disease for them to be considered as two distinct entities^34^.

In conclusion, we characterized the rapid expansion of CM genetic associations driven by studies of recessive inheritance and demonstrated gene-specific spectrums of effects across monoallelic and biallelic variants. The approaches described in this article can be applied across other diseases with similarly complex genetic architectures to characterize gene–disease relationships and identify new associations. This study highlights the need for integrated research efforts to define genetic etiology and more comprehensive clinical genetic testing of patients and at-risk individuals.

## Methods

### Literature search and triaging of abstracts

To identify publications associated with recessive CM genotypes, a PubMed search was performed with the following terms: (((cardiomyopathy OR HCM OR DCM OR ARVC OR ACM OR RCM OR NCCM OR LVNC) AND (recessive OR biallelic OR homozygous OR homozygosis OR homozygosity OR homozygote OR compound heterozygous OR compound heterozygote OR compound heterozygotes) AND ((english[Filter]) AND (1990:2023[pdat]))) NOT (‘review’[Publication Type])) NOT (‘systematic review’[Publication Type]).

The search included terms associated with CM phenotypes (and subtypes) and recessive or biallelic inheritance, and was restricted to English-language non-review articles published between 1990 and 2023.

Abstracts (and where necessary full-text articles) were then triaged to restrict the search to articles describing human clinical reports with recessive and biallelic genotypes in patients whose phenotypes included CM. Relevant studies referred to in these articles that were not detected by the original search were also included. The reports were subsequently categorized into the following four groups: (1) autosomal recessive inheritance for isolated CMs or where CM was consistently the presenting or primary clinical symptom; (2) biallelic genotypes in genes primarily associated with autosomal dominant CM based on the recent ClinGen curation efforts for HCM^4^, DCM^5^ and ARVC^6^; (3) syndromic or multisystem autosomal recessive conditions where there were reports of isolated CM phenotypes or the patients initially presented with CM, that is, genocopy conditions; (4) syndromic or multisystem autosomal recessive conditions where CM was one of many phenotypic symptoms and without reports of isolated CM presentation.

### Data mining and analysis of recessive CM gene reports

Studies reporting genes associated with CM with autosomal recessive inheritance (group 1) were assessed in detail and the following data were extracted (details in Supplementary Table 1; gene summaries in the Supplementary Text).

For each family pedigree: their country of origin; sequencing and analysis strategy to identify probable causal variants for recessive inheritance; number of affected individuals with the recessive genotype; whether the parents were heterozygotes; number of unaffected individuals with the causal variant in the heterozygous form; LOD score for the pedigree if reported.

For each reported causal variant(s): genomic description (chromosome, coordinate, reference and alternative alleles); RefSeq transcript ID and Human Genome Variation Society (HGVS) description for the coding sequence and protein; variant class (missense, inframe indel, nonsense, frameshift, splice acceptor, splice donor, splice other, start lost, stop lost); the maximum minor allele frequency (MAF) for the subpopulations in gnomAD v.2; the number of homozygotes for the variant in gnomAD v.2; variant zygosity, that is, homozygous, compound heterozygous (two variants in the same gene demonstrated as being on different alleles, that is, each parent was heterozygous for one), double heterozygous (two variants in the same gene but without proof that they were on different alleles).

For each patient with CM (where information was reported): sex assigned at birth; CM subtype, that is, ACM, DCM, HCM, LVNC, RCM or more general CM-related phenotypes: CM, HF, SCD; age at onset of symptoms; reported outcome, that is, alive (at the time of the study), deceased, heart transplant, insertion of an LVAD, fetal termination or unknown; age at the reported outcome; summary of clinical phenotype.

Additionally, any details on functional validation of the reported variants or other comments were noted, including any affected relatives where the genotype was unavailable. Individuals who were not sequenced or genotyped for the causal variants were excluded from the analysis, even when their genotype was assumed to be the same as the proband given the similarity in terms of disease severity and clinical course (such cases are noted in the ‘Comments’ column).

### Identification of robustly associated genes

Based on the analysis of all reported cases, genes were considered as replicated for association with CM with recessive inheritance if biallelic variants were identified in multiple family pedigrees using genome-wide approaches (exome or genome sequencing, potentially with runs of homozygosity analysis, followed by variant filtering, selection of putative causal variants and further segregation analysis in the family pedigree if available) or there was a demonstrated enrichment of biallelic variants in case cohorts with CM compared to controls. The exception was for genes solely reported in more than one patient or family in a large cohort study where limited information was available for each patient.

For each gene, the number of cases are summarized in Table 1 by four classes of evidence: (1) genome-wide approaches with proven recessive inheritance (that is, heterozygosity of variants in one or both parents) (‘exome trio’); (2) genome-wide approaches where recessive inheritance was not explicitly proven (that is, genotyping only in proband) (‘exome proband’); (3) panel-based sequencing of a limited number of CM-associated genes with proven recessive inheritance (‘panel trio’); and (4) panel-based sequencing where recessive inheritance was not explicitly proven (‘panel proband’). Additional evidence for pathogenicity was also highlighted, that is, high LOD scores in large family pedigrees, case-control enrichment of rare biallelic variants, concordant data from mouse models and relevant GWAS associations where the gene was the putative causal gene.

Details of the genes considered as not currently replicated are shown in Extended Data Table 2, along with additional evidence (for example, from mouse knockout data) that could support a causal role for the gene in recessive CM. Note that if any reported variants were present in multiple gnomAD individuals in the homozygous state, this was considered strong evidence against pathogenicity. Two genes that were implicated in severe early-onset, recessive CM based only on single variants with multiple homozygous gnomAD samples were excluded from the candidate genes (*GJB4* p.Glu204Ala^35^, 878 gnomAD v.2 homozygotes; *LRRC10* p.Ile195Thr^36^, 5 gnomAD v.2 homozygotes, aged 40–65).

Text summaries providing an overview of the evidence for association with CM are available in the Supplementary Text. These summarize the clinical and genetic evidence from the published reports and additionally highlight any evidence for a phenotypic effect for monoallelic variants in these genes, evidence from mouse knockout studies, data on the constraint of LOF variants from the gnomAD v.2 database (LOF observed/expected upper bound fraction metric, for genes where the reported variants were mostly or solely PTVs) and information on the gene and protein function.

### Presence of biallelic LOF variants in recessive genes in population datasets

The number of individuals with biallelic truncating variants in several population databases with direct exome or genome sequencing was assessed for the 13 genes where PTVs were the predominant pathogenic variant class to highlight the overall rarity of such variant classes. The number of individuals with high-confidence homozygous LOF variants (excluding those predicted to escape nonsense-mediated decay) was calculated for gnomAD v.2 (*n* = 125,748), gnomAD v.3 (*n* = 57,344, excluding v.2 individuals), the UK Biobank (*n* = 454,162) and datasets from ancestries with high consanguinity rates, that is, the Pakistan Risk of Myocardial Infarction Study^13^ cohort (*n* = 10,503) and British adults of Pakistani heritage^14^ (*n* = 3,222) (total number of individuals = 650,979). Additionally, we assessed individuals with two different PTVs where these were either predicted to occur in *trans* or were classified as ‘unphased’ (that is, not predicted as either in *trans* or in *cis*), based on variant co-occurrence analysis in gnomAD v.2 (https://gnomad.broadinstitute.org/news/2023-03-variant-co-occurrence-counts-by-gene-in-gnomad/). We also assessed individuals with predicted compound heterozygous LOF variants in the UK Biobank based on the phasing of 452,644 exome-sequenced samples using the SHAPEIT5 method^37^.

### Calculation of LOD scores in reported family pedigrees

LOD scores are highlighted in Table 1 and Supplementary Table 1 where these are reported. For large pedigrees where LOD scores were not provided in published reports, an estimated LOD score was calculated based on the recommendations by the ClinGen gene–disease curation process^38^. Specifically, this required a minimum of three affected biallelic cases, known genotypes for affected and unaffected individuals and clear demonstration of recessive inheritance (that is, at least one parent shown to be heterozygous). Estimated LOD scores were calculated for *LEMD2* and *PLEKHM2*.

### Outcome and age at onset analysis

Robustly associated recessive CM genes with more than eight individuals with outcome data, of which there were seven genes, were examined in an outcome analysis. Kaplan–Meier estimates of the survivor function for the individuals was calculated using the survfit2 function in the ggsurvfit v.0.2.1 package in R v.4.2.2. Survival was defined as being free from the adverse outcomes: LVAD, heart transplant or death. Individuals who were still reported free from LVAD, transplant or death were censored at the age corresponding to the time of the latest report of survival. Censor marks are shown as vertical lines on the Kaplan–Meier curves. Time zero was set as the birth of the individuals. A Cox proportional hazards model, with no covariates included other than the causal gene, was used to compare adverse outcome hazards in individuals between the seven genes using *NRAP* as the baseline. The coxph function from the package survival v.3.4.0 in R v.4.2.2. was used. A similar analysis was performed for age at onset for genes with more than eight individuals with age at onset data, with survival defined as freedom from onset of disease and *NRAP* again used as the baseline for the Cox proportional hazards regression to compare differences between genes.

### Frequency of heterozygous LOF variants in recessive genes in gnomAD populations

The percentage of individuals with LOF variants for each major population group in gnomAD was calculated for each gene robustly associated with recessive CM (where PTVs were the predominant pathogenic variant class). Data were collated from 125,748 exome samples from gnomAD v.2 and 57,344 non-gnomAD v.2 genome samples from gnomAD v.3.1. The total number of samples per ancestry group were: 22,505 African; 6,578 Ashkenazi Jewish; 10,611 East Asian; 14,486 Finnish; 24,174 Latino; 82,873 non-Finnish European; and 17,254 South Asian. Amish (455) and Middle Eastern (154) samples were excluded because of small sample sizes, as were samples classified as ‘Other’ (4,002), which did not unambiguously cluster with the major populations. LOF variants are defined as PTVs affecting the default transcripts for each gene (nonsense, frameshift, splice acceptor or donor variants) and missense variants that have been recurrently implicated as causal variants in published studies (that is, *NRAP* p.Gln24His, *TRIM63* p.Cys23Tyr, *TRIM63* p.Cys75Tyr, *PPP1R13L* p.Trp799Ser). LOF variants flagged as low quality in gnomAD, and those predicted to escape nonsense-mediated decay, were excluded.

### Biallelic variant reports in CM genes with predominantly autosomal dominant inheritance

We assessed reports with biallelic variants in genes primarily associated with autosomal dominant CM. Analysis was focused on sarcomeric genes associated with HCM (a subset of the genes are also associated with DCM) with multiple reports of biallelic variants, that is, *MYBPC3*, *MYH7*, *TNNT2*, *TPM1*, *TNNI3*, *MYL2* and *MYL3*, as well as *CSRP3*. For each report, the following data were obtained: sex assigned at birth; CM subtype and other phenotypic features, if described; age at onset of symptoms; reported outcome, that is, alive (at the time of the study), deceased or heart transplant; age at the reported outcome; variant details, that is, HGVS description for the coding sequence and protein, variant class (biallelic truncating, biallelic non-truncating or biallelic mixed) and zygosity (homozygous or compound heterozygous); clinical status of heterozygous relatives.

### UK Biobank analysis

The UK Biobank is an ongoing prospective cohort study that includes over half a million middle-aged participants (40–69 at enrollment) from the UK^39^. The biobank hosts rich phenotypic data, including anthropometric measurements, serum biomarkers, health surveys, death registry linkage, electronic health record linkage and whole-body MRI, as well as genetic data including exome sequencing for 454,787 samples^39,40^. The UK Biobank resource was approved by the UK Biobank Research Ethics Committee and all participants provided written informed consent to participate. Use of UK Biobank data was performed under application no. 17488 and was approved by the local Massachusetts General Hospital institutional review board.

Exome sequencing was performed on 454,787 individuals from the UK Biobank as described previously^40,41^. We used the OQFE exome call set and closely followed a previously published pipeline to perform stringent quality control of the exome sequencing data^42^. Briefly, we removed low-quality genotype calls (based on read depth, allele balance and genotype quality) and then removed variants based on call rate (<90%), Hardy–Weinberg equilibrium (*P* < 1 × 10^−15^), presence in low-complexity regions and minor allele count (=0). Sample quality control consisted of removal of samples with revoked consent, duplicates, samples with a mismatch between genetically inferred and self-reported sex, samples with low call rates (<90%) and samples who were outliers for several other metrics (transition:transversion ratio, single nucleotide variant:indel ratio, heterozygous:homozygous ratio, number of singletons). For these metrics, we removed individuals found to be outside of eight standard deviations from the mean, after regressing out the first 20 principal components of ancestry. After quality control, we were left with 18,752,405 high-quality autosomal variants and 454,210 high-quality samples.

The protein consequences of variants were explored using dbNSFP (v.4.3)^43^ and the Loss-Of-Function Transcript Effect Estimator (LOFTEE)^44^ implemented in the Ensembl Variant Effect Predictor (v.105)^45^ (https://github.com/konradjk/loftee). We focused on variants affecting the Ensembl canonical transcripts of protein-coding genes, which were annotated as either missense or high-confidence LOF by LOFTEE. LOF variants flagged by LOFTEE as dubious were removed. In this article, we refer to these high-confidence LOF variants as PTVs. Missense variants were assigned a missense score representing the proportion of bioinformatics tools predicting a damaging effect, according to previously published methods^42^. Briefly, we used information from 31 tools included in the dbNSFP to score each missense variant by the number of tools predicting a damaging or deleterious effect, and divided this value by the number of tools that gave a prediction. Missense variants with fewer than seven predictions were removed. For instance, if 14 tools predicted a damaging effect and 28 total tools gave a prediction, then the missense score would be 0.5 (14 of 28). Details on the scoring method and included tools have been described in detail previously^42^. We further annotated variants with continental population frequencies from gnomAD (exomes v.2)^44^ to identify the highest population frequency (‘gnomAD POPMAX’) among European, South Asian, East Asian, Admixed American and African continental populations.

In the present study, we focused our primary analyses on six curated cardiac disease end points of relevance to CM, defined using self-report, death registry data and electronic health records (Supplementary Table 6). The assessed curated disease end points were DCM, HCM, HF, AF or flutter, SVT and VT. Of the 454,210 samples with high-quality exome sequencing data, 454,162 could be linked to their electronic health record data and were used for the disease association analyses. Prevalent and incident events were combined. Baseline characteristics and disease case numbers are presented in Extended Data Table 3. We also assessed quantitative endophenotypes of cardiac function and structure, as previously defined from cardiac MRI using machine learning algorithms^46,47^. These traits include LV functional and volumetric traits (LVEF, LVEDV, LVESV, LVSV), RV functional and volumetric traits (RVEF, RVEDV, RVESV, RVSV) and cardiac hypertrophy traits (FWmax, IVSmax, LVMmax). Given the much lower available sample size for these analyses (*n* = 34,893–38,066), these traits were considered as supportive in secondary analyses.

We then performed rare variant collapsing tests to identify associations between rare variants in the 18 robustly associated recessive CM genes and the curated disease end points (and quantitative MRI traits). To this end, we used logistic mixed-effects models (or linear mixed models for the quantitative traits) implemented in custom software (https://github.com/seanjosephjurgens/UKBB_200KWES_CVD/tree/v1.2), which is a previously described adaptation^42^ of the R package GENESIS (v.2.18)^48^. We included as fixed effects: age; age^2^; sex; sequencing tranche; ancestral principal components 1–4; and any principal components from 5 to 20 if associated with the given disease at *P* < 0.05. We included a sparse kinship matrix as a random effect to account for sample relatedness^42^. Collapsing tests were run using two-sided score tests, with *P* values computed using saddlepoint approximation to account for case-control imbalance^49^. For each gene–trait end point, we ran collapsing testing for 22 potentially correlated masks constructed using two different frequency filters (MAF < 0.1%, MAF < 0.001%) and 11 different filters for inclusion of PTV and missense variants using various cutoffs for missense deleteriousness scores (Extended Data Fig. 4). Results for tests with a cumulative minor allele count of less than 20 were removed to mitigate false positive signals driven by low allele counts. Because effect sizes from logistic mixed models may be inaccurate for imbalanced data (for example, rare outcome such as HCM and rare variants), we used Firth’s bias-reduced logistic regression to reestimate effect sizes for all mask–disease associations that reached nominal *P* < 0.05 and OR > 1 in the logistic mixed model^50^.

We then aimed to produce a single association metric that combined the information from all assessed masks for a given gene–trait pair. To this end, we used the Cauchy distribution test to combine the various *P* values from each mask–trait association into a single *P* value for the gene–trait pair (Extended Data Fig. 4). The Cauchy distribution test allows for valid aggregation of multiple, potentially correlated, test statistics into a single omnibus test statistic^51^. A Benjamini–Hochberg FDR correction was applied, accounting for all diseases and all 18 genes from the primary traits, to compute *Q* values from the nominal Cauchy *P* values. A separate FDR correction was applied for the cardiac MRI traits, again accounting for all traits and all 18 genes, to compute *Q* values from the nominal Cauchy *P* values. A summary flowchart of this analysis pipeline is shown in Extended Data Fig. 4.

### Replication with the MGB Biobank

The MGB is a health system biobank from eastern Massachusetts with extensive exome sequencing data. An RVAS using various rare variant masks and testing 601 phecodes was previously performed, with results available on the following online portal: https://hugeampkpn.org/research.html?pageid=600_diseases_home. To replicate the results for our significant genes of interest, we extracted data for the following relevant phecodes: 425.12 (other_hypertrophic_cardiomyopathy; 579 cases, 51,236 controls) for *ALPK3*; 425.10 (primary/intrinsic_cardiomyopathy; 4,785 cases, 47,030 controls) for *LMOD2;* and 427.20 (atrial_fibrillation_or_flutter; 8,018 cases, 43,797 controls) for *RPL3L*. We tested for replication using the rare variant mask that most closely resembled the strongest mask in our discovery analysis, as well as for the phecode most strongly resembling the associated phenotype from our discovery analysis.

### Statistics and reproducibility

Because of the nature of the study design (analysis of published biallelic cases and cohort analysis of UK Biobank data), no statistical method was used to predetermine sample size. After selection of robustly associated genes (see above), no data were excluded from the analyses. The experiments were not randomized and the investigators were not blinded to allocation during the experiments and outcome assessment.

### Reporting summary

Further information on research design is available in the Nature Portfolio Reporting Summary linked to this article.

## Supplementary information

Supplementary Information Gene text summaries for recessive CM genes.

Reporting Summary

Supplementary Tables 1–9.

## Data Availability

All data from the published reports on biallelic genotypes in CM cases are available in Supplementary Tables 1 (genes associated with recessive inheritance) and 5 (genes primarily associated with dominant inheritance). The raw association data from the UK Biobank analysis is available in Supplementary Table 8.

## Extended data

**Extended Data Table 1:** Clinical characteristics of 241 individuals with autosomal recessive CM caused by biallelic variants in 18 validated genes

		All (n=241)	DCM (n=122)	HCM (n=53)
**Cardiomyopathy**	DCM	122 (50.6%)	–	–
	HCM	53 (22.0%)	–	–
	Other	66 (27.4%)	–	–
**Sex assigned at birth**	Female	109 (45.2%)	52 (42.6%)	21 (39.6%)
	Male	128 (53.1%)	66 (54.1%)	32 (60.4%)
	Unknown	4 (1.7%)	4 (3.3%)	0 (0%)
**Age of onset (years)**	Mean (SD)	12.2 (14.9)	8.51 (11.4)	25.4 (16.4)
	Median [Min, Max]	4.00 [0, 69.0]	3.00 [0, 56.0]	24.0 [0, 69.0]
	Unknown	22 (9.1%)	15 (12.3%)	3 (5.7%)
**Age of outcome (years)**	Mean (SD)	17.7 (19.0)	13.9 (15.8)	37.9 (17.7)
	Median [Min, Max]	11.5 [0, 78.0]	7.00 [0, 59.0]	37.5 [0.33, 78.0]
	Unknown	29 (12.0%)	13 (10.7%)	5 (9.4%)
**Outcomes**	Alive	98 (40.7%)	37 (30.3%)	42 (79.2%)
	Deceased	100 (41.5%)	54 (44.3%)	4 (7.5%)
	Heart transplant	26 (10.8%)	20 (16.4%)	3 (5.7%)
	LVAD	6 (2.5%)	6 (4.9%)	0 (0%)
	Unknown	11 (4.6%)	5 (4.1%)	4 (7.5%)
**Variant zygosity**	Compound heterozygous	64 (26.6%)	32 (26.2%)	6 (11.3%)
	Double heterozygous	12 (5.0%)	5 (4.1%)	7 (13.2%)
	Homozygous	165 (68.5%)	85 (69.7%)	40 (75.5%)
**Variant class**	Biallelic truncating	112 (46.5%)	74 (60.7%)	24 (45.3%)
	Biallelic non-truncating	104 (43.2%)	35 (28.7%)	21 (39.6%)
	Biallelic mixed	25 (10.4%)	13 (10.7%)	8 (15.1%)

DCM, dilated cardiomyopathy; HCM, hypertrophic cardiomyopathy; LVAD. Left ventricular assist device; SD, standard deviation.

**Extended Data Table 2:** Summary of genes associated with recessive CM without replication in multiple families/studies

Gene	Gene function	CM	PMID	Cases	Variant(s)	Methods
*AASDH*	Synthesises peptides independently of ribosomes	DCM	32870709	1	p.Tyr1061Cysfs*3 (last exon)	ES/HM
*ACACB*	Fatty acid synthesis enzyme	LVNC	32870709	1	p.Arg2102Gln	ES/HM
*Supporting evidence: KO mice had normal lifespan, higher fatty acid oxidation rate, lower fat (PMID:11283375)*.						
*BICD2*	Cargo adaptor protein for retrograde COPI-independent Golgi-ER transport	DCM	36068540	3	p.Arg810His	ES
*Supporting evidence: KO zebrafish displayed a greater rate of embryonic lethality; echocardiography showed a reduction in cardiac output but no significant change in cardiac area or volume, unlike DCM. RNAseq of the zebrafish showed an altered transcriptome, relative to wild-type, though the findings were not particularly specific to DCM (PMID:36068540)*.						
*CASZ1*	Zinc finger transcription factor	DCM/LVNC	32870709	1	p.Ser237Cys	ES/HM
*Supporting evidence: KO mice: embryonic lethality, abnormal heart development/shape, Z line formation (PMID:25190801). GWAS loci - atrial fibrillation (PMID:35872910,30061737,29892015), ECG traits (PMID:36050321,32916098)*.						
*GATAD1*	H3K4me3 histone code reader protein	DCM	21965549	3	p.Ser102Pro	Linkage /HM/ES
*Supporting evidence: In KO zebrafish, 1/6 had enlarged heart, all had lower survival rates (PMID:28955713)*.						
*GET3 (ASNA1)*	Chaperone for the insertion of tail-anchored proteins into the ER membrane	DCM	31461301	2	p.Cys289Trp / p.Gln305* / p.Val163Ala	ES
*Supporting evidence: Homozygous KO zebrafish died by 9 days post fertilisation. Injections of wild-type GET3/ASNA1 mRNA slowed the progression of cardiac failure whereas mutant GET3/ASNA1 mRNA did not (PMID:31461301)*.						
*KIF20A*	Kinesin motor protein for Aurora B and Golgi vesicle trafficking	RCM	29357359	2	p.Arg182Trp / p.Ser635Thrfs*15	ES
*Supporting evidence: 90% of KIF20A morpholino-knockdown zebrafish developed cardiac oedema, pooling of red blood cells proximal to the atrium, tachycardia and increased fractional shortening by 6 days post fertilisation. Wild-type KIF20A cDNA partially rescued the phenotype, whereas mutant cDNA did not. Histology of the fish showed increased ventricular thickness in the mutants (PMID:29357359). KIF20A homozygous KO mice die by 3-4 weeks of age (PMID:27626380)*.						
*PHACTR2*	Phosphatase and actin regulator	DCM/LVNC	36674904	1	p.Arg511His	ES
*RHBDF1*	Inactive intramembrane rhomboid protein that regulates EGFR signalling	DCM	32870709	3	p.Gly665Trp / p.Phe405Serfs*16	ES/HM
*SLC6A6*	Taurine transporter	DCM	31903486	2	p.Gly399Val	ES/HM
*Supporting evidence: KO mice had cardiac dysfunction and fractional shortening at older age (PMID:20804595). Putative causal gene at a DCM GWAS locus (PMID:33677556)*.						
*SOD2*	Manganese-superoxide dismutase	DCM	31494578	1	p.Gly181Val	ES
*Supporting evidence: KO mice can develop DCM and demonstrate early death (PMID:7493016,8790408). Putative causal gene at a myocardial fibrosis (interventricular septum and LV free wall) GWAS locus (PMID:37081215)*.						
*TAF1A*	Part of a transcription factor for ribosomal DNA	DCM	28472305	2	p.Leu84Ser / p.Gly341Arg	ES
*Supporting evidence: ClinVar: compound het p.Gly341Arg and p.Thr261Pro in Jamaican African-descent female 0-9 years with RCM (*SCV001775486.1*). A zebrafish TAF1A KO model showed a heart failure like phenotype; early lethality (death by 11 days post fertilisation); pericardial oedema; reduction in ventricular fractional shortening (PMID:28472305)*.						
*ULK1*	Autophagosome formation	DCM	32870709	1	p.Arg691Trp	ES/HM
*Supporting evidence: Double ULK1/ULK2 KO perinatal mice develop cardiomyopathy (PMID:34724805,35018428,35104184)*.						

DCM, dilated cardiomyopathy; ECG, electrocardiogram; ES, exome sequencing; GWAS, genome-wide association study; HM, homozygosity mapping; Linkage, linkage analysis; KO, knockout; LVNC, left ventricular non-compaction; RCM, restrictive cardiomyopathy.

**Extended Data Table 3:** Characteristics of studied participants and traits in the UK Biobank exome cohort

**Participants, n**	**454162**		
Female, n (%)	246303 (54.23)		
European ancestry, n (%)	428872 (94.4)		
Age at enrolment, mean (SD)	57.04 (8.09)		
Age at last follow-up, mean (SD)	68.00 (8.06)		
**Disease endpoint**	**Cases, n (%)**	**Controls, n (%)**	
Dilated cardiomyopathy (DCM)	924 (0.21)	438722 (99.79)	
Hypertrophic cardiomyopathy (HCM)	538 (0.12)	453624 (99.88)	
Atrial fibrillation or flutter (AF)	29149 (6.42)	425013 (93.58)	
Supraventricular tachycardia (SVT)	4836 (1.06)	449326 (98.94)	
Ventricular tachycardia (VT)	2297 (0.51)	451865 (99.49)	
Heart failure (HF)	13791 (3.04)	440268 (96.96)	
**Quantitative MRI endophenotype**	**Samples with data, n (%)**	**Mean (SD)**	**Median [Q1; Q3]**
LVEF	38066 (8.38)	0.602 (0.061)	0.604 [0.565; 0.642]
LVEDV (BSA indexed)	36818 (8.11)	75.651 (13.487)	74.512 [66.415; 83.632]
LVESV (BSA indexed)	36818 (8.11)	30.354 (8.181)	29.441 [24.803; 34.803]
LVSV (BSA indexed)	36818 (8.11)	45.297 (8.097)	44.869 [39.889; 50.234]
RVEF	36078 (7.94)	0.594 (0.064)	0.594 [0.553; 0.635]
RVEDV (BSA indexed)	34893 (7.68)	70.08 (13.705)	68.614 [60.409; 78.295]
RVESV (BSA indexed)	34893 (7.68)	28.672 (7.954)	27.723 [23.012; 33.445]
RVSV (BSA indexed)	34893 (7.68)	41.408 (8.381)	40.705 [35.7; 46.262]
LVMmax (BSA indexed)	37044 (8.16)	54.849 (10.124)	53.728 [47.467; 61.114]
FWmax (BSA indexed)	36811 (8.11)	39.586 (7.484)	38.808 [34.184; 44.148]
IVSmax (BSA indexed)	37075 (8.16)	16.68 (3.255)	16.318 [14.327; 18.669]

Baseline characteristics are provided for the entire cohort, totalling 454,162 individuals. Disease case and control counts are provided for the studied diseases; diseases were analysed in the entire cohort. We also studied certain MRI phenotypes in samples where data were available (~8%), with distributions provided as mean (SD) and median (quartiles). When logical, MRI traits were indexed to BSA. Of note, all quantitative traits were inverse-rank-normalized before genetic analyses.

BSA, body surface area; FWmax, maximum free wall mass; IVSmax, maximum interventricular septal mass; LVEDV, left ventricular end diastolic volume; LVEF, left ventricular ejection fraction; LVESV, left ventricular end systolic volume; LVMmax, left ventricular mass; LVSV, left ventricular stroke volume; MRI, magnetic resonance imaging; RVEDV, right ventricular end diastolic volume; RVEF, right ventricular ejection fraction; RVESV, right ventricular end systolic volume; RVSV, right ventricular stroke volume; SD, standard deviation.

## Code availability

The code used for the UK Biobank rare variant association analysis is available at https://github.com/seanjosephjurgens/UKBB_200KWES_CVD/tree/v1.2.
