# Supplementary material for: Exploring the complex spectrum of dominance and recessiveness in genetic cardiomyopathies
Source: Nat Cardiovasc Res. 2023 Oct 9;2(11):1078–94. doi: 10.1038/s44161-023-00346-3 (PMC11041721; doi:10.1038/s44161-023-00346-3)
Supplement: Supplementary file 1 — Supplementary Information Gene text summaries for recessive CM genes. [file 44161_2023_346_MOESM1_ESM.pdf]

nature cardiovascular research

Analysis

https://doi.org/10.1038/s44161-023-00346-3

Exploring the complex spectrum of dominance and recessiveness in genetic cardiomyopathies

In the format provided by the authors and unedited

SUPPLEMENTARY TEXT

Gene summaries for recessive cardiomyopathy genes

Below are summaries of the evidence for the association of genes with recessive forms of cardiomyopathies. For full details of the studies, genetic variants and clinical and phenotypic details of the cardiomyopathy patients, see Supplementary Table 1. The genes here are divided into those replicated in multiple family pedigrees (and therefore considered validated) and those found in single pedigrees (and therefore considered as still being candidate recessive cardiomyopathy genes).

Validated recessive cardiomyopathy genes

ALPK3

ALPK3, encoding for α-protein kinase 3, has been associated with a recessive form of cardiomyopathy in 26 individuals from 17 families across 9 publications[1–9]. The phenotypes described are variable but are often associated with a diagnosis of DCM at birth or infancy which can progress to LV hypertrophy and HCM over a period of months. There is also widespread variability in severity and outcomes for these cases - while there have been some reports of early death and heart transplantation, most patients were alive at the time of reporting and there have been several reports of later onset disease. Additional extra-cardiac, particularly dysmorphic, features are often observed in cases but there does not seem to be a consistent syndromic pattern to these. In these early reports, most heterozygous relatives of patients with biallelic truncating *ALPK3* variants were reported to be healthy, although a minority (5/37, 13.5%[6]) were diagnosed with HCM in adulthood. Subsequently a large cohort study found an association for heterozygous *ALPK3* truncating variants with adult-onset HCM, with such variants being detected in 1.6% of HCM cases across two cohorts and characterised by age-related penetrance in families (perhaps explaining the large proportion of apparently unaffected heterozygous relatives in earlier studies who are likely to be relatively young adult relatives of paediatric cases)[10]. While most of the earlier reported cases involved biallelic truncating variants, recent studies have also included missense variants (either as homozygotes or compound heterozygotes with truncating variants). As we now know that heterozygous truncating variants are associated with adult-onset HCM, the role of some missense variants (when compound heterozygous with truncating variants) may be questionable, especially for later onset disease. The *ALPK3* transcript has recently been updated (from a 1907 amino acid coding transcript to a 1705 residue one) as the previous exon 1 is now believed to be non-coding (it contains many common truncating variants in gnomAD). Because of this, three compound heterozygous cases (from two families) described by Herkert *et al*. were not included in this analysis, as one of the reported variants affected the previous exon 1[6]. Note that there may also therefore be a discrepancy with the variant descriptions of earlier reports. *ALPK3* was classified as having *Strong* evidence for association with "Infant-onset HCM/DCM" by the ClinGen HCM curation in 2018 (based on the initial reports)[11] and was upgraded to *Definitive* in February 2022. *ALPK3* is also a strong candidate to be the causal gene at a locus that has been associated with HCM, DCM and several related LV traits in multiple GWAS[12–14]. *ALPK3* demonstrates only marginal constraint of loss-of-function variants in gnomAD (LOEUF=0.91). Homozygous *ALPK3* null mice generally recapitulate phenotypes of biallelic patients, displaying myocardial hypertrophy and disarray, as well as DCM-associated features like reduced cardiac output and ejection fraction[15].

Heterozygous *ALPK3* null mice had normal contractile function and survival but developed hypertrophy after one year[16]. In summary, there is an abundance of evidence for the association of *ALPK3* truncating variants with cardiomyopathy for both recessive and dominant inheritance.

Function of ALPK3

A recent study found that *ALPK3* is actually a pseudokinase, with its kinase domain sequence having diverged to the point of lacking any catalytic activity[16]. *ALPK3* was shown to colocalise with myomesin proteins (MYOM1, MYOM2) at the nuclear envelope and sarcomere M-band. Loss-of-function *ALPK3* variants caused these myomesin proteins to mislocalize and dysregulated several additional M-band proteins involved in sarcomere protein turnover.

BAG5

BAG5, encoding for Bcl-2–associated athanogene co-chaperone 5, has been associated with a recessive form of isolated DCM in 6 individuals from 5 families in two publications[17,18]. In the main study evaluating 4 Japanese families, *BAG5* was initially highlighted through a transcriptomics study as an upregulated gene in failing hearts, with biallelic truncating variants in *BAG5* then identified in 5/536 individuals with DCM (though this cohort apparently included both probands and affected relatives, so it is difficult to estimate the overall disease frequency)[17]. For one family, the pathogenicity of the *BAG5* homozygous variant was verified through homozygosity mapping, exome sequencing and variant filtering in two affected and two unaffected individuals. The *BAG5* patients had early onset disease (four between 11-14 years and one at 34 years) characterised by rapid progression, the development of ventricular tachycardia/fibrillation and the requirement for LVAD support. Although heterozygous relatives were unaffected, heterozygous *BAG5* truncating variants were detected in 3/26 individuals with tachycardia-induced cardiomyopathy (a reversible cardiomyopathy phenotype), suggesting such variants may act as cardiomyopathy risk factors. Knock-in mice for the recurrent *BAG5* DCM-associated variant p.Arg197* displayed ventricular dilatation, reduced systolic function and arrhythmogenicity which was ameliorated by administration of an AAV-*BAG5* wild type vector. *BAG5* may be particularly prevalent in Japan as the recurrent p.Arg197* variant has a MAF of 0.000168 for in the ToMMo database of 38,000 Japanese individuals. *BAG5* is not constrained for loss-of-function variants in gnomAD (LOEUF=1.16). A subsequent genome sequencing study on a cohort of paediatric cardiomyopathy patients from Canada identify one case with the homozygous p.Gln125* variant[18]. The demonstration of multiple affected families (including through genome-wide analysis in one family) and strong supporting evidence in knock-in mice studies support a role for this gene in cardiomyopathy.

Function of BAG5

BAG5 acts as a co-chaperone for HSC70 (for which it acts as a nucleotide exchange factor) which is responsible for maintaining proteostasis (the correct folding of proteins and refolding of misfolded proteins). *BAG5* is localised to junctional membrane complexes (JMC) which are critical for calcium handling and loss–of-function variants lead to reduced levels of JMC protein, disturbed T-tubule morphology and perturbed calcium handling[17].

CAP2

CAP2 encodes adenylyl cyclase-associated protein 2, one of the two isoforms (the other being CAP1) of the ancestral protein CAP, which is important for actin organisation regulation in a wide-range of organisms. A total of 4 papers concerning the *CAP2*-cardiomyopathy association in humans have been published (two family-based, one cohort-based and a case report). Family-based and cohort-based analyses describing single patients concern a large consanguineous Bedouin family from Israel, a trio of unspecified ethnic background (both sequenced with WES) and a Pakistani cohort[19–21]. The four affected individuals across the different studies were homozygous carriers of ultra-rare protein-truncating variants and were all affected by severe forms of early-onset DCM (LVEFs 5-34%, ages of onset 0-12 years, data not reported for the patient from the Pakistani cohort) leading to congestive heart failure and/or death or enlistment for heart transplant. The Pakistani proband had a positive family history, with two siblings who had previously died of DCM. A fourth paper that has been excluded from our analysis reports on a family with two affected children with a homozygous variant in *CAP2,* but does not provide variant details[22]. Of note, the gnomAD-based LOEUF intolerance score of *CAP2* is 0.407, with an observed/expected protein-truncating variant ratio of 0.19, indicating that truncating variants are constrained in the population. Functional validation experiments demonstrated the protein truncating effects of the variant segregating in the Bedouin family and supported a nonsense-mediated-decay disease-causing mechanism for the nonsense variant observed in the trio. Tissue analysis revealed the presence of nemaline rods in the skeletal muscles of the trio's proband. In terms of non-human evidence, four *CAP2* mouse models were published[23–26]. The majority of homozygous knockout mice developed severe DCM and malignant ventricular arrhythmias and died suddenly. However, the phenotype observed in mutant mice was not always consistent with that observed in human variant carriers, as besides their cardiac phenotype knockout mice were observed to suffer also from microphtalmia, and females tended to survive close to expected levels and with live normal life spans[24]. Furthermore, heterozygous mice have also been observed to suffer from malignant arrhythmias[25]. The most recent of these papers focuses entirely on the skeletal muscle phenotype and does not describe the animals' cardiac phenotype[26]. In summary, there is convincing evidence for the association of *CAP2* with recessive DCM with four independent families (albeit one without variant details) and equivalent data in mice.

Function of CAP2

CAP2 is a 477-residue multifunctional protein that localises to the centre of the sarcomere (M-line), in close proximity to thin filament pointed ends in the heart. *CAP2* inhibits actin incorporation into thin filaments and promotes thin filament depolymerization in a tropomyosin-dependent manner by increasing the activity of cofilin by two orders of magnitude. Different from other pointed-end proteins, *CAP2*'s function is not enhanced but inhibited by tropomyosin and it does not directly control thin filament lengths. In addition, *CAP2* plays a key role in cardiomyocyte maturation by modulating pre-sarcomeric actin assembly and regulating alpha-actin composition in mature filaments. *CAP2* interacts with filamentous actin via a helical-folded domain located in the N-terminal region, and with globular actin via a Wiskott–Aldrich-homology 2 (WH2) and a CAP-retinitis pigmentosa (CARP) domain, both located in the C-terminal region.

FBXO32

An association between *FBXO32* (F-box only protein 32) and autosomal recessive DCM was found in two studies with six affected biallelic individuals across two families, one from Saudi Arabia and the

other from Iran[27,28]. Both of these MENA region countries are associated with high consanguinity rates and indeed the parents in both families are first cousins. The associated biallelic variants were non-truncating: a missense variant, p.Gly243Arg, in the Saudi family and an inframe indel variant, p.Lys295del, in the Iranian family. All heterozygous individuals were unaffected. The LOD score in the Saudi family reached a significant value of 3.37. The variants were associated with adolescent to young adult onset DCM (median age of onset 20 years; range 14–26 years). There was significant variation in outcome - most of the individuals with DCM were relatively asymptomatic, whereas a 26 year old proband developed decompensated heart failure and underwent a heart transplant. Another affected individual was reported to experience shortness of breath only. Histology of an explanted diseased heart after heart transplantation from one of the probands showed hypertrophied cardiomyocytes, and immunohistochemistry revealed apparent reduced *FBXO32* protein expression. *FBXO32* is a putative causal gene at a locus associated with HCM using a multi-trait analysis of GWAS (MTAG) approach[29]. According to the GWAS Catalog, common variant loci mapped to *FBXO32* have also been associated with atrial fibrillation[30–33] and ECG traits[34] in a number of GWAS analyses. While there are only two genetic studies for the *FBXO32* association, replication in independent families including a significant LOD score of 3.37 in one has been demonstrated, in conjunction with additional supporting GWAS evidence.

Function of FBXO32

FBXO32 encodes the F-box only protein 32. This constitutes one of the four subunits of the SCF protein complex (Skp, Cullin, F-box containing complex); the other three subunits are Skp1, Cullin and Rbx1. The particular type of F-box protein in an SCF complex varies, and so confers substrate specificity. SCF acts as the E3 ubiquitin-ligase component in the ubiquitin-proteasome system. An E2 ubiquitin-conjugating enzyme binds to the Rbx1 subunit of SCF, inducing the transfer of ubiquitin from E2 to the target protein bound by the F-box subunit of the SCF. This marks the target protein for degradation by the proteasome. In human heart samples from DCM patients homozygous for the p.Gly243Arg variant in *FBXO32*, Al-Yacoub *et al*. showed an upregulation of the CHOP transcription factor and dysregulation of endoplasmic-reticulum stress proteins, compared to control human hearts[35]. It was therefore suggested that the defective *FBXO32* protein stimulates the unfolded protein response, a type of stress response caused by the presence of unfolded or misfolded proteins in the lumen of the endoplasmic reticulum. This response leads to increased CHOP expression which activates an apoptosis pathway, thereby putatively causing DCM and heart failure.

FLII

The Al-Hassnan *et al.* cohort study of 205 Saudi Arabian unrelated probands with paediatric cardiomyopathy reported two consanguineous families with a variant in *FLII* (*FLII* Actin Remodelling Protein), associating it with paediatric autosomal recessive dilated cardiomyopathy[36]. In 2023, Ruijmbeek *et al.* reported again on the same 2 families, providing more clinical details, and on a third (non-consanguineous) pedigree from the Netherlands[37]. All the families were sequenced with WES, which revealed homozygosity for the *FLII* p.Arg1240Cys and p.Leu674Val missense variants in the first two families, and compound heterozygosity of p.Arg1168Trp and the protein-truncating p.Gln454* variant in the Dutch pedigree. Parents were sequenced in all cases and were unaffected heterozygous carriers of a single *FLII* variant passed on to the proband. In gnomAD, p.Arg1240Cys and p.Arg1168Trp are present in heterozygous form with the rare frequencies of 0.06% in East Asians and 0.009% in Finns, respectively, while p.Leu674Val and p.Gln454* are absent. Probands (two girls and one boy) were characterized by an early onset (< 1 year) and were all reported alive at the ages of 2, 3 and 7

years. The phenotype appears consistent across these three cases, with disease presentation characterized by a markedly reduced LV function (LVEF in the 23-32% range), tricuspid and mitral regurgitation and the absence of heart rhythm abnormalities or extra-cardiac features. In all cases, the proband showed either stable disease or improved cardiac function at follow-up. Using CRISPR-Cas9, Ruijmbeek *et al.* created genome-edited zebrafish with genotypes corresponding to those observed in 2 of the 3 families (i.e. homozygosity for p.Arg1230Cys - corresponding to human p.Arg1240Cys - and compound heterozygosity for p.Arg1158Trp and p.Ser449*, mimicking the variants carried by the Dutch proband). The phenotype of both zebrafish models resembled the one observed in the families, with fish showing a normal heart rate accompanied by significantly reduced contractility and EF. Using confocal microscopy coupled with 3D rendering, authors also showed that fish homozygous for p.Arg1230Cys had less organized and more primitive cardiac trabeculae besides less densely packed and less organized myofibrils and intercalated discs compared with controls. A range of other functional validation experiments were performed by Ruijmbeek *et al* on KO fish from a previously published line with a premature stop codon in place of a leucine residue at position 111, characterized by embryonic lethality[38]. These experiments showed how KO animals manifested a very severe cardiac phenotype characterized by, for example, a markedly decreased ventricular complexity (i.e. low number of trabeculae), cell architectural changes (i.e. cardiomyocytes extrusions), irregular and poorly defined filament organization, and thinner and unbundled myofibrils. In addition, expression and immunohistochemical analyses showed how FLII dysfunction also results in the dysregulation of DCM-related signaling pathways (Notch and Hippo) during ventricular morphogenesis. Of note, embryonic lethality was also observed for KO mice[39].

Function of FLII

FLII encodes the *FLII* actin remodelling protein, otherwise known as the flightless I actin binding protein. *FLII* contains a gelsolin actin binding domain and a leucine rich repeat protein interaction domain. Using the gelsolin domain, *FLII* binds to F- and G-actin and controls actin filament assembly and disassembly, for example *FLII* has the ability to sever actin filaments[40]. The *FLII* protein is involved in early embryogenesis and structural organisation, and published data overall strongly suggest a crucial role in striated muscle function.

JPH2

JPH2, which codes for the junctophilin-2 protein, has been found to be associated with recessive paediatric DCM in 7 individuals from 7 families across 6 publications[36,41–45]. Four families were from the MENA region, known for higher consanguinity rates, and one of the families was Finnish—a known genetically isolated founder population. A mixture of biallelic truncating and non-truncating variants were associated using gene sequencing panels and WES. The recessive variants in *JPH2* seem to be associated with early-onset paediatric DCM (median age of onset 3 years; range 0.67-11 years) with poor outcomes (66% deceased). *JPH2* truncating variants are marginally constrained (LOEUF=0.78) in gnomAD. *JPH2* has been classified with moderate evidence for association with DCM via a semi-dominant mode of inheritance by the ClinGen curation[46]; semi-dominant due to one *JPH2* DCM study that claimed dominant inheritance[47]. In the studies above, individuals heterozygous for the truncating and non-truncating variants were not affected. However, heterozygous missense variants in *JPH2* are known to be associated with autosomal dominant adult-onset HCM, with the gene classified as having moderate evidence for the association by the ClinGen curation[11]. *JPH2* homozygous knockout mice exhibited embryonic lethality[48]. In summary, there is substantial genetic evidence that *JPH2* is

associated with recessive DCM, although further data will be required to fully define the genotype-phenotype relationships for DCM and HCM.

Function of JPH2

Junctophilin-2 is part of the junctophilin family of proteins, which are junctional complex proteins. Junctophilin-2 is the predominant paralog in cardiac tissue[48]. The protein stretches from its membrane occupation and recognition nexus (MORN) domain located at the t-tubular sarcolemma, to its hydrophobic C-terminus located at the sarcoplasmic reticulum (SR) membrane[49]. In doing so, junctophilin-2 connects L-type calcium channels in the t-tubule sarcolemma to the ryanodine receptors in the SR membrane, allowing for dyad formation and effective calcium-induced calcium release[49].

KLHL24

KLHL24, encoding for the ubiquitin ligase substrate receptor Kelch-like (KLHL) protein 24, has been associated with a recessive form of isolated HCM in 9 individuals from 4 families across 3 publications[36,50,51]. Hedberg-Oldfors *et al*. identified two consanguineous families with biallelic loss-of-function variants in *KLHL24* – a nonsense variant (p.Glu350*) in two siblings of Iraqi origin with HCM and a missense variant (p.Arg206His) in five affected individuals in a large pedigree of Iranian ancestry[50]. The latter was identified through linkage/homozygosity mapping and WES, yielding a LOD score of 3.6. The affected individuals in these pedigrees were diagnosed at relatively early ages (16-28 years), with three other sudden deaths of individuals in their 20s reported in these families (though with unknown genotype), suggesting *KLHL24* variants are associated with a severe HCM phenotype. Additional cases identified through WES included a HCM patient diagnosed at 11 years reported in a large case series of childhood onset cardiomyopathy in Saudi Arabia (with the nonsense variant p.Trp387*)[36] and a patient of Middle Eastern origin who had a mixed cardiomyopathy phenotype of HCM, DCM and LVNC features (with the same p.Arg306His variant described in the large Iranian pedigree)[51]. Morpholino knockdown of the *KLHL24a* homologue in zebrafish resulted in similar heart defects observed with other HCM-causing variants which could not be rescued by *KLHL24a* mRNA carrying the equivalent variants observed in HCM patients (in contrast to wild type *KLHL24a* mRNA)[50]. Truncating variants in *KLHL24* are rarely observed and somewhat constrained (LOEUF=0.65) in gnomAD, suggesting this gene is likely to be a very rare cause of HCM and may be largely restricted to populations of high consanguinity. The role of *KLHL24* in cardiomyopathies is supported by the fact that 40% of cases with *KLHL24* gain-of-function start lost variants causing epidermolysis bullosa simplex, a hereditary skin fragility disorder, were also diagnosed with DCM[52,53]. In summary, there is convincing evidence for association of *KLHL24* with recessive HCM, established by strong genetic data in multiple families (including robust segregation evidence in one with a LOD score of 3.6) and other supporting evidence.

Function of KLHL24

Several KLHL proteins act as adaptors for the recruitment of substrates to Cul3-based E3 ubiquitin ligases and therefore contribute to the turnover of structural proteins in muscle cells. An upregulation of desmin was detected in the heart and skeletal muscle of the HCM patients described by Hedberg-Oldfors *et al*., suggesting it might be a substrate of *KLHL24* in myocytes[50]. This was confirmed by a study that showed *KLHL24* start-lost gain-of-function variants conversely led to a tenfold reduction in desmin protein levels (using engineered heart tissue generated from patients with such variants)[54].

LDB3

LDB3, encoding for the PDZ-LIM domain-binding factor (also known as ZASP - Z-disc Alternatively Spliced Protein) has been associated with a recessive form of DCM in 5 individuals from 5 families in a single publication[55]. All cases were associated with a severe early onset phenotype, although one was terminated during pregnancy upon discovery of cardiac defects by ultrasound and biallelic *LDB3* variants by prenatal testing. Four of the cases were from consanguineous families, suggesting this gene is likely to be more prevalent in populations of high consanguinity. The role of *LDB3* in recessive DCM is supported by animal model data, with both knockout mice (global and cardiac-specific) and zebrafish morpholino knockdown leading to severe DCM phenotypes[56–59]. While heterozygous missense variants in *LDB3* have been implicated in DCM in a number of studies, the gene was classified as having *Limited* evidence for association by the ClinGen curation, due to a lack of robust statistical genetics and functional evidence (note, this curation occurred prior to the publication of the study above)[46]. All relatives with heterozygous truncating variants in the recessive study were unaffected, although loss-of-function variants are somewhat constrained in gnomAD (LOEUF=0.68). *LDB3* is a reported mapped gene for PR interval[60] and ECG morphology[34] according to the GWAS Catalog. Notably, there are both short and long isoforms of *LDB3* expressed - variants affecting the short isoform have been implicated in skeletal myopathies. The one patient (aborted foetus) with biallelic variants affecting the short isoform displayed irregular Z-disc formation in skeletal muscle whereas the four cases with variants affecting the long isoform only had cardiac restricted phenotypes. Although only one study has thus far associated *LDB3* with recessive DCM, the demonstration of multiple affected families and strong supporting evidence from animal models support a role for this gene in recessive cardiomyopathy.

Function of LDB3

LDB3/ZASP is involved in the formation, structural stability and function of the sarcomere Z-disc, through key interactions with proteins like actin, alpha-actinin 2, myotilin and protein kinase C.

LEMD2

An association between *LEMD2* (LEM Domain Nuclear Envelope Protein 2) and autosomal recessive arrhythmic cardiomyopathy was found in one study of two families with 11 affected biallelic individuals overall[61]. These families were from the Hutterite isolated endogamous ethnoreligious population in Canada - all of the parents of the above mentioned individuals were consanguineous. The associated biallelic variant, found using WES, was a missense variant, p.Leu13Arg, in *LEMD2*. The homozygous phenotype was described as “arrhythmic” cardiomyopathy since the patients did not meet the diagnostic criteria for arrhythmogenic cardiomyopathy, but nonetheless presented with severe ventricular arrhythmias resulting in sudden cardiac death, often as the first symptom. Left ventricular fibrosis and mild impairment of LV systolic function was also characteristic of the reported cardiomyopathy. A DCM phenotype was present in one homozygous individual. There appears to be age-dependent penetrance, with onset of sudden cardiac arrest around the third and fourth decades of life. This adult onset cardiomyopathy had a median age of onset of 31 years (range 15–46 years). The outcomes were of moderate severity (death or heart transplant in 27% of the affected individuals at median age 28 years, range 27–42 years). Of the individuals genotyped as heterozygous, all showed no cardiac abnormalities on echo- and electrocardiography. The biallelic phenotype also included juvenile cataracts. A separate, earlier study from the United States in 2016 also linked the same

homozygous *LEMD2* variant to juvenile-onset cataracts by a recessive mode of inheritance in three Hutterite families—of note, six individuals with cataracts also presented with sudden cardiac death[62]. Homozygous *LEMD2* knockout mice died *in-utero*, by E11.5[63]. The p.Leu13Arg variant from the human Hutterite studies was engineered into two independent knock-in mouse models - both studies reported a DCM-like phenotype with fibrosis and arrhythmia in homozygous mice[64,65]. Wild-type *LEMD2* delivered using AAV9 rescued the phenotype in the Caravia *et al.* study[65]. Although only a single *LEMD2* variant in a founder population has been associated with recessive cardiomyopathy, the strong segregation data in multiple pedigrees, combined with equivalent phenotypes in mouse knock-in studies, support the pathogenicity of this gene.

Function of LEMD2

LEMD2 encodes the LEM domain nuclear envelope protein 2, which localises to the inner membrane of the nuclear envelope. It is thought to be involved in DNA replication, mitosis and nuclear structure organisation[66,67]. Other genes that also encode inner nuclear membrane proteins, such as LMNA, are a known cause of DCM. The 2019 Hutterite study noted earlier showed abnormal nuclear envelope morphology and condensed peripheral heterochromatin in TEM micrographs of patient cardiac tissue and fibroblasts[61]. Assays on these fibroblasts showed decreased proliferation, increased senescence and a prolonged G1 phase compared to controls. The p.Leu13Arg mouse knock-in cardiomyocytes displayed more nuclear membrane invaginations, nuclear envelope associated heterochromatin disorganisation, DNA damage and apoptosis. The interaction between mutant LEMD2 and BAF (barrier-to-autointegration factor) was found to be disrupted; BAF is required for the nuclear envelope repair process. Therefore, impaired nuclear structure organisation, possibly via disruption of nuclear envelope rupture repair, may underpin the pathophysiology of a rare recessive arrhythmic cardiomyopathy described in the Hutterite population.

LMOD2

LMOD2 encodes leiomodin 2 (LMOD2), an actin-binding protein. Published genetic evidence of bi-allelic variant pathogenicity in humans concerns a total of 5 families from 5 studies which describe 6 neonate patients affected by an extremely early-onset and severe form of DCM[68–72]. An additional case was not sequenced for *LMOD2* although her phenotype and outcome was essentially identical to that of her variant-carrying sister and has been excluded by our analysis. Of the 6 neonates included in our work, one had a later-than-usual onset at 10 months of age (potentially triggered by a viral infection, followed by heart transplant 4 months later) but in all the others disease appeared hours/days after birth. Of these neonate patients, two received heart transplant before 1 year of age, while the other 3 died within hours/weeks. All the 6 neonates were carriers of biallelic ultra-rare *LMOD2*-truncating variants in homozygosity or compound heterozygosity, with the gene characterised by a gnomAD-derived LOEUF intolerance score of 0.86 and an observed/expected ratio for protein-truncating variants of 0.48, indicating that the gene is marginally constrained toward this type of variants. Of note, one of these alleles was carried by two distinct probands, and for all these variants no homozygous individuals are present in gnomAD. Where reported, LVEF of the described neonates was in the 14-35% range and in three cases the heart was described as almost non-contractile or immobile, though not always dilated. Four of the 6 cases also developed ventricular tachycardia. Tissue staining showed in three papers that carriers' cardiomyocytes possessed significantly shorter thin filaments vs controls[68,70,72]. Of note, 2 of the 6 reported patients were carriers of the same variant (p.Trp398*) and showed some differences in terms of age of onset (0 vs 4 months), histology (while significant vs controls in both cases, the shortening of thin filaments was not of comparable severity)

and electrical activity (ventricular tachycardia and ectopy vs ventricular tachyarrhythmia). Functional validation results also demonstrated the total absence of LMOD2 protein from the heart of a homozygous nonsense variant carrier characterised by a 5-fold decrease in contraction force[68], and the lack of full-length *LMOD2* in a carrier of an essential splice site variant[70]. Non-human evidence comprises two mouse models. A *LMOD2*-knockout mouse was characterised by abnormally short thin filaments, severe contractile dysfunction and ventricular enlargement consistent with DCM and leading to death around 3 weeks of age[73]. A knock-in mouse carrying the homologous protein-truncating variant to that detected in one of the aforementioned neonates engineered to escape nonsense-mediated decay unexpectedly had a substantially less severe phenotype, with no correlation between expression level and severity (milder phenotype observed even with the lowest expression levels)[68]. In summary, consistent evidence across multiple independent studies and families, as well as mouse knockout data, support the association of *LMOD2* with recessive DCM.

Function of LMOD2

LMOD2 functions as an important regulator of thin filament by promoting elongation of actin through polymerization at pointed ends. Leiomodin is a homolog of tropomodulin, a capping protein that has the opposite effects: while tropomodulin plays a role in capping of filament ends to terminate elongation, leiomodin acts as powerful nucleator of actin polymerization in vitro to lengthen thin filaments. *LMOD2* competes for binding with Tmod1 and displaces it, allowing for actin polymerization/thin filament elongation[74].

MYZAP

MYZAP, encoding for the myocardial zonula adherens protein, has been associated with a recessive form of isolated DCM in 8 individuals from 3 families (from Finland and Slovenia) in two publications[75,76]. Homozygous truncating variants were identified in each family through exome sequencing. Age of onset ranged from 14-41 years but the cases were associated with relatively severe outcomes, with 75% of the patients either dying or requiring heart transplantation. Heterozygous relatives of the patients were all unaffected and *MYZAP* is not constrained of loss-of-function variants in gnomAD (LOEUF=1.24). Knockdown of the *MYZAP* homologue in zebrafish caused severe cardiomyopathy[77], while knockdown in mice led to heart failure and death after transverse aortic constriction[78]. A common missense variant in *MYZAP*, p.Gln254Pro, was associated with atrial fibrillation in a study using cases/controls from Iceland, UK Biobank, Norway and USA (OR=1.38) - the variant has a MAF of 0.011 in Iceland, 0.016 in Finland but only 0.0036 in UKBB/gnomAD-NFE[79]. Although only two studies have thus far implicated *MYZAP* variants in recessive cardiomyopathy, replication in multiple families with consistent phenotypes and supporting evidence from animal models and GWAS together provide substantial evidence for association.

Function of MYZAP

MYZAP is located at the intercalated disc structure of intercellular junctions between cardiomyocytes, where it interacts with desmoplakin and zonula adherens proteins. *MYZAP* is part of the broader GRINL1A complex transcription unit which comprises genes/transcripts for *GCOM1*, *MYZAP* and *POLR2M*. GTEx exon expression (PEXT metric) indicates that the cardiac tissue expression is restricted to the exons of *MYZAP*, suggesting it is the relevant cardiac gene/transcript at this locus.

NRAP

NRAP, encoding for nebulin-related anchoring protein, has been associated with a recessive form of isolated DCM in 24 individuals from 22 families across 6 publications[36,41,80–83]. In the initial reports, biallelic truncating variants were identified by exome sequencing in small family pedigrees[41,81,83] and a large case series of childhood onset cardiomyopathy in Saudi Arabia[80]. Cases were largely early onset (though this may partially reflect publication bias towards childhood disease) with severe outcomes, although two reports of unaffected homozygous carriers in their mid-30s suggests incomplete penetrance. In the cohort of childhood onset cardiomyopathy in Saudi Arabia reported by Al-Hassnan *et al*., *NRAP* accounted for 14.3% of all DCM cases with positive results, indicating it is likely to be a prevalent disease gene in regions of high consanguinity (perhaps driven by recurrent variants present at low frequency in these populations)[36]. Koskenvuo *et al*. recently published an enrichment analysis for *NRAP*, with double variants (including at least one truncating variant) detected in 1.9% of 577 DCM cases compared to none in 25912 controls and 5150 non-DCM cardiac cases (p<0.0001, OR=1052)[82]. There are some caveats with this dataset however. Only 6/11 cases were confirmed biallelic / compound heterozygous (based on homozygosity or segregation analysis). Additionally, 6/11 cases had one truncating and one missense variant, although 4 of these carried the missense variant p.Gln24His which is strongly predicted to affect splicing. While ancestry data was not provided for these cases, this study was conducted by Blueprint Genetics in Finland – the recurrent variants described (p.Thr1458Glnfs36, p.Arg1502*, p.Tyr448*, p.Gln24His) are particularly enriched in Finnish individuals in gnomAD, indicating the relatively high prevalence of *NRAP* biallelic variants may be restricted to such populations. Koskenvuo *et al*. also highlighted an enrichment of single heterozygous *NRAP* truncating variants in DCM cases (1.9%) compared to controls (0.3%) and non-DCM cardiac cases (0.5%) (p<0.0001, OR=6.7), suggesting these variants may be risk factors of intermediate effect sizes in DCM patients[82]. There is no constraint of loss-of-function variants in gnomAD however (LOEUF=1.0). *NRAP* is a reported mapped gene for QT interval according to the GWAS Catalog[84]. Upregulation of *NRAP* has been observed in mouse models of DCM[85] and in human DCM patients[86], while cardiac-specific experimental overexpression of *NRAP* led to right ventricular cardiomyopathy in mice[87]. In summary, there is abundant evidence that biallelic truncating variants in *NRAP* are associated with isolated DCM, they lead to severe and relatively early onset disease (though not always in infancy/childhood) and are likely to be particularly prevalent in regions with bottleneck populations or high consanguinity.

Function of NRAP

NRAP is involved in myofibrillogenesis during cardiomyocyte development in the foetal heart. In the adult heart, it anchors terminal actin filaments to the membrane and plays a role in tension transmission from the sarcomere to the extracellular matrix. *NRAP* comprises a LIM domain, which interacts with alpha-actinin and talin, and 46 nebulin repeats. The latter comprises a domain of 11 simple repeats, which interacts with actin, KLHL41 and muscle LIM protein (*CSRP3*), and a C-terminal section of 5 super repeats, which interacts with filamin C and vinculin.

PLEKHM2

PLEKHM2, which codes for the pleckstrin homology and RUN domain containing M2 protein, has been associated with recessive DCM with left ventricular non-compaction (LVNC) in 5 individuals, four males and one female, across two families in two studies. In a study on a Bedouin family from the Negev, Muhammad *et al.* described four DCM individuals homozygous for a truncating frameshift variant

(p.Lys645Alafs*12) in *PLEKHM2*, identified using WES and homozygosity mapping; the 11 heterozygotes were unaffected[88]. Fibroblasts from the individuals displayed impaired autophagy flux, perinuclear distribution of lysosomes and abnormal distribution of endosomes. *PLEKHM2* cDNA transfection rescued the lysosome distribution. A case study from the United States described a 21 year old female with DCM; panel testing uncovered compound heterozygosity for two *PLEKHM2* variants—a paternally inherited frameshift variant (p.Gly919Alafs*34) and a maternally inherited synonymous splice site variant (p.Ser662Ser)[89]. An *in-silico* analysis of the synonymous splice region variant predicted loss of consensus splicing (SpliceAI donor loss score = 0.72). Her father displayed syncope with bradycardia at 42 before receiving a pacemaker; SCD in her paternal grandfather and great-grandfather was reported at 65 years and 40s respectively. Her mother and siblings were healthy. Immunostaining for *PLEKHM2* showed reduced expression in the patient versus control. The recessive variants in *PLEKHM2* seem to be associated with an approximately adolescent-onset DCM (median age of onset 16 years; range 7–21 years) with moderately poor outcomes (two deceased and one heart transplantation). *PLEKHM2* truncating variants are somewhat constrained (LOEUF=0.54) in gnomAD. *PLEKHM2* has been classified with limited evidence for association with DCM via an autosomal recessive mode of inheritance by the ClinGen curation[46], due to a lack of studies - at the time of curation only the first study was published. *PLEKHM2* is a putative causal gene for a genome-wide significant locus associated with DCM-relevant LV cardiac traits in a UK Biobank study[14]. Although only two studies have thus far implicated biallelic *PLEKHM2* variants with cardiomyopathy, their identification in independent pedigrees with consistent phenotypes supports this association.

Function of PLEKHM2

The pleckstrin homology and RUN domain containing M2 protein, encoded by *PLEKHM2*, is part of a complex which links kinesin-1, a motor protein that moves to the plus-end of microtubules, to lysosomes[90]. More specifically, the WD domains of the *PLEKHM2* protein bind to the light chains of kinesin-1, and its N-terminal RUN domain binds to Arl8-GTP which itself is bound to the lysosome. The *PLEKHM2* protein also contains a C-terminal PH domain which is free - it is speculated to have functions in motor or membrane traffic regulation. Lysosomes degrade unnecessary or dysfunctional cellular components and as such their motility is critical in facilitating effective autophagy. Therefore, impaired autophagy may be important in the pathogenesis of DCM in patients with *PLEKHM2* truncating variants.

PPA2

PPA2 encodes the inorganic phosphatase 2, a ubiquitously expressed mitochondrial protein important for the phosphate metabolism of cells. Published research concerning the association of *PPA2* variants with human cardiomyopathies comprises 7 papers reporting on 32 families (with 56 affected individuals carrying biallelic variants and 64 unaffected heterozygotes) of various ethnic backgrounds[41,44,91–95]. The largest share of these families (N=23 of 32) are described in two papers published by the same group[92,94]. The vast majority (79%) of the reported biallelic variant carriers died suddenly at a very young age (mean age of death 3.6 years), either as a result of sudden arrhythmogenic events or of sudden heart failure. The rest either had an unknown clinical phenotype/outcome or were reported as alive (following transplant or ICD implantation and/or in some cases with recurrent arrhythmogenic events or severe cardiomyopathy). One was reported as affected by syndromic disease with no cardiac symptoms, and has been excluded from our analysis. In cases in which an autopsy was performed, a viral infection and/or alcohol ingestion seemed to have acted as a trigger for the sudden deterioration of cardiac function or arrhythmogenic death. Other

features shared by many cases were emesis before death, necrotic/fibrotic areas of the myocardium and lactic acidosis. Of note, cardiomyopathy or the presence of dysmorphic ventricles were described for a minority of cases (23%). Reported extra-cardiac features ranged from none to severe multi-organ involvement. The vast majority of variants reported in affected individuals are rare missense alleles carried in homozygosity or in compound heterozygosity. All reported variants are rare, with the commonest being p.Glu172Lys, observed in 0.095% of Non-Finnish Europeans in gnomAD (but no homozygotes) and remarkably recurrent in the affected individuals reported in the literature, being carried by members of 17 different families. Of note, other recurrent variants were also observed in up to 5 distinct families. Functional validation experiments showed that specific variants (such as p.Glu172Lys) caused a substantial to near-complete inactivation of the phosphatase activity and their association with decreased levels of *PPA2* protein in the myocardium[91]. Other experiments showed the loss of phosphatase in fibroblasts to be associated with a drastic reduction of mitochondrial respiratory chain Complex IV, and *in silico* modelling predicted specific variants to disrupt stabilising interactions and create new ones in the hydrophobic core of the protein[41]. No *PPA2*-related animal models are reported in the literature to date. In summary, there is an abundance of evidence that biallelic variants in *PPA2* are associated with sudden cardiac death and an arrhythmogenic cardiomyopathy in young people, although considerable heterogeneity exists in the reported phenotypes.

Function of PPA2

The inorganic phosphatase 2 hydrolyses inorganic pyrophosphate, generated by many nucleotide-dependent reactions. This activity is essential for the correct regulation of mitochondrial membrane potential, and mitochondrial organisation and function[92]. The mechanism through which inorganic pyrophosphatase deficiency can lead to heart or global multiorgan dysfunction is not well understood. A hypothesis is that the accumulation of pyrophosphate beyond a certain threshold has an impact on the regulation of mitochondrial inner membrane potential and thus leads to chronic ADP build-up, with the most disruptive consequences observed in energy-consuming organs like heart and brain[94].

PPP1R13L

PPP1R13L codes for the protein phosphatase 1 regulatory subunit 13 like, also called inhibitor of apoptosis stimulating protein of p53 (iASPP). *PPP1R13L* is expressed mainly in skin, testes, heart and stomach. This gene has been associated with a recessive, severe, early-onset form of DCM/ACM in 6 studies[36,96–100]. These describe a total of 11 families originally from Eastern Europe (5 pedigrees) and Middle Eastern countries or ancestries (6 pedigrees), all of which were investigated with WES/WGS. In most of the 15 reported variant carriers, the disease is not described specifically as DCM or ACM, but as a “cardio-cutaneous” syndrome with a certain variability in terms of extra-cardiac symptoms and their severity. While for 20% of the biallelic *PPP1R13L* variant carriers no extra-cardiac features are reported, in the majority hair and/or skin and nail anomalies were observed in addition to cardiomyopathies, with other features reported including teeth and/or head/neck anomalies, cleft lip/palate and neurological complications or cognitive delay. Two individuals from an Israeli family were not sequenced and have been excluded from our analysis, although the phenotype and clinical history were remarkably similar to that of ascertained variant carriers from the same pedigree. Most *PPP1R13L* variants detected in these homozygous or double heterozygous variant carriers are predicted to have a protein-truncating effect with the rest being missense variants. All variants are ultra-rare in the gnomAD database with no homozygous carriers. Heterozygous variant carriers (N=23) in the described families are reported as not having a related disease phenotype. Of note, *PPP1R13L*

has a gnomAD-derived LOEUF intolerance score of 0.49 with an observed/expected ratio of predicted protein-truncating variants of 0.28, indicating that the gene is constrained for loss-of-function variants. The cardiac phenotype of the described biallelic variant carriers is overt DCM or (in 1 case only) biventricular arrhythmogenic CM progressing to reduced systolic function and heart failure. Onset was consistently during early childhood (by age 4), with the exception of a single case where disease was reported to have manifested at 8 years of age. LVEF, where provided, was in the range 9-23% and in the majority of cases outcomes were either death (in all cases before 5 years of age) or heart transplant (at 2-8 years of age). Three biallelic variant carriers (of whom the oldest was 6 at the time) were reported alive. Functional validation experiments demonstrated fibroblasts from some of the biallelic variant carriers to have lower mRNA compared with control cells and no iASPP protein (supporting nonsense-mediated decay as the disease mechanism), coupled with higher mRNA levels of specific cytokine-coding genes, suggesting a pro-inflammatory effect of the pathogenic variants. This was observed also in *PPP1R13L*-knockdown murine cardiomyocytes. As far as animal models are concerned, one of the aforementioned family-based studies[96] reports on mice with spontaneous recessive *PPP1R13L* mutations that had been previously studied[101,102]. These mice get born blind with open eyelids and develop rapidly progressing and fatal DCM. Furthermore, differentially expressed genes in these mice compared with wild-type animals were reported enriched for inflammation-related pathways, and injection of a pro-inflammatory molecule in mutant mice caused death while having no deadly effect in variant-free mice. In summary, there is abundant genetic and animal model evidence that biallelic loss-of-function variants in *PPP1R13L* are associated with severe early-onset recessive cardiomyopathy with a cardio-cutaneous phenotype.

Function of PPP1R13L

iASPP is one of the most evolutionarily conserved inhibitors of the transcriptional activity of NF-kB and p53. The inhibition of p53 is possibly exerted by preventing the association between p53/TP53 and ASPP1 or ASPP2. According to the hypothesis tested by Falik-Zaccai *et al.*, the absence of iASPP would unleash NF-kB to increase transcription of pro-inflammatory mediators and, as a consequence, would lower the heart's threshold to inflammatory response, induce prolonged inflammatory processes and eventually DCM[96].

RPL3L

Four studies have found an association between *RPL3L*, which codes for ribosomal protein L3 like, and autosomal recessive infantile-onset DCM. In total, the association was found in 9 individuals, five male and four female, from 6 families in 4 studies[36,103–105]. Affected individuals were compound heterozygous or homozygous for generally non-truncating variants, identified using WGS or WES. All were missense except for one family who were compound heterozygous for a missense and frameshift variant, though the latter was C-terminal variant predicted to escape NMD. Parents were consanguineous in two of the families (from Saudi Arabia and Colombia). All heterozygous individuals were unaffected. The recessive variants in *RPL3L* are associated with very early-onset infantile DCM (median age of onset 46 days; range 1–274 days) with very poor outcomes (death or heart transplant in all individuals at median age 91 days; range 15-486 days). Non-rare missense (p.Ala75Val) and splice donor (c.1167+1G>A) variants in *RPL3L* were associated with atrial fibrillation in a study using cases/controls from Iceland, UK Biobank, Norway and USA (OR=1.2 and 1.5 respectively)[79]. Two other missense variants (pArg4Gln, p.Arg242Trp) enriched in the Finnish population were associated with atrial fibrillation in a FinnGen GWAS[106]. *RPL3L* is also a reported mapped gene for p-wave duration according to the GWAS Catalog[107]. In summary, the identification of biallelic RPL3L variants in multiple

independent families with consistent phenotypes provides robust evidence for association with recessive DCM.

Function of RPL3L

The human eukaryotic 80S ribosome, responsible for protein synthesis, is a ribonucleoprotein complex consisting of the 60S large subunit and 40S small subunit. The 60S subunit is composed of rRNA and 46 ribosomal proteins. *RPL3* encodes one of these proteins, the ribosomal protein large 3. *RPL3L*, ribosomal protein large 3-like, is a paralog of *RPL3* which has a tissue specific high level of expression in skeletal and cardiac muscle. An *in-vitro* myoblast cell line study showed a reduction in myotube growth when *RPL3L* was expressed via an inducible promoter gene expression system[108]. It follows that pathogenic variants in *RPL3L* may therefore cause DCM via some impairment of ribosomal function - in particular, dysregulated cardiac muscle growth may be involved.

SLC30A5

One study from Germany reported an association between *SLC30A5* (Solute Carrier Family 30 Member 5) and very severe and early onset recessive cardiomyopathy[109]. Four children across two families were described, all of whom were severely affected; the parents in both families were unaffected and consanguineous. Death from cardiac symptoms occurred in all four children - of these, cardiomyopathy was detected in three. One died *in-utero*, the other three died in the first few days after birth. Other non-cardiac phenotypes were described, such as dysmorphic features, cystic hygromas, small ribs, dystrophic nails and intraventricular haemorrhage, though the cardiac phenotype seemed predominant. Exome sequencing revealed all three children in family one were homozygous for the p.Ile278Phefs*33 frameshift variant in *SLC30A5*; the parents were unaffected and heterozygous. The child in family two was homozygous for the p.His661Tyrfs*10 frameshift variant in *SLC30A5*, and the parents were heterozygous. These variants are not present in the gnomAD database. Loss-of-function variants in *SLC30A5* are constrained in gnomAD (LOEUF=0.46). One *SLC30A5* knockout mouse study showed sudden cardiac death in more than 60% of the knockout mice - the mice also displayed a non-cardiac, skeletal phenotype of reduced bone density, as well as poor growth[110]. Although there have been limited genetic studies published thus far for *SLC30A5*, the identification of biallelic truncating variants in two independent families, coupled with supporting mouse knockout data, suggests this gene is associated with a severe early-onset form of recessive cardiomyopathy.

Function of SLC30A5

SLC30A5 (Solute Carrier Family 30 Member 5), also known as ZnT-5 (Zinc Transporter 5), encodes one of a family of zinc transporters. These membrane transport proteins facilitate the efflux of cytoplasmic zinc ions out of the cell, or efflux from the cytoplasm into vesicles[111]. The paper by Lieberwirth *et al*. therefore speculates that impaired zinc homeostasis may be one rare cause of lethal perinatal autosomal recessive cardiomyopathy[109].

TRIM63

Biallelic loss-of-function variants in *TRIM63*, which encodes the E3 ubiquitin protein ligase TRIM63 (also known as muscle-specific RING finger protein 1 (MuRF1)), were associated with HCM in a large case-control study[112], detected in 15 (0.4%) of 4,867 HCM probands compared with 0% in 3,136

controls. The phenotype was largely adult-onset in the 15 probands and 4 affected relatives, ranging from teens to 60s at presentation. The patients were characterised by concentric LV hypertrophy and comparatively high rates of cardiac fibrosis, LV systolic dysfunction and arrhythmias. The *TRIM63* variants were a mix of biallelic truncating (8 probands), biallelic missense (6) and mixed (1). While a previous study from 2014 proposed a modifier role for heterozygous missense variants in *TRIM63* (and *TRIM55*) in patients with HCM[113], all 32 heterozygous relatives in this study were reported to be healthy. *TRIM63* is also not constrained of loss-of-function variants in gnomAD (LOEUF=1.11). We recently demonstrated that biallelic *TRIM63* variants are highly enriched in HCM patients from Egypt compared to population-matched controls (8/374, 2.14% vs 0/400, 0%) - the five-fold enrichment in Egyptian vs the predominantly European ancestry cases in the prior study demonstrates that *TRIM63* is likely to be a more prevalent HCM gene in populations of high consanguinity[114]. These reports built upon two earlier studies on individual patients with stable HCM and mild skeletal muscle phenotypes, both of whom has the p.Gln247* in homozygosity[115,116]. While the p.Gln247* variant has also been found in both cohort studies above, it is particularly common in Ashkenazi Jews (MAF=0.008), indicating that *TRIM63* could also be a relatively common cause of HCM in individuals of this ancestry. *TRIM63* was previously curated as having limited evidence for association with HCM by the ClinGen curation, although this was assessed before the publication of the two cohort studies described above[11]. *TRIM63* is a reported mapped gene for myocardial longitudinal strain in UK Biobank data[29], as well as for QRS complex/amplitude[117] and ECG morphology[34] according to the GWAS Catalog. Knockout mice data for *TRIM63* has revealed complex phenotype correlations. *TRIM63* knockouts alone are healthy with normal cardiac muscle. Double knockouts of *TRIM63* and *TRIM55* (MURF2) display severe cardiac hypertrophy[118]. In the single gene knockout models however, *TRIM63* knockout mice developed TAC-induced LV hypertrophy whereas TRIM55 knockout mice did not, suggesting it is *TRIM63*/MURF1 that is responsible for the hypertrophy phenotype[119]. Although all reports of biallelic *TRIM63* variants in HCM were based on panel sequencing studies rather than unbiased genome-wide approaches, the clear and significant enrichment of variants in two cohorts provides robust evidence for its role as a recessive HCM gene.

Function of TRIM63

TRIM63/MURF1 localises to the Z-disk and M-line of the sarcomere where it acts to regulate the degradation of sarcomeric proteins through ubiquitylation.

Gene summaries for recessive cardiomyopathy genes without replication

The following genes are associated with recessive cardiomyopathies in single reported family pedigrees only (or 2-3 probands in one cohort-based study). The evidence available, including supporting data from animal models, GWAS etc., is described below. Further studies are required however to establish their pathogenic role in cardiomyopathy.

AASDH

The Al-Hassnan *et al.* cohort study of 205 Saudi Arabian unrelated probands with paediatric cardiomyopathy reported one family with a variant in *AASDH* (Aminoadipate-Semialdehyde Dehydrogenase), associating it with paediatric autosomal recessive dilated cardiomyopathy[36]. WES revealed homozygosity for the *AASDH* p.Tyr1061Cysfs*3 frameshift variant (this variant occurs in the last exon of the gene); the parents were consanguineous, heterozygous and unaffected. The girl presented with DCM 5 months after birth and was reported still alive at 6 years of age. In gnomAD, the variant is very rare (maximum population frequency of 0.05% in Latinos/Admixed-Americans) with no homozygotes; the gene is only marginally constrained for loss-of-function variants (LOEUF = 0.83).

Function of AASDH

AASDH encodes the aminoadipate-semialdehyde dehydrogenase enzyme. This is a non-ribosomal peptide synthetase, these enzymes usually catalyse the biosynthesis of a particular member of the class of peptides known as non-ribosomal peptides, which are named as such since they do not require ribosomes for their synthesis.

ACACB

The Al-Hassnan *et al.* cohort study of 205 Saudi Arabian unrelated probands with paediatric cardiomyopathy reported one family with a variant in *ACACB* (Acetyl-CoA Carboxylase Beta), associating it with paediatric autosomal recessive left ventricular non-compaction cardiomyopathy[36]. WES revealed homozygosity for the *ACACB* p.Arg2102Gln missense variant; the parents were consanguineous, heterozygous and unaffected. The girl presented with LVNC 21 days after birth and died at 1 year of age. In gnomAD, the variant is very rare (maximum population frequency of 0.0063% in Africans/African-Americans) with no homozygotes. *ACACB* knockout mice had a normal lifespan with higher fatty acid oxidation rate and lower fat[120].

Function of ACACB

ACACB encodes the acetyl-CoA carboxylase beta enzyme. This catalyses the carboxylation of acetyl-CoA into malonyl-CoA, which is a key substrate in the biosynthetic pathway for fatty acid production.

BICD2

One study presented evidence for an association between homozygosity for a *BICD2* (bicaudal-D cargo adaptor protein 2) variant and dilated cardiomyopathy[121]. In the studied family from China, healthy consanguineous parents had seven children, of which, two sons and one daughter were affected with DCM. Echocardiography in the proband showed poor systolic function (LVEF 26.8%) and LV dilatation (LVESV 357ml). WES of the parents and two of the affected children identified novel homozygous

variants in five gene candidates, with the *BICD2* p.Arg810His missense variant chosen as most likely to be associated with a cardiac phenotype. *BICD2* is highly expressed in the skin and oesophageal mucosa, less so in the heart (GTEx). The unaffected parents were heterozygous for the *BICD2* variant, whereas two of the affected children were homozygous. The variant is associated with poor outcomes: one of the affected individuals died after a heart transplant, the other two are presumably alive. The p.Arg810His variant is rare, with a maximum population frequency of 0.014% in gnomAD and no homozygous carriers. Immunohistochemistry demonstrated *BICD2* expression in the human heart. *BICD2* knock-out zebrafish displayed a greater rate of embryonic lethality; echocardiography showed a reduction in cardiac output but no significant change in cardiac area or volume, unlike DCM. RNA-seq of the zebrafish showed an altered transcriptome, relative to wild-type, though the findings were not particularly specific to DCM. *BICD2* variants are also associated with autosomal dominant spinal muscular atrophy[122].

Function of BICD2

BICD2 is one of the two paralogs of the gene encoding the bicaudal-D cargo adaptor protein. *BICD2* binds to activated RAB6A, a GTPase located on the membrane surface of vesicles located on the *trans* side of the Golgi apparatus[123]. This allows recruitment of the dynein-dynactin complex to the N-terminal end of *BICD2*; dynein is a motor protein that travels towards the minus end of microtubules, and dynactin is an enhancer of dynein activity. This motor complex then transports its cargo in a retrograde manner from the Golgi apparatus to the endoplasmic reticulum[124]. The study by Luo *et al*. is therefore suggestive of dysfunction in retrograde COPI-independent Golgi-ER transport as a potential rare cause of autosomal recessive DCM.

CASZ1

The Al-Hassnan *et al.* cohort study of 205 Saudi Arabian unrelated probands with paediatric cardiomyopathy reported one family with a variant in *CASZ1* (Castor Zinc Finger 1), associating it with paediatric autosomal recessive left ventricular non-compaction and dilated cardiomyopathy[36]. WGS revealed homozygosity for the *CASZ1* p.Ser237Cys missense variant; the parents were consanguineous, heterozygous and unaffected. The girl presented with LVNC and DCM 5 months after birth and was reported still alive at 6.5 years of age. The variant is absent from gnomAD. *CASZ1* knockout mice display embryonic lethality, display abnormal heart development, heart shape and Z line formation[125]. CASZ1 is a reported mapped gene for atrial fibrillation and ECG traits according to the GWAS Catalog[31–34,126].

Function of CASZ1

CASZ1 encodes the castor zinc finger 1 protein, which is a zinc finger transcription factor. It has been associated with the regulation of gene expression during cardiac development in Xenopus and mouse models[125,127].

GATAD1

One study found an association between *GATAD1*, which codes for the GATA zinc finger domain containing 1 protein, and autosomal recessive DCM[128]. Three individuals were affected, two female and one male, in a Norwegian consanguineous family where the parents were first cousins. The two female patients had DCM whereas the male displayed idiopathic left ventricular enlargement. Linkage

analysis and homozygosity mapping identified a critical region (7q21) with LOD score of 3.1; WES after variant filtration revealed the p.Ser102Pro missense variant in *GATAD1* located within this critical region. The three affected individuals were homozygous for the variant and had late-onset DCM (median age of onset 53 years old; range 50–57 years) without poor outcomes—both females are still alive at median age 75 years old (range 74–76 years) and the male died at 73 years of age but from cancer (13 heterozygotes in the family were unaffected). Two other variants were found by WES in the affected individuals after filtering: MYLK3, which encodes a cardiac specific kinase, and SETD1A, which encodes a protein involved in epigenetic gene regulation. However, these genes were ruled out since an unaffected individual was homozygous for these variants. Immunohistochemistry using *GATAD1* antibodies showed abnormal intracellular *GATAD1* localisation compared to control healthy tissue and tissues from other DCM mutations. Histology showed globular, rather than the usual rod-like, cardiomyocyte morphology. There has also been an experimental study on *GATAD1*'s association with DCM using adult zebrafish[129]. *In-situ* hybridization showed high *GATAD1* RNA expression in the heart, but the highest in the brain. Transgenic expression of *GATAD1*-GFP showed subcellular localisation near the nucleus and actin filaments. *GATAD1* knock-out zebrafish showed significant decreases in survival rates and compromised swimming capacity, however these fish were also treated with ethanol and a high-cholesterol diet, in addition there was no change in body weight adjusted ventricular area. The human *GATAD1* gene with the S102P variant was introduced into zebrafish—there was a reduction in survival but this did not reach significance, there was no significant difference in swimming capacity and only one of the six transgenic fish displayed an enlarged heart. Based on the evidence described above, the ClinGen curation classified *GATAD1* as having limited evidence for association with DCM via an autosomal recessive mode of inheritance[46].

Function of GATAD1

GATAD1 encodes GATA zinc finger domain containing 1; this protein has been identified as a H3K4me3 histone code reader protein[130]. H3K4me3 (trimethylation of the fourth residue, a lysine, in histone 3) is a post-translational modification of the tail of a histone. *GATAD1* is ubiquitously expressed across all tissues, as seen in the GTEx Portal. Patterns of H3K4me3 marks were found to be different between healthy human LV specimens and that of DCM patients with heart failure[131]. It is suggested then, that deleterious variants in *GATAD1* may cause DCM via epigenetic dysregulation.

GET3/ASNA1

An association between biallelic *GET3* (guided entry of tail-anchored proteins factor 3, ATPase) variants and dilated cardiomyopathy was found in one study from the Netherlands[132]. This gene was previously known as *ASNA1* (arsenite-stimulated ATPase) and is referred to as such in that paper. Two females were affected in a small family with non-consanguineous parents. Dilatation of the heart and poor LV contractility were observed in both. WES and filtration to novel variants revealed that they were compound heterozygous for the *GET3* variants p.Cys289Trp and p.Gln305* on the paternal copy, and p.Val163Ala on the maternal copy. A *GET3* p.Val163Ala heterozygote was unaffected. The patients had very early onset DCM (9/14 days) with very poor outcomes (death at 12/49 days). *GET3* is constrained for loss-of-function variants (LOEUF=0.35) in gnomAD. Histology of the explanted hearts showed reduced *GET3* protein expression, disorganised intercalated discs and irregularly shaped nuclei, compared to controls. *GET3* protein with the Val163Ala variant showed reduced insertion efficiency of tail-anchored proteins into the endoplasmic reticulum, measured by the presence of glycosylated tail-anchored proteins, compared to wild-type[132]. Homozygous *GET3* knockout zebrafish died by 9 days post fertilisation. Injections of wild-type *GET3* mRNA slowed the progression of cardiac

failure whereas p.Val163Ala and p.Cys289Trp-p.Gln305* mutant *GET3* mRNA did not. It is therefore suggested that disturbances in tail-anchored protein insertion could be a cause of DCM.

Function of GET3

The *GET3* gene product is a chaperone that facilitates the insertion of tail-anchored proteins from the cytosol into the membrane of the endoplasmic reticulum.

KIF20A

One study from Belgium described an association between *KIF20A* (Kinesin Family Member 20A) and lethal paediatric restrictive cardiomyopathy[133]. Two, one male and one female, out of the three children of non-consanguineous parents developed restrictive cardiomyopathy prenatally; they developed heart failure & died at 93 and 71 days of age respectively. WES revealed the two affected children as compound heterozygous for the missense p.Arg182Trp and frameshift p.Ser635Thrfs*15 variants in *KIF20A*. The unaffected child did not possess any of these variants, the parents were each heterozygous for one of the variants. The highest frequency in gnomAD for these variants was 0.0065% for the p.Arg182Trp variant in South Asians; no homozygotes are listed in gnomAD for either variant. *KIF20A* has a LOEUF of 0.74 in gnomAD, which is somewhat constrained but not as much as other genes linked to recessive cardiomyopathy. Lower abundances of *KIF20A* transcripts and protein were measured in the fibroblasts of the RCM patients. Immunostaining of these cells revealed an abnormal subcellular localisation of KIF20A, and subsequent failure of the Aurora B protein (trafficked by KIF20A) to localise at the spindle midzone during mitosis. ATPase assays showed that the mutant KIF20A motor protein had an almost complete loss of function compared to wild-type. Zebrafish were subjected to *KIF20A* knockdown using Morpholinos; 90% of these fish developed cardiac oedema, pooling of red blood cells proximal to the atrium, tachycardia and increased fractional shortening by 6 days post fertilisation. Wild-type *KIF20A* cDNA partially rescued the phenotype, whereas mutant cDNA did not. Histology of the fish showed increased ventricular thickness in the mutants. In a different study, *KIF20A* homozygous knockout mice die by 3-4 weeks of age[134]. The authors speculate that impaired cell division may be one rare cause of lethal paediatric recessive restrictive cardiomyopathy[133].

Function of KIF20A

KIF20A (Kinesin Family Member 20A) encodes a kinesin, a motor protein which transports cargo from the minus end of microtubules to the plus end (anterograde transport) and transports the Aurora B kinase protein[133], as well as Golgi-associated vesicles. Aurora B is a protein involved in attachment of the mitotic spindle to the centromeres of chromosomes during mitosis, more specifically, it inhibits binding of the spindle microtubules to the kinetochore until there sufficient tension is generated.

PHACTR2

A single study, conducted on an Bedouin family from Israel, associated biallelic *PHACTR2* variation with early-onset DCM with LVNC[135]. The variant associating with disease in the family is the rare, missense allele p.Arg511His and family in question is consanguineous and comprises two heterozygous parents, one affected homozygous male child and his 5 healthy siblings (3 of whom heterozygous for the variant, 2 homozygous for the reference allele). The proband had a dilated LV with decreased function (and normal morphology) at gestation week 32. His birth was normal, but

symptoms of severe heart failure appeared at 10 weeks of age, with subsequent examinations revealing cardiomegaly, pulmonary edema, a severely dilated LV with reduced function (EF 37-40%) and LVNC. As outcome, he is reported alive in his teens (authors don't provide an age indication), under pharmacological treatment and suffering from constant fatigue and exercise intolerance (EF = 44%). Functional follow-up performed by the authors included several experiments. Using the crystal structure of *PHACTR1* (given that that the variant site is conserved across all PHACTR proteins and down to zebrafish) they predicted how the variant would cause misfolding of the second RPEL repeat, which interacts with actin, and would disrupt the entire PHACTR-actin complex. While the variant had no effect on the subcellular localization of the protein, it had significant effects on actin dynamics as observed comparing the proband's fibroblasts to control cells. Among the significant changes observed in the patient's cells were a decreased globular/filamentous actin ratio, an increased accessibility of actin to depolymerizing agents and a decreased repolymerization rate. Furthermore, the proband's cells were dysmorphic (rounded) following actin depolymerization compared with control cells, and a wound healing assay showed control cells to migrate more quickly than the patient's.

Function of PHACTR2

PHACTR2 is a member of a family of four phosphatases and actin regulator (PHACTRs) proteins that exhibit the modulatory activity of protein phosphatase 1 (PP1) and an actin-binding activity[136]. Its expression is ubiquitous, but about twice as high in the heart compared with the skeletal muscle.

RHBDF1

The Al-Hassnan *et al.* cohort study of 205 Saudi Arabian unrelated probands with paediatric cardiomyopathy reported three independent families with a variant in *RHBDF1* (Rhomboid 5 Homolog 1), associating it with paediatric autosomal recessive dilated cardiomyopathy[36]. WES and WGS revealed homozygosity for the p.Gly665Trp *RHBDF1* missense variant in the proband of the first family, and the p.Phe405Serfs*16 frameshift variant in the second and third families. The parents in all three families were consanguineous, heterozygous and unaffected. The boy in the first family presented with DCM at 1 month and was still reported to be alive at 8 years. The boy in the second family presented at 2 months and died at 2.5 years. In the third family, the boy presented at 7 months and was still reported to be alive at 1.67 years. In gnomAD, the frameshift variant is absent, and the missense variant is present at a very rare frequency in heterozygous form (0.00093% in non-Finnish Europeans). The gene is only marginally constrained for loss-of-function variants (LOEUF = 0.94) in gnomAD.

Function of RHBDF1

RHBDF1 encodes the rhomboid 5 homolog 1 protein, otherwise known as the iRhom2 protein (i for inactive). Rhomboid proteases are intramembrane serine proteases—they possess a proteolytic active site which is embedded inside the lipid bilayer. The *RHBDF1* protein does not possess the catalytic serine residue and so is an enzymatically inactive rhomboid protease. Despite this, they still appear to have functions, such as in regulation of the epidermal growth factor receptor signalling pathway[137].

SLC6A6

SLC6A6, encoding for the taurine transporter, was associated with recessive cardiomyopathy in one family in a single study[138]. Two siblings from a consanguineous Pakistani family had mild hypokinetic cardiomyopathy with systolic dysfunction, along with cone-rod retinopathy and were homozygous for the p.Gly399Val variant (detected by WES and homozygosity mapping). Blood taurine levels were almost undetected and functional analysis revealed that transport capacity was only 15% of normal. Taurine supplementation therapy for 24 months completely corrected the cardiomyopathy. Supporting evidence for a role for *SLC6A6* in cardiomyopathy comes from knockout mice studies which demonstrated cardiac dysfunction and fractional shortening in older mice[139]. It is also a putative causal gene at a significant GWAS locus in both HCM and DCM case-control studies[29,140]. Taurine deficiency in dogs and cats also leads to cardiomyopathy, with improvement in echocardiography parameters observed upon diet taurine supplementation[141].

Function of SLC6A6

The *SLC6A6*-encoded taurine transporter is a multi-pass membrane protein and member of the family of sodium and chloride-ion dependent transporters whose function is to transport taurine and beta-alanine.

SOD2

SOD2, encoding for superoxide dismutase 2 or manganese-superoxide dismutase, was associated with recessive DCM in one family in a single study[142]. The p.Gly181Val homozygous variant was identified in a new-born patient (born to distantly related parents) with severe biventricular DCM who died at 4 days. Functional studies confirmed dysfunction of the patient's superoxide dismutase enzyme. While there is only a single family with biallelic *SOD2* variants currently reported, *Sod2* knockout mice can also develop early onset DCM and death (although phenotypes differed depending on strain)[143,144]. *SOD2* is also the putative causal gene at a myocardial fibrosis (interventricular septum and LV free wall) GWAS locus[145].

Function of SOD2

SOD2 is one of three superoxide dismutase genes which convert superoxide anions into hydrogen peroxide and protect cells from damage by reactive oxygen species.

TAF1A

One study reported an association between *TAF1A* (TATA-Box Binding Protein Associated Factor, RNA Polymerase I Subunit A) and recessive DCM[146]. The two daughters of unaffected parents were diagnosed via echocardiography with DCM (LV enlargement; LVEF 20% and 27%), each at 2.5 years of age. The condition of each child deteriorated and they underwent heart transplants at 3 years. WES and subsequent variant filtration revealed compound heterozygosity in both children for the *TAF1A* p.Leu84Ser and p.Gly341Arg missense variants. The parents were heterozygous for one of each of these variants. The gnomAD database shows that these variants are very rare with no homozygotes. Histology of the explanted hearts showed interstitial fibrosis, cardiomyocyte hypertrophy and nucleolar segregation, the latter of which was not present in other DCM hearts—suggesting the involvement of *TAF1A* which is expressed in the nucleolus. Their zebrafish *TAF1A* knockout model showed a heart failure like phenotype; early lethality (all were dead by 11 days post fertilisation);

pericardial oedema; reduction in ventricular fractional shortening; however, there was no mention of cardiac dilatation. An entry exists in ClinVar of a girl (0–9 years old) of African descent (Jamaican) with restrictive cardiomyopathy who is compound heterozygous for the *TAF1A* variants p.Gly341Arg and p.Thr261Pro.

Function of TAF1A

TAF1A encodes the TATA box-binding protein-associated factor RNA polymerase I subunit A protein. The transcription factor SL1, selective factor 1, is composed of one TATA-binding protein (TBP) and three TBF-associated factor proteins (TAFs), one of which is *TAF1A*[147]. SL1 binds to the promoter of ribosomal DNA (rDNA) genes and forms part of the complex of transcription factors which recruit RNA polymerase I (Pol I) and initiate transcription. Pol I transcribes 45S rDNA in the nucleolus. The resulting 45S rRNA is eventually processed and combined with 5S rRNA to form the 80S human ribosome.

ULK1

The Al-Hassnan *et al.* cohort study of 205 Saudi Arabian unrelated probands with paediatric cardiomyopathy reported one family with a variant in *ULK1* (Unc-51 Like Autophagy Activating Kinase 1), associating it with paediatric autosomal recessive DCM[36]. WES revealed homozygosity for the *ULK1* p.Arg691Trp missense variant in the proband. The parents were consanguineous, heterozygous and unaffected. The boy presented at 1.5 months with DCM and died at 2 years of age. In gnomAD, the missense variant is present in heterozygous form but is rare (0.018% in non-Finnish Europeans).

Function of ULK1

ULK1 encodes the Unc-51 like autophagy activating kinase 1 protein. The Atg1/*ULK1* protein-kinase complex is involved in the process of autophagy; specifically in the formation of autophagosomes, which are sealed double-membranes that engulf unwanted proteins and organelles[148]. In the cardiac context, *ULK1* has been associated with protective mitophagy under stress conditions, and double knock-out *ULK1*/*ULK2* perinatal mice develop cardiomyopathy[149–151].

References

1. Almomani, R. *et al.* Biallelic Truncating Mutations in ALPK3 Cause Severe Pediatric Cardiomyopathy. *J Am Coll Cardiol* **67**, 515–25 (2016).

2. Phelan, D. G. *et al.* ALPK3-deficient cardiomyocytes generated from patient-derived induced pluripotent stem cells and mutant human embryonic stem cells display abnormal calcium handling and establish that ALPK3 deficiency underlies familial cardiomyopathy. *Eur Heart J* **37**, 2586–90 (2016).

3. Çağlayan, A. O. *et al.* ALPK3 gene mutation in a patient with congenital cardiomyopathy and dysmorphic features. *Cold Spring Harb Mol Case Stud* **3**, a001859 (2017).

4. Jaouadi, H. *et al.* Novel ALPK3 mutation in a Tunisian patient with pediatric cardiomyopathy and facio-thoraco-skeletal features. *J Hum Genet* **63**, 1077–1082 (2018).

5. Al Senaidi, K. *et al.* Phenotypic spectrum of ALPK3-related cardiomyopathy. *Am J Med Genet A* **179**, 1235–1240 (2019).

6. Herkert, J. C. *et al.* Expanding the clinical and genetic spectrum of ALPK3 variants: Phenotypes identified in pediatric cardiomyopathy patients and adults with heterozygous variants. *Am Heart J* **225**, 108–119 (2020).

7. Jorholt, J. *et al.* Two New Cases of Hypertrophic Cardiomyopathy and Skeletal Muscle Features Associated with ALPK3 Homozygous and Compound Heterozygous Variants. *Genes (Basel)* **11**, 1201 (2020).

8. Papadopoulos, C. *et al.* A novel homozygous ALPK3 variant associated with cardiomyopathy and skeletal muscle involvement. *Muscle Nerve* **65**, E7–E10 (2022).

9. Chumakova, O. S. *et al.* Overlapping Phenotype of Adult-Onset ALPK3-Cardiomyopathy in the Setting of Two Novel Variants. *Cardiol Res* **13**, 398–404 (2022).

10. Lopes, L. R. *et al.* Alpha-protein kinase 3 (ALPK3)-truncating variants are a cause of autosomal dominant hypertrophic cardiomyopathy. *Eur Heart J* **42**, 3063–3073 (2021).

11. Ingles, J. *et al.* Evaluating the Clinical Validity of Hypertrophic Cardiomyopathy Genes. *Circ Genom Precis Med* **12**, e002460 (2019).

12. Tadros, R. *et al.* Shared genetic pathways contribute to risk of hypertrophic and dilated cardiomyopathies with opposite directions of effect. *Nat Genet* **53**, 128–134 (2021).

13. Harper, A. R. *et al.* Common genetic variants and modifiable risk factors underpin hypertrophic cardiomyopathy susceptibility and expressivity. *Nat Genet* **53**, 135–142 (2021).

14. Pirruccello, J. P. *et al.* Analysis of cardiac magnetic resonance imaging in 36,000 individuals yields genetic insights into dilated cardiomyopathy. *Nat Commun* **11**, 2254 (2020).

15. Van Sligtenhorst, I. *et al.* Cardiomyopathy in α-kinase 3 (ALPK3)-deficient mice. *Vet Pathol* **49**, 131–41 (2012).

16. Agarwal, R. *et al.* Pathogenesis of Cardiomyopathy Caused by Variants in ALPK3, an Essential Pseudokinase in the Cardiomyocyte Nucleus and Sarcomere. *Circulation* **146**, 1674–1693 (2022).

17. Hakui, H. *et al.* Loss-of-function mutations in the co-chaperone protein BAG5 cause dilated cardiomyopathy requiring heart transplantation. *Sci Transl Med* **14**, eabf3274 (2022).

18. Lesurf, R. *et al.* Whole genome sequencing delineates regulatory, copy number, and cryptic splice variants in early onset cardiomyopathy. *NPJ Genom Med* **7**, 18 (2022).

19. Aspit, L. *et al.* CAP2 mutation leads to impaired actin dynamics and associates with supraventricular tachycardia and dilated cardiomyopathy. *J Med Genet* **56**, 228–235 (2019).

20. Cheema, H. *et al.* Genomic testing in 1019 individuals from 349 Pakistani families results in high diagnostic yield and clinical utility. *NPJ Genom Med* **5**, 44 (2020).

21. Gurunathan, S. *et al.* A homozygous CAP2 pathogenic variant in a neonate presenting with rapidly progressive cardiomyopathy and nemaline rods. *Am J Med Genet A* **188**, 970–977 (2022).

22. Patel, R. & Peterson, R. Cardiomyopathy presenting prenatally with functional tricuspid and pulmonary atresia. *Echocardiography* **36**, 1779–1782 (2019).

23. Peche, V. S. *et al.* Ablation of cyclase-associated protein 2 (CAP2) leads to cardiomyopathy. *Cell Mol Life Sci* **70**, 527–43 (2013).

24. Field, J. *et al.* CAP2 in cardiac conduction, sudden cardiac death and eye development. *Sci Rep* **5**, 17256 (2015).

25. Stöckigt, F. *et al.* Deficiency of cyclase-associated protein 2 promotes arrhythmias associated with connexin43 maldistribution and fibrosis. *Arch Med Sci* **12**, 188–98 (2016).

26. Kepser, L.-J. *et al.* CAP2 deficiency delays myofibril actin cytoskeleton differentiation and disturbs skeletal muscle architecture and function. *Proc Natl Acad Sci U S A* **116**, 8397–8402 (2019).

27. Al-Hassnan, Z. N. *et al.* A substitution mutation in cardiac ubiquitin ligase, FBXO32, is associated with an autosomal recessive form of dilated cardiomyopathy. *BMC Med Genet* **17**, 3 (2016).

28. Ghasemi, S., Mahdavi, M., Maleki, M., Salahshourifar, I. & Kalayinia, S. A novel likely pathogenic variant in the FBXO32 gene associated with dilated cardiomyopathy according to whole-exome sequencing. *BMC Med Genomics* **15**, 234 (2022).

29. Tadros, R. *et al.* Large scale genome-wide association analyses identify novel genetic loci and mechanisms in hypertrophic cardiomyopathy. *medRxiv* (2023) doi:10.1101/2023.01.28.23285147.

30. Sakaue, S. *et al.* A cross-population atlas of genetic associations for 220 human phenotypes. *Nat Genet* **53**, 1415–1424 (2021).

31. Cárcel-Márquez, J. *et al.* A Polygenic Risk Score Based on a Cardioembolic Stroke Multitrait Analysis Improves a Clinical Prediction Model for This Stroke Subtype. *Front Cardiovasc Med* **9**, 940696 (2022).

32. Roselli, C. *et al.* Multi-ethnic genome-wide association study for atrial fibrillation. *Nat Genet* **50**, 1225–1233 (2018).

33. Nielsen, J. B. *et al.* Biobank-driven genomic discovery yields new insight into atrial fibrillation biology. *Nat Genet* **50**, 1234–1239 (2018).

34. Verweij, N. *et al.* The Genetic Makeup of the Electrocardiogram. *Cell Syst* **11**, 229-238.e5 (2020).

35. Al-Yacoub, N. *et al.* Mutation in FBXO32 causes dilated cardiomyopathy through up-regulation of ER-stress mediated apoptosis. *Commun Biol* **4**, 884 (2021).

36. Al-Hassnan, Z. N. *et al.* Categorized Genetic Analysis in Childhood-Onset Cardiomyopathy. *Circ Genom Precis Med* **13**, 504–514 (2020).

37. Ruijmbeek, C. W. B. *et al.* Bi-allelic variants in FLII cause pediatric cardiomyopathy by disrupting cardiomyocyte cell adhesion and myofibril organization. *JCI Insight* (2023) doi:10.1172/JCI.INSIGHT.168247.

38. Naganawa, Y. & Hirata, H. Developmental transition of touch response from slow muscle-mediated coilings to fast muscle-mediated burst swimming in zebrafish. *Dev Biol* **355**, 194–204 (2011).

39. Campbell, H. D. *et al.* Fliih, a gelsolin-related cytoskeletal regulator essential for early mammalian embryonic development. *Mol Cell Biol* **22**, 3518–26 (2002).

40. Thomsen, N. *et al.* Mouse strains for the ubiquitous or conditional overexpression of the Flii gene. *Genesis* **49**, 681–8 (2011).

41. Vasilescu, C. *et al.* Genetic Basis of Severe Childhood-Onset Cardiomyopathies. *J Am Coll Cardiol* **72**, 2324–2338 (2018).

42. Jones, E. G. *et al.* Analysis of enriched rare variants in JPH2-encoded junctophilin-2 among Greater Middle Eastern individuals reveals a novel homozygous variant associated with neonatal dilated cardiomyopathy. *Sci Rep* **9**, 9038 (2019).

43. Miura, A., Kondo, H., Yamamoto, T., Okumura, Y. & Nishio, H. Sudden Unexpected Death of Infantile Dilated Cardiomyopathy with JPH2 and PKD1 Gene Variants. *Int Heart J* **61**, 1079–1083 (2020).

44. Janin, A. *et al.* Molecular Diagnosis of Primary Cardiomyopathy in 231 Unrelated Pediatric Cases by Panel-Based Next-Generation Sequencing: A Major Focus on Five Carriers of Biallelic TNNI3 Pathogenic Variants. *Mol Diagn Ther* **26**, 551–560 (2022).

45. Mehaney, D. A. *et al.* Molecular analysis of dilated and left ventricular noncompaction cardiomyopathies in Egyptian children. *Cardiol Young* **32**, 295–300 (2022).

46. Jordan, E. *et al.* Evidence-Based Assessment of Genes in Dilated Cardiomyopathy. *Circulation* **144**, 7–19 (2021).

47. Sabater-Molina, M. *et al.* Mutation in JPH2 cause dilated cardiomyopathy. *Clin Genet* **90**, 468–469 (2016).

48. Takeshima, H., Komazaki, S., Nishi, M., Iino, M. & Kangawa, K. Junctophilins: a novel family of junctional membrane complex proteins. *Mol Cell* **6**, 11–22 (2000).

49. Beavers, D. L., Landstrom, A. P., Chiang, D. Y. & Wehrens, X. H. T. Emerging roles of junctophilin-2 in the heart and implications for cardiac diseases. *Cardiovasc Res* **103**, 198–205 (2014).

50. Hedberg-Oldfors, C. *et al.* Cardiomyopathy with lethal arrhythmias associated with inactivation of KLHL24. *Hum Mol Genet* **28**, 1919–1929 (2019).

51. Maurer, C. *et al.* Genetic Insights from Consanguineous Cardiomyopathy Families. *Genes (Basel)* **14**, (2023).

52. Schwieger-Briel, A. *et al.* Epidermolysis Bullosa Simplex with KLHL24 Mutations Is Associated with Dilated Cardiomyopathy. *J Invest Dermatol* **139**, 244–249 (2019).

53. Walsh, R., Offerhaus, J. A., Tadros, R. & Bezzina, C. R. Minor hypertrophic cardiomyopathy genes, major insights into the genetics of cardiomyopathies. *Nat Rev Cardiol* **19**, 151–167 (2022).

54. Vermeer, M. C. *et al.* Gain-of-function mutation in ubiquitin-ligase KLHL24 causes desmin degradation and dilatation in hiPSC-derived engineered heart tissues. *J Clin Invest* (2021) doi:10.1172/JCI140615.

55. Koopmann, T. T. *et al.* Biallelic loss of LDB3 leads to a lethal pediatric dilated cardiomyopathy. *Eur J Hum Genet* **31**, 97–104 (2023).

56. Zhou, Q. *et al.* Ablation of Cypher, a PDZ-LIM domain Z-line protein, causes a severe form of congenital myopathy. *J Cell Biol* **155**, 605–12 (2001).

57. Zheng, M. *et al.* Cardiac-specific ablation of Cypher leads to a severe form of dilated cardiomyopathy with premature death. *Hum Mol Genet* **18**, 701–13 (2009).

58. Cheng, H. *et al.* Selective deletion of long but not short Cypher isoforms leads to late-onset dilated cardiomyopathy. *Hum Mol Genet* **20**, 1751–62 (2011).

59. van der Meer, D. L. M. *et al.* Zebrafish cypher is important for somite formation and heart development. *Dev Biol* **299**, 356–72 (2006).

60. Ntalla, I. *et al.* Multi-ancestry GWAS of the electrocardiographic PR interval identifies 202 loci underlying cardiac conduction. *Nat Commun* **11**, 2542 (2020).

61. Abdelfatah, N. *et al.* Characterization of a Unique Form of Arrhythmic Cardiomyopathy Caused by Recessive Mutation in LEMD2. *JACC Basic Transl Sci* **4**, 204–221 (2019).

62. Boone, P. M. *et al.* Hutterite-type cataract maps to chromosome 6p21.32-p21.31, cosegregates with a homozygous mutation in LEMD2, and is associated with sudden cardiac death. *Mol Genet Genomic Med* **4**, 77–94 (2016).

63. Tapia, O., Fong, L. G., Huber, M. D., Young, S. G. & Gerace, L. Nuclear envelope protein Lem2 is required for mouse development and regulates MAP and AKT kinases. *PLoS One* **10**, e0116196 (2015).

64. Chen, R. *et al.* Mechanistic Insights of the LEMD2 p.L13R Mutation and Its Role in Cardiomyopathy. *Circ Res* (2023) doi:10.1161/CIRCRESAHA.122.321929.

65. Caravia, X. M. *et al.* Loss of function of the nuclear envelope protein LEMD2 causes DNA damage-dependent cardiomyopathy. *J Clin Invest* **132**, (2022).

66. von Appen, A. *et al.* LEM2 phase separation promotes ESCRT-mediated nuclear envelope reformation. *Nature* **582**, 115–118 (2020).

67. Brachner, A., Reipert, S., Foisner, R. & Gotzmann, J. LEM2 is a novel MAN1-related inner nuclear membrane protein associated with A-type lamins. *J Cell Sci* **118**, 5797–810 (2005).

68. Ahrens-Nicklas, R. C. *et al.* Disruption of cardiac thin filament assembly arising from a mutation in LMOD2: A novel mechanism of neonatal dilated cardiomyopathy. *Sci Adv* **5**, eaax2066 (2019).

69. Greenway, S. C. *et al.* Early Death of 2 Siblings Related to Mutations in LMOD2, a Recently Discovered Cause of Neonatal Dilated Cardiomyopathy. *CJC Open* **3**, 1300–1302 (2021).

70. Yuen, M. *et al.* Neonatal-lethal dilated cardiomyopathy due to a homozygous LMOD2 donor splice-site variant. *Eur J Hum Genet* **30**, 450–457 (2022).

71. Lay, E. *et al.* LMOD2-related dilated cardiomyopathy presenting in late infancy. *Am J Med Genet A* **188**, 1858–1862 (2022).

72. Sono, R. *et al.* Whole-Exome Sequencing Identifies Homozygote Nonsense Variants in LMOD2 Gene Causing Infantile Dilated Cardiomyopathy. *Cells* **12**, (2023).

73. Pappas, C. T. *et al.* Knockout of Lmod2 results in shorter thin filaments followed by dilated cardiomyopathy and juvenile lethality. *Proc Natl Acad Sci U S A* **112**, 13573–8 (2015).

74. Tolkatchev, D. *et al.* Leiomodin creates a leaky cap at the pointed end of actin-thin filaments. *PLoS Biol* **18**, e3000848 (2020).

75. Heliö, K. *et al.* GRINL1A Complex Transcription Unit Containing GCOM1, MYZAP, and POLR2M Genes Associates with Fully Penetrant Recessive Dilated Cardiomyopathy. *Front Genet* **12**, 786705 (2021).

76. Maver, A. *et al.* A biallelic loss-of-function variant in MYZAP is associated with a recessive form of severe dilated cardiomyopathy. *Cold Spring Harb Mol Case Stud* **8**, (2022).

77. Seeger, T. S. *et al.* Myozap, a novel intercalated disc protein, activates serum response factor-dependent signaling and is required to maintain cardiac function in vivo. *Circ Res* **106**, 880–90 (2010).

78. Rangrez, A. Y. *et al.* Myozap Deficiency Promotes Adverse Cardiac Remodeling via Differential Regulation of Mitogen-activated Protein Kinase/Serum-response Factor and β-Catenin/GSK-3β Protein Signaling. *J Biol Chem* **291**, 4128–43 (2016).

79. Thorolfsdottir, R. B. *et al.* Coding variants in RPL3L and MYZAP increase risk of atrial fibrillation. *Commun Biol* **1**, 68 (2018).

80. Monies, D. *et al.* The landscape of genetic diseases in Saudi Arabia based on the first 1000 diagnostic panels and exomes. *Hum Genet* **136**, 921–939 (2017).

81. Truszkowska, G. T. *et al.* Homozygous truncating mutation in NRAP gene identified by whole exome sequencing in a patient with dilated cardiomyopathy. *Sci Rep* **7**, 3362 (2017).

82. Koskenvuo, J. W. *et al.* Biallelic loss-of-function in NRAP is a cause of recessive dilated cardiomyopathy. *PLoS One* **16**, e0245681 (2021).

83. Ahmed, H., Al-ghamdi, S. & Mutairi, F. Dilated cardiomyopathy in a child with truncating mutation in NRAP gene. *Journal of Biochemical and Clinical Genetics* **1**, 77–80 (2018).

84. Bihlmeyer, N. A. *et al.* ExomeChip-Wide Analysis of 95 626 Individuals Identifies 10 Novel Loci Associated With QT and JT Intervals. *Circ Genom Precis Med* **11**, e001758 (2018).

85. Ehler, E. *et al.* Alterations at the intercalated disk associated with the absence of muscle LIM protein. *J Cell Biol* **153**, 763–72 (2001).

86. Perriard, J.-C., Hirschy, A. & Ehler, E. Dilated cardiomyopathy: a disease of the intercalated disc? *Trends Cardiovasc Med* **13**, 30–8 (2003).

87. Lu, S. *et al.* Cardiac-specific NRAP overexpression causes right ventricular dysfunction in mice. *Exp Cell Res* **317**, 1226–37 (2011).

88. Muhammad, E. *et al.* PLEKHM2 mutation leads to abnormal localization of lysosomes, impaired autophagy flux and associates with recessive dilated cardiomyopathy and left ventricular noncompaction. *Hum Mol Genet* **24**, 7227–40 (2015).

89. Atkins, J. *et al.* PLEKHM2 Loss-of-Function Is Associated With Dilated Cardiomyopathy. *Circ Genom Precis Med* **15**, e003594 (2022).

90. Rosa-Ferreira, C. & Munro, S. Arl8 and SKIP act together to link lysosomes to kinesin-1. *Dev Cell* **21**, 1171–8 (2011).

91. Kennedy, H. *et al.* Sudden Cardiac Death Due to Deficiency of the Mitochondrial Inorganic Pyrophosphatase PPA2. *Am J Hum Genet* **99**, 674–682 (2016).

92. Guimier, A. *et al.* Biallelic PPA2 Mutations Cause Sudden Unexpected Cardiac Arrest in Infancy. *Am J Hum Genet* **99**, 666–673 (2016).

93. Phoon, C. K. L. *et al.* Sudden unexpected death in asymptomatic infants due to PPA2 variants. *Mol Genet Genomic Med* **8**, e1008 (2020).

94. Guimier, A. *et al.* PPA2-associated sudden cardiac death: extending the clinical and allelic spectrum in 20 new families. *Genet Med* **23**, 2415–2425 (2021).

95. Manzanilla-Romero, H. H., Schermer, E., Mayr, A. & Rudnik-Schöneborn, S. Only one beer can be mortal: a case report of two sisters with cardiac arrest due to a homozygous mutation in PPA2 gene. *Eur J Pediatr* (2023) doi:10.1007/s00431-023-05034-9.

96. Falik-Zaccai, T. C. *et al.* Sequence variation in PPP1R13L results in a novel form of cardio-cutaneous syndrome. *EMBO Mol Med* **9**, 319–336 (2017).

97. Robinson, H. K. *et al.* Biallelic variants in PPP1R13L cause paediatric dilated cardiomyopathy. *Clin Genet* **98**, 331–340 (2020).

98. Henry, A., Bernhardt, I., Hayes, I. & Mitchelson, B. Novel PPP1R13L variant expands the phenotype of a rare cardiocutaneous syndrome. *Clin Genet* **102**, 461–462 (2022).

99. Kalayinia, S. *et al.* Novel homozygous stop-gain pathogenic variant of PPP1R13L gene leads to arrhythmogenic cardiomyopathy. *BMC Cardiovasc Disord* **22**, 359 (2022).

100. Bagnall, R. D. *et al.* Genetic Basis of Childhood Cardiomyopathy. *Circ Genom Precis Med* **15**, e003686 (2022).

101. Herron, B. J. *et al.* A mutation in NFkB interacting protein 1 results in cardiomyopathy and abnormal skin development in wa3 mice. *Hum Mol Genet* **14**, 667–77 (2005).

102. Toonen, J., Liang, L. & Sidjanin, D. J. Waved with open eyelids 2 (woe2) is a novel spontaneous mouse mutation in the protein phosphatase 1, regulatory (inhibitor) subunit 13 like (Ppp1r13l) gene. *BMC Genet* **13**, 76 (2012).

103. Ganapathi, M. *et al.* Bi-allelic missense disease-causing variants in RPL3L associate neonatal dilated cardiomyopathy with muscle-specific ribosome biogenesis. *Hum Genet* **139**, 1443–1454 (2020).

104. Nannapaneni, H. *et al.* Further Evidence of Autosomal Recessive Inheritance of RPL3L Pathogenic Variants with Rapidly Progressive Neonatal Dilated Cardiomyopathy. *J Cardiovasc Dev Dis* **9**, (2022).

105. Das, B. B., Gajula, V., Arya, S. & Taylor, M. B. Compound Heterozygous Missense Variants in RPL3L Genes Associated with Severe Forms of Dilated Cardiomyopathy: A Case Report and Literature Review. *Children (Basel)* **9**, (2022).

106. Kurki, M. I. *et al.* FinnGen provides genetic insights from a well-phenotyped isolated population. *Nature* **613**, 508–518 (2023).

107. Weng, L.-C. *et al.* Genetic Determinants of Electrocardiographic P-Wave Duration and Relation to Atrial Fibrillation. *Circ Genom Precis Med* **13**, 387–395 (2020).

108. Chaillou, T., Zhang, X. & McCarthy, J. J. Expression of Muscle-Specific Ribosomal Protein L3-Like Impairs Myotube Growth. *J Cell Physiol* **231**, 1894–902 (2016).

109. Lieberwirth, J. K. *et al.* Bi-allelic loss of function variants in SLC30A5 as cause of perinatal lethal cardiomyopathy. *Eur J Hum Genet* **29**, 808–815 (2021).

110. Inoue, K. *et al.* Osteopenia and male-specific sudden cardiac death in mice lacking a zinc transporter gene, Znt5. *Hum Mol Genet* **11**, 1775–84 (2002).

111. Hara, T. *et al.* Physiological roles of zinc transporters: molecular and genetic importance in zinc homeostasis. *J Physiol Sci* **67**, 283–301 (2017).

112. Salazar-Mendiguchía, J. *et al.* Mutations in TRIM63 cause an autosomal-recessive form of hypertrophic cardiomyopathy. *Heart* **106**, 1342–1348 (2020).

113. Su, M. *et al.* Rare variants in genes encoding MuRF1 and MuRF2 are modifiers of hypertrophic cardiomyopathy. *Int J Mol Sci* **15**, 9302–13 (2014).

114. Allouba, M. *et al.* Ethnicity, consanguinity, and genetic architecture of hypertrophic cardiomyopathy. *Eur Heart J* (2023) doi:10.1093/EURHEARTJ/EHAD372.

115. Jokela, M., Baumann, P., Huovinen, S., Penttilä, S. & Udd, B. Homozygous Nonsense Mutation p.Q274X in TRIM63 (MuRF1) in a Patient with Mild Skeletal Myopathy and Cardiac Hypertrophy. *J Neuromuscul Dis* **6**, 143–146 (2019).

116. Olivé, M. *et al.* New cardiac and skeletal protein aggregate myopathy associated with combined MuRF1 and MuRF3 mutations. *Hum Mol Genet* **24**, 3638–50 (2015).

117. van der Harst, P. *et al.* 52 Genetic Loci Influencing Myocardial Mass. *J Am Coll Cardiol* **68**, 1435–1448 (2016).

118. Witt, C. C. *et al.* Cooperative control of striated muscle mass and metabolism by MuRF1 and MuRF2. *EMBO J* **27**, 350–60 (2008).

119. Willis, M. S. *et al.* Muscle ring finger 1, but not muscle ring finger 2, regulates cardiac hypertrophy in vivo. *Circ Res* **100**, 456–9 (2007).

120. Abu-Elheiga, L., Matzuk, M. M., Abo-Hashema, K. A. & Wakil, S. J. Continuous fatty acid oxidation and reduced fat storage in mice lacking acetyl-CoA carboxylase 2. *Science* **291**, 2613–6 (2001).

121. Luo, K. *et al.* Identification and functional characterization of BICD2 as a candidate disease gene in an consanguineous family with dilated cardiomyopathy. *BMC Med Genomics* **15**, 189 (2022).

122. Martinez-Carrera, L. A. & Wirth, B. Dominant spinal muscular atrophy is caused by mutations in BICD2, an important golgin protein. *Front Neurosci* **9**, 401 (2015).

123. Matanis, T. *et al.* Bicaudal-D regulates COPI-independent Golgi-ER transport by recruiting the dynein-dynactin motor complex. *Nat Cell Biol* **4**, 986–92 (2002).

124. Teuling, E. *et al.* A novel mouse model with impaired dynein/dynactin function develops amyotrophic lateral sclerosis (ALS)-like features in motor neurons and improves lifespan in SOD1-ALS mice. *Hum Mol Genet* **17**, 2849–62 (2008).

125. Liu, Z. *et al.* Essential role of the zinc finger transcription factor Casz1 for mammalian cardiac morphogenesis and development. *J Biol Chem* **289**, 29801–16 (2014).

126. Young, W. J. *et al.* Genetic analyses of the electrocardiographic QT interval and its components identify additional loci and pathways. *Nat Commun* **13**, 5144 (2022).

127. Christine, K. S. & Conlon, F. L. Vertebrate CASTOR is required for differentiation of cardiac precursor cells at the ventral midline. *Dev Cell* **14**, 616–23 (2008).

128. Theis, J. L. *et al.* Homozygosity mapping and exome sequencing reveal GATAD1 mutation in autosomal recessive dilated cardiomyopathy. *Circ Cardiovasc Genet* **4**, 585–94 (2011).

129. Yang, J., Shah, S., Olson, T. M. & Xu, X. Modeling GATAD1-Associated Dilated Cardiomyopathy in Adult Zebrafish. *J Cardiovasc Dev Dis* **3**, (2016).

130. Vermeulen, M. *et al.* Quantitative interaction proteomics and genome-wide profiling of epigenetic histone marks and their readers. *Cell* **142**, 967–80 (2010).

131. Kaneda, R. *et al.* Genome-wide histone methylation profile for heart failure. *Genes Cells* **14**, 69–77 (2009).

132. Verhagen, J. M. A. *et al.* Biallelic Variants in ASNA1, Encoding a Cytosolic Targeting Factor of Tail-Anchored Proteins, Cause Rapidly Progressive Pediatric Cardiomyopathy. *Circ Genom Precis Med* **12**, 397–406 (2019).

133. Louw, J. J. *et al.* Compound heterozygous loss-of-function mutations in KIF20A are associated with a novel lethal congenital cardiomyopathy in two siblings. *PLoS Genet* **14**, e1007138 (2018).

134. Dickinson, M. E. *et al.* High-throughput discovery of novel developmental phenotypes. *Nature* **537**, 508–514 (2016).

135. Majdalani, P. *et al.* A Missense Variation in PHACTR2 Associates with Impaired Actin Dynamics, Dilated Cardiomyopathy, and Left Ventricular Non-Compaction in Humans. *Int J Mol Sci* **24**, (2023).

136. Allen, P. B., Greenfield, A. T., Svenningsson, P., Haspeslagh, D. C. & Greengard, P. Phactrs 1-4: A family of protein phosphatase 1 and actin regulatory proteins. *Proc Natl Acad Sci U S A* **101**, 7187–92 (2004).

137. Burzenski, L. M. *et al.* Inactive rhomboid proteins RHBDF1 and RHBDF2 (iRhoms): a decade of research in murine models. *Mamm Genome* **32**, 415–426 (2021).

138. Ansar, M. *et al.* Taurine treatment of retinal degeneration and cardiomyopathy in a consanguineous family with SLC6A6 taurine transporter deficiency. *Hum Mol Genet* **29**, 618–623 (2020).

139. Ito, T. *et al.* Cardiac and skeletal muscle abnormality in taurine transporter-knockout mice. *J Biomed Sci* **17 Suppl 1**, S20 (2010).

140. Garnier, S. *et al.* Genome-wide association analysis in dilated cardiomyopathy reveals two new players in systolic heart failure on chromosomes 3p25.1 and 22q11.23. *Eur Heart J* **42**, 2000–2011 (2021).

141. Kaplan, J. L. *et al.* Taurine deficiency and dilated cardiomyopathy in golden retrievers fed commercial diets. *PLoS One* **13**, e0209112 (2018).

142. Almomani, R. *et al.* Homozygous damaging SOD2 variant causes lethal neonatal dilated cardiomyopathy. *J Med Genet* **57**, 23–30 (2020).

143. Li, Y. *et al.* Dilated cardiomyopathy and neonatal lethality in mutant mice lacking manganese superoxide dismutase. *Nat Genet* **11**, 376–81 (1995).

144. Lebovitz, R. M. *et al.* Neurodegeneration, myocardial injury, and perinatal death in mitochondrial superoxide dismutase-deficient mice. *Proc Natl Acad Sci U S A* **93**, 9782–7 (1996).

145. Nauffal, V. *et al.* Genetics of myocardial interstitial fibrosis in the human heart and association with disease. *Nat Genet* **55**, 777–786 (2023).

146. Long, P. A. *et al.* Recessive TAF1A mutations reveal ribosomopathy in siblings with end-stage pediatric dilated cardiomyopathy. *Hum Mol Genet* **26**, 2874–2881 (2017).

147. Comai, L. *et al.* Reconstitution of transcription factor SL1: exclusive binding of TBP by SL1 or TFIID subunits. *Science* **266**, 1966–72 (1994).

148. Tanida, I. Autophagosome formation and molecular mechanism of autophagy. *Antioxid Redox Signal* **14**, 2201–14 (2011).

149. Tong, M. *et al.* Alternative Mitophagy Protects the Heart Against Obesity-Associated Cardiomyopathy. *Circ Res* **129**, 1105–1121 (2021).

150. Nah, J. *et al.* Ulk1-dependent alternative mitophagy plays a protective role during pressure overload in the heart. *Cardiovasc Res* **118**, 2638–2651 (2022).

151. Harris, M. P. *et al.* Perinatal versus adult loss of ULK1 and ULK2 distinctly influences cardiac autophagy and function. *Autophagy* **18**, 2161–2177 (2022).
